# Europe and Turkey: identities in evolution. An analytical literature review

**DOI:** 10.12688/openreseurope.16176.1

**Published:** 2023-07-26

**Authors:** Seckin Baris Gulmez, Alp Eren Topal, Bahar Rumelili

**Affiliations:** 1 Department of International Relations, Izmir Katip Celebi University, Izmir, Turkey; 2 Department of Political and Social Sciences, Free University of Berlin, Berlin, Germany; 3 Department of International Relations, Koç University, Istanbul, Turkey

**Keywords:** Turkey, Europe, Identity, Culture

## Introduction

This article provides a comprehensive analytical review of the existing literature on identity relations between Turkey and Europe. How Turks and Europeans perceive each other has a direct impact on their attitudes towards and decisions about each other. Therefore, Turkey’s status in Europe and Europe’s image in Turkey have long been the focus of political as well as academic debates. However, there is an evident lack of scholarly attention on the evolution of mutual representations comparatively in the *long durée*. Most scholarly works focus on specific periods of time and investigate either how Turks view Europe or visa-versa without taking into account the mutual interaction between identity repesentations. This analytical review synthesizes the findings of these disparate studies to provide the basis for an evolutionary analysis of mutual identity representations between the two sides over more than a two-hundred-year period.

The period under focus starts with the French Revolution (1789) triggering intensive identity debates between Ottomans and Europeans and lasts until mid-2010s when bilateral relations between Turkey and the European Union (EU) went into disarray. This long period has been divided into four shorter periods each starting and ending with a remarkable event in world politics or bilateral relations. This periodization facilitates temporal comparison in tracing continuity and change in identity representations.

The first period (1789–1922) commences with the final century of the Ottoman Empire, in which key political developments in both the Ottoman Empire and Europe shaped identity representations of each other on both sides. The second period (1923–1945) starts with the establishment of the Turkish Republic ushering in substantial political and socio-cultural changes and ends with the Second World War. This period not only witnessed a rapid transformation of Turkey from empire to nation state, but also constitutes a turbulent age in European history leading up to the Second World War.

The third period (1946–1998) starts with the immediate aftermath of the Second World War, proceeds with the Cold War, and finalizes with the first decade of the post-Cold War era. This period has substantial ramifications for Turkey and Europe as allies during the Cold War and the EU-Turkey relationship in the post-Cold War era. The fourth and the final period (1999–2016) covers a comparably shorter time frame which nevertheless had a crucial bearing upon Turkey’s EU membership. It starts with the official recognition of Turkey’s EU candidacy at the Helsinki European Council Summit (11–12 Decmber 1999) and lasts until the Turkey-EU joint refugee deal on 18 March 2016, the final remarkable event before the bilateral relations went into impasse.

The study examines each period in relation to selected focal issues, namely the issues with respect to which Europe (or Turkey) constitutes its identity by comparing itself with and/or differentiating itself from its significant Other, i.e., Turkey (or Europe). The four focal issues identified are nationalism, civilization, status in international society, and state-citizen relations generating intensive identity discussions in both sides.

This is the first review of its kind surveying the vast literature on Turkey-Europe identity relations across the four historical periods. The relevant literature on identity relations between Turkey and Europe published in the English and Turkish languages was identified through the search of keywords ‘Turkey’, ‘Europe’, ‘Ottoman Empire’, ‘identity’, ‘image’, and ‘culture’ in Koc and Sabanci University databases. In addition, keywords involving the focal issues were used where appropriate. This system ensured that all academic literature involving these keywords appeared in the literature review, and also served as a means to distinguish the literature focusing on identity relations from those focusing on other aspects of Turkey-Europe relations in different time periods. The bibliography provides a comprehensive listing of the 313 works analyzed for the purposes of this review.

The following sections will present the key findings of the relevant literature on European representations of Turkey and on Turkish representations of Europe in the four historical periods. Then it will analyze the relevant literature on each period with respect to the four focal issues. Overall, the study, through a comprehensive literature review, demonstrates the ways in which mutual identity representations in Turkey and Europe have been contested over a two-hundred-year period.

## The First Period (1789–1922)

The period between 1789 and 1922 covers the last 150 years of the Ottoman Empire and is marked by a constant effort by Ottoman bureaucrats and government to modernize the administration, the economy and the society of the Empire and salvage the Empire from total collapse. The period starts with the French Revolution and ends with the fall of the Ottoman Empire and the founding of the Turkish republic.

The period starts with the French Revolution, which, despite having little impact in the Ottoman Empire initially, was synchronous with the first extensive reform program in the history of the Empire, namely the New Order (1791–1808). The New Order program included the first attempts by Ottomans to adopt European military technologies and asu such it provoked discussions of Westernization and identity. Though the New Order program failed, Ottoman state engaged in successive comprehensive reform initiatives such as the proclamation of the Tanzimat (1839) and the Reform Edict (1856), both of which coincided with two major international crises: Egyptian challenge to Ottoman security in the 1830s which involved Russia and European governments in a game of power and influence, and the Crimean War (1853–56) which ended with an alliance of British, French and Ottoman armies against Russia. These crises and synchronous reform initiatives were core drivers which relate directly to the four focal issues of our project.

After a very brief experiment with constitutional monarchy in 1876, Abdulhamid II dismissed the parliament and Ottoman Empire entered a 30-year absolutist rule which is associated with the rise of pan-Islamism as an ideology in response to the legitimacy crisis of the Ottoman dynasty and government and wider developments in the world. Hamidian era also brought large scale infrastructural modernization, dissemination of public education in the Empire and the rise of a new educated bureaucratic and military elite which eventually brought down Abdulhamid and initiated the Second Constitutional period in 1908 under the banner of the Committee of Union and Progress (CUP). The 15 years of CUP government witnessed the two major and catastrophic events which had a decisive impact on the identity issueand sealed the image of the Turk abroad: The Balkan Wars (1912–13) which set the stage for the WW I and Armenian Genocide (1915).

In terms of cultural developments, the period witnessed several major cultural shifts in the Ottoman Empire. Starting with the fashioning of the Ottoman bureaucratic system in the European style and the establishment of the translation bureau, Westernization in urban social and intellectual life spread gradually and geographically from Istanbul to other urban centers. Translations were made from European literature starting from 1850s and Ottoman writers started experimenting with European genres of novel and poetic styles. Consumption patterns and leisure activities as well as architectural style and education imitated European models to varying degrees among the emerging Ottoman middle classes.

The literature on respective Ottoman and European representations of each other consist mostly of historical scholarship, involving in depth studies of particular events or a genre of texts. The majority of the scholars are students of Middle Eastern history and Ottoman studies and, hence, going by the standards of the field they draw on a wide range of disciplinary approaches from literary criticism to sociology to media studies. The available literature is still overwhelmingly driven by the choice of available material rather than theoretical or methodological concerns. Scholars use archival material (i.e. newspaper articles, archival documents, diplomatic correspondences, letters, popular publications, and visual material such as photographs and cartoons) to identify various representations of the Europe and the Ottoman Empire. As such, the literature overall lacks a dominant or driving methodology; virtually all of the studies rely on close or selective reading of certain textual material with reference to context with the exception of Einar Wigen’s work on the concept of Europe in the late Ottoman Empire and republican Turkey (2010), which employs discourse analysis and conceptual study with a careful attention to theories of identity and processes of othering. There are very few systematic studies covering the whole period using extensive samples, such as Cemil Aydın’s comparative study of anti-Westernism, pan-Islamism and Pan-Asianism in the Ottoman Empire and Japan respectively (2007), Şerif Mardin’s analysis of the criticism of over-Westernization in the Ottoman Empire throughout the 19 ^th^ century literature (1974), Doğan Gürpınar’s article on British turcophilism throughout the 19 ^th^ century (2012) and Chase Gummer’s unpublished dissertation on Turcophilism in German press from 1850s to the WW I (2010). Beyond these, the bulk of the literature deals with a particular genre such as travel writing or photography ( Baleva, 2012; Demiraj, 2001; Şirin, 2009) or a very limited period ( Brummet, 2000; Çiçek, 2010; Köroğlu, 2007).

The literature on the Ottoman perspectives on Europe and its identity dimension during the nineteenth century mainly focus on the tensions of Ottoman reform process: the desire to become a part of the post-Vienna European order and the formation of national identity which gradually acquires an anti-Westernist tone. Westernization appears as both a desirable phenomenon which involves acquisition of military administrative technology, and also as a social ill which manifests in consumption patterns, material culture and alienation. The literature on European perspective, on the other hand, focus mainly on two things: orientalist representations of the East as exotic and backward at the same time and Eastern question, the problem of the fate of the Ottoman Empire and its multi-confessional subjects in the face of Russian threat and subsequent nationalist uprisings particularly in the Balkans. Particularly the press coverage of successive periods of violence in the Ottoman Empire between the state, Muslim subjects and the non-Muslim minorities have received scholarly attention. The Crimean War (1853–56), the Bulgarian uprisings of 1875 and the ensuing Russo-Ottoman War, the massacre of Armenians in 1890s and the Balkan upheavals between 1902 and 1913 seem to be significant turning points that shaped European representation of the Ottoman Empire and “the Turk”.

A significant difference between the literature on European representation and the Ottoman ones is the sources used. While the former draws on a wide range of sources from diplomatic letters to daily newspapers and popular literature thus covering both the elite and public perceptions ( Baleva, 2012; Demiraj, 2001; Gummer, 2010; Gürpınar, 2012), lack of an independent and widespread reading public in the Ottoman Empire for the better part of the 19 ^th^ century (until 1908 to be precise) seems to have narrowed the scope of studies to writings of bureaucrats and several elect intellectuals who were, again, active or former bureaucrats ( Aydın, 2007; Baykara, 2007; Çiçek, 2010; Şirin, 2009; Wigen, 2010), thus limiting the representative value of the conclusions drawn; only dealing with larger public in the second constitutional period ( Brummet, 2000; Köroğlu, 2007). There is an observable emphasis on British views on the Empire, probably owing to the accessibility of primary sources and preference for English as the language of scholarship ( Çiçek, 2010; Çırakman, 2001; Demiraj, 2001; Gürpınar, 2012). German perspectives on the Empire follow the literature on British ( Gummer, 2010; Vettes, 1958); however, there is hardly anything specific on French culture. It should be mentioned, however, this review deals exclusively with the literature in English due to practical limitations and secondary literature in German and French are covered only so far as they are used by the literature in English (see particularly work of Gummer, 2010).

### European representations of the Ottoman Empire

It is generally observed in the literature that the image of the Ottoman Empire in Europe shifts from a more positive one to a more negative one during the eighteenth century and the Enlightenment. Both Aslı Çırakman’s ( 2001; 2002) and M.E. Yapp’s (1992) work on the shifting European image of the Turks from the sixteenth to the nineteenth centuries based on a variety of European authors argue that while Ottoman state and society was associated with the concept “tyranny” earlier, during the nineteenth century this is replaced with “despotism.” Çırakman (2001:50) observes that while “tyranny" denoted “both positive and negative features… ‘despotism’ had no redeeming feature”. Drawing on essentialist assumptions based on climate and geography European authors began to perceive an irredeemable difference between the “Orient” and “Europe.” Revealing a “European self-image as indisputably civilized, progressive, liberal, and rational,” this perspective posited the Ottoman Empire as despotic, irrational, backward, fatalistic and stagnant ( Çırakman, 2001: 64; Yapp, 1992: 149–151). This shift in representations is confirmed by Edward Said’s monumental work on Orientalism which demonstrated the asymmetric power relations, that is European imperialism behind the particularly nineteenth century European Orientalist discourse which depicted the Orient as its negative “other” ( Gürpınar, 2012). Çırakman concludes that this negative and prejudiced image was a product of assumptions inherent in the Enlightenment discourse and Yapp emphasizes the significance of gradual secularization and the emergence of the idea of moral and material progress and civilization as central to European identity which reflects negatively on the Ottoman Empire ( Yapp, 1992: 152–153).

Felix Konrad (2011: 30) also confirms this perspective and observes that by the eighteenth-century Ottoman Empire was being systematically excluded from conceptions of Europe: Orient and Occident were being imagined as “irreconcilable and contrary civilizations” under secular criteria. Konrad (2011: 31) notes how Napoleon’s invasion of Egypt was justified in France by claiming that they were saving the Egyptians from the “yoke” of the despotic Ottomans. Herder saw Ottomans as “strangers” to Europe, whereas Hegel discarded the whole Orient and Islamic East as stagnant immobile and hence relevant only so far as they helped carry the ancient knowledge to Europe (ibid: 32). Thus, Ottoman Empire entered the nineteenth century bearing a dominantly negative image in the eyes of European public, which was further exacerbated by the Greek revolt in 1820s, which appealed to the philhellenism prevalent in Europe and once again reinforced the image of the Turks as “barbaric and un-European” (ibid: 33). Particularly the literary writings of Lord Byron helped create a highly eroticized image of the Orient which was essentially different from Europe ( Renda, 2006: 18). Byron invoked support for the Greeks and Delacroix’s famous painting *Massacres in Chiox* was actually based on Byron’s work (ibid). Renda (2006: 18) observes that the romantic image of the Ottoman Empire in the early nineteenth century was so exotic that European orientalists were initially disappointed with the Westernizing reforms in the Empire for destroying the authenticity of the Ottoman geography.

Voltisa Demiraj (2001), in her study of John Henry Newman (d. 1890), a prominent British catholic intellectual, analyzes the late eighteenth and early nineteenth century European travelers and scholars, on whose works Newman’s lectures on the history of the Turks was based. Newman, who was a conservative Christian, wrote his history in an effort to influence public opinion against British alliance with Ottoman Empire against Russia in 1853 leading to the Crimean War. As a conservative, Newman was not fond of concepts such as civilization and progress which was championed by the liberals, however his biggest argument against the Turks was that they were uncivilized, barbaric and incapable of progress, which, according to Demiraj, still showed how these concepts were central to British self-perception and the Muslim other. Though Newman highly valued dogma as a Catholic, he had a very low opinion of Islam which he associated with apathy and decay as opposed to Christianity (ibid: 88). He also did not think it proper for a Christian nation to ally itself with Muslims against Christians although Russia belonged to the Eastern Orthodox Church. Demiraj (2001: 74–75) observes that while Newman was eclectic in his use of the sources and sometimes neglected the evidence which opposed his argument, still most of the literature he relied on had a dominantly negative view of the Turks as savage, barbaric, cruel, rude and ignorant. Although some travelers saw contradictorily positive qualities in Turks as well, such as hospitality, bravery, sensuality and delicacy, the ultimate verdict was still that they were savage (ibid: 76–77). However, Demiraj (2001: 35) also notes that there was a Turcophile section within the British parliament which considered Russia as evil and Turks as good, and Orthodox as superstitious and Moslems as progressive. Thus, the image of the Turk was inextricably linked with the identity struggles within the British society between protestant liberals and conservative segments.

Doğan Gürpınar (2012: 349), in his article on the nineteenth century British Turcophilism, emphasizes the role of British fear of Russia’s growing power and the Westernizing reforms of the Ottoman government, which led to the emergence of an alternative image of the Turk as a promising candidate to the newly established European order. Several French and British writers saw a great ruler and reformer in Mahmud II (ibid: 352) and efforts of David Urquhart, a Russophobe and a Turcophile in the parliament was influential in promoting a positive image of the Turk during the 1830s and 40s (ibid: 355 and Çiçek, 2010: 50–57). As a romantic Urquhart was enamored with the pre-Tanzimat Turk and his authenticity while at the same time he vehemently supported the Tanzimat reforms. Gürpınar (2012: 357) also notes how the most common cliché regarding the Ottoman Empire was to imagine a contrast between the enlightened elite and “ignorant and barbaric Oriental flock”. Somewhat contradicting Demiraj’s conclusion, Gürpınar observes that Turcophilism was consistently correlated with conservatism and romanticism in Britain through the nineteenth century; liberals supported Turkish cause only so far as it suited their foreign policy objectives.

However, this positive attitude which peaked during the Crimean War during the 1850s began to erode in 1860s both in France and Britain due to unresolved ethnic conflicts within the Empire and growing foreign debt of the government ( Gürpınar, 2012: 350). A particularly crucial turning point for the British support for the Ottoman Empire seems to have been the unrest in the Balkans which erupted in 1876 and triggered a major war between Russia and the Ottoman Empire. The uprising and its suppression by the Ottoman army was covered extensively in European and particularly British press. The leader of the British opposition William Gladstone’s pamphlet *Bulgarian Horrors and the Question of the East* was an instant bestseller. Newspapers and the pamphlet described stories of massacres in graphic detail, which created a strongly negative public opinion against the Ottomans and in favor of the Christian Slavs. The “horrors” came to be known as “Turkish atrocities”. Martina Baleva’s (2012: 282) analysis of the painted images of the massacres published by the British newspapers demonstrates how the press mostly ignored the atrocities committed against Muslims and almost exclusively promoted the sufferings of Christian Slavs. While British press was relatively restrained in publishing explicit graphic imagery compared to French and Russian press, Baleva (2012: 281) observes that hand drawn illustrations of the massacres consciously invoked famous Western Christian iconography thus making these images accessible to Western public and creating a religious feeling at the same time. Gladstone employed a particularly racist language which described the Turks as “the one great anti-human specimens of humanity” and the image of the Muslim Turks as the oppressor of the poor Christians prevailed in British public, after which turcophilism was restricted to a few conservatives ( Baleva, 2012: 364). Chase Gummer (2010: 11–12) observes that the fury against the Ottoman atrocities were notably different than those directed against the ones committed by Russia for instance; the language and logic of humanitarian intervention had developed around this time but the phrase “humanity” was a “codeword” for domestic support and pressure in favor of Christian orthodox population, and European powers considered armed intervention only against the Ottoman Empire, not Russia or other oppressors. This revealed a racially and religiously charged identity issue.

Necmettin Alkan’s (2016) analysis of political cartoons in the European press between 1876 and 1909 demonstrates the most prevalent visual imagery the Ottoman Empire was associated with in European public. Working with an extensive sample of caricatures from each of the 34 years in between, Alkan (2016: 364) observes that the most popular image the Empire and Ottoman sultan was associated with was an “old man” or a “sick man” who is invariably drawn with a hookah or a tobacco. Occasionally and particularly during Ottoman suppression of insurgencies, the image of the old man was replaced with angry bully or a barbarian with a machete or scimitar in his bloody hands. Alkan (2016: 366) thus higlights the dual image of the Ottomans as either a bloodthirsty barbarian or a sick man in alternating contexts. Other major themes in the cartoons were the mockery of Ottoman reform process and the “hedonism” and “voluptuousness” of the Ottomans complemented with images of tobacco, harem, slaves and concubines, which, Alkan (2016: 367–368) observes, were parallel with the dominant image in classical orientalist texts.

Chase Gummer (2010: 155), in his impressive and extensive study of Turcophilism in German press from 1870 to the WW I, argues that although British public had its own share of Turcophile apologists during the events of 1876, German press was comparatively more balanced in its presentation of the turmoils in the Balkans. Despite Bismarck’s wishes, there was a considerable part of the German press that condemned the Ottoman Empire; “Muslim fanaticism”, which implicated all Muslims in the events, was the code word for Muslim deeds both in German and French press and some newspapers went so far as calling for the expulsion of the Turks form Europe ( Gummer, 2010: 157). Gummer (2010: 160, 164) also observes how the discussions about Turks and orthodox minorities drew on the domestic culture wars going on in Germany between Catholics and the Protestants; while Turcophile editors associated Catholic fanaticism with those of the “backward” orthodox Christianity under Ottoman rule, others conflated Muslim and Catholic fanaticism, comparing Muslim *softas* (madrasa graduates) with the Jesuits. The choice between Russians and Ottomans was a constant debate in Germany as well, and once the war was triggered coverage of both Russian and Ottoman atrocities was a priority for many German newspapers ( Gummer, 2010: 169, 175). Although Ottoman forces were defeated, their fierce resistance to Russian forces created an administration in Germany for the fighting power and discipline of the Turks among German officials and soldiers ( Gummer, 2010: 184).

Gürpınar (2012: 365) states that the reign of Abdulhamid II, who distrusted and diplomatically alienated the British further worsened the image of the Empire in British public and made it increasingly difficult to ignore the problems of the Empire; Ottomans epitomized “barbarism and bigotry” and they were “enemies of civilization”. Gummer (2010: 190) notes that the wave of violence against Armenians in the 1890s turned the British public overwhelmingly against the Ottoman Empire and even Tories dropped defending the Ottoman cause. French press on the other hand, was censured in order to protect the interests of French investors in the Empire, and for two years nothing was published on the massacres of Armenians ( Gummer, 2010: 225–26). There was a philarmenian movement in Germany as well but it was smothered by Turcophilism which:

was not just a reaction to the Armenian question, but part of broader concern for stability and security. Turcophile interpretations of the massacres tapped into a familiar repertoire of clichés from the German culture wars that had been used during the Great Eastern Crisis, as well as ways of viewing social order that privileged state violence ( Gummer, 2010: 191).

Many German newspapers were aware of the significance of public opinion for the Ottoman minorities and they claimed Armenian guerillas desired to raise European public opinion by inciting violence and hence inviting intervention ( Gummer, 2010: 208–209).

Ottoman decisive victory against the Greeks in 1896 after the Cretan separatist revolt, further reinforced the positive image of the Empire as a source of stability and order, and Greeks as weak and unfit to rule (ibid: 231). Particularly, a series of articles by German military advisor Marshall Colmar von der Goltz, appealed to the German conservatives through concepts of *Ordnung* and *Gehorsam* which he used to describe the Turks; one could recognize “the born dignity of a ruling people, instead of an impotent will” of the Greeks, “Turks were protecting ancient traditions of property and order against a chaotic onslaught from below” ( Gummer, 2010: 235).

A major event around the time was Kaiser William’s travel to Istanbul and Jerusalem with the intention of both closing the deal on Ottoman railroad constructions and demonstrating German solidarity with the Empire ( Gummer, 2010: 239). Gummer (2010: 242) writes:

William’s encounters with Islam provided a striking counterpoint to his disappointment with local “Oriental” Christian communities. Privately he was repulsed by the “fetishistic adoration” of the Eastern Rite Christians, and found the asceticism and discipline of Islam appealing after visiting the Al-Aqsa mosque and the Dome of the Rock. Writing to his cousin, Czar Nicohols II, the Kaiser mused that he would have become Muslim had he entered Jerusalem with no religion.

A German intellectual Friedrich Naumann “popularized the intellectual association between Islam and Protestantism in a collection of his travel writings, agreeing with the sentiment widespread among the Kaiser’s entourage that…Islam had more in common with Luther’s church” ( Gummer, 2010: 245).

This heightened sympathy and positive perceptions of the Ottoman Empire, however, was seriously shaken first with the Young Turk revolution in 1908 and soon after with embarrassing Ottoman defeat in the Balkan Wars in 1912–13 ( Gummer, 2010: 248). The Committee of Union and Progress once again turned the diplomatic face of the Empire to Britain and France, shunning Germany which had vehemently supported Abdulhamid. While a part of the press still continued Turcophile agenda, particularly after the defeat of Ottomans against Italians and soon after the Balkan alliance, the positive image was drastically shattered; complaints about Turkish officers lacking proper mental and physical discipline emerged and the accusation of “fatalism” resurfaced ( Gummer, 2010: 283:84). Gummer (2010: 300–304) concludes that German Turcophilism which had much to do with domestic struggles within Germany (mainly protestants vs catholics), still survived and even thrived during the WW I and affinities between Germandom and Islam was reiterated.

Konrad (2011: 34) summarizes that overall in the nineteenth century:

…numerous stereotypes circulated that to a degree continue to shape the image of Islam and Muslims to this day. It was alleged that Islam does not know a separation of state and religion, that Muslims cannot conceive of a secular social and state order, that knowledge stagnates in Muslim societies and can only be developed through the adoption of European ideas and standards, that Islam oppresses women and that Islam is anti-modern. Particularly widespread was the stereotype that Islam is the obstacle to modernization, enlightenment and progress in the Muslim Orient, and that Islam is the reason why it was inferior in political, military, economic and, ultimately, cultural terms.

While, as the literature suggests, the picture of European perceptions of the Ottoman Empire and the Turk is undeniably more complex than simply negative images, it is also true that with the exception of Germany, the image of the Empire was mostly and in the final analysis a negative one which was underscored by racial, religious and ethnic essentialism and the dominant discourse of Orientalism which was a product of the imperial power relations between Europe and the “Orient.” Moreover, the competing identity narratives regarding the Empire in individual European countries were determined by competing claims about respective national identities. Hence, while Britain and France emphasized civilization and progress as yard sticks in judging the worth of the Turk, German public was looking for a strong rule, order, discipline and dynamism, which reflects on construction of European national identities in the mirror of the Turkish other. Comparisons of the Turk with the Russians, Greeks and minorities within the Ottoman Empire appears to be a significant element in delineation of the representations of the Turk, which was driven by different trends like philhellenism, Russophobia, and philarmenia.

A major lacuna in the literature seems to be the lack of reflection on the level of analysis (i.e. between public and elite representations, policy makers vs scholars/intellectuals, cultural drivers vs political drivers) and interaction between different levels which makes the identification of the sources and causes of the conflicting narratives regarding the Empire difficult. Another problem with few exceptions is the fragmentary nature of the scholarship which focus mostly on individual moments and impact of particular political events and does not deal with the transformation of representations in the long duree (see Gummer, 2010; Gürpınar, 2012).

### Ottoman representations of Europe

The eighteenth century marks the beginning of “Westernization” attempts in the Empire, that is the beginning of Ottoman curiosity regarding the causes of Western military and technological superiority. After the Treaty of Karlowitz (1699) which brought territorial losses to the Ottoman Empire in Europe, the sense of Ottoman superiority was seriously shaken, and Ottoman bureaucrats began to accept that they could learn from Europe. This manifested itself in the gradually increasing number, and spatial variety of ambassadorial missions to European capitals throughout the eighteenth and early nineteenth century until permanent embassies were established in 1835 (see Unat 1968). These missions were the main point of interaction and learning between the Empire and Europe, and the observations and opinions of Ottoman ambassadors are practically the only source on Ottoman perceptions on Europe until Tanzimat era when slowly newspaper printing helped produce a variety of sources (see Göçek, 1987).

In his book on how Ottoman concept of Europe has changed from the eighteenth century to the Turkish republic and how this concept revealed Ottoman-Turkish representations of self and the European other, Einar Wigen (2010: 107–10) marks two significant changes in Ottoman representations of Europe. The first significant change happens around the late eighteenth century with the shift of naming from “Frengistan,” meaning the land of the Franks carrying strong religious connotations, to “Avrupa” (Europe), a more secular definition of Europe ( Wigen, 2010: 30–35). Concurrent with this shift, in proposing imitation of Europe as the only way to secure the future of the empire, “Ottoman reformists split European practices semantically into a 'material' and a 'spiritual' aspect, making it possible to imitate the former while still rejecting the latter” ( Wigen, 2010: 30). Marinos Sariyannis (2015: 175–80) identifies two attitudes within Ottoman political writing of the time: traditionalists and Westernizers, recognizing, however, that both attitudes had a similar approach to Europe. In bureaucratic writing Europe is associated with order whereas the Ottoman self is associated with stagnancy and ignorance ( Wigen, 2010: 38). Fatih Yeşil also notes how a major Ottoman ambassador in 1890s, Ebubekir Ratib Efendi associates European orderliness and discipline with classical Ottoman ideal of order and circle of justice ( Yeşil, 2007). İbrahim Şirin, on the other hand, notes how European cities were associated with sexual practices which were either intriguing or detestable depending on the position of the bureaucratic authors ( Şirin, 2009).

With the Gülhane Edict of 1839, relations of Ottoman Empire with Europe entered a new phase. The edict aimed to reorder the state society relations in the Empire while holding the European states witnesses to its pledge to restore and implement justice and prosperity. The proclamation of Tanzimat, hence, was closely linked to the focal issues of status in international society and state-society relations. Particularly, Sadık Rıfat Paşa is accorded much attention in the literature ( Aydın, 2007; Baykara, 2007; Mardin, 2000; Wigen, 2010). Sadık Rıfat Paşa was influential in drafting of the Gülhane edict, was an envoy to Vienna, and he also coined the term “civilization” ( *medeniyet*) for the first time in Ottoman language. Rıfat Paşa used civilization to mean the new European order which rested on principles and peace and prosperity and associated it, like Ebubekir Ratib Efendi, with the Ottoman ideal of Circle of Justice ( Aydın, 2007: 18–20; Mardin, 2000: 180; Wigen, 2010: 48–9). Mardin (2000: 170–80) also observes how Sadık Rıfat Paşa preferred Metternich’s approach to reform, which emphasized order, efficiency, stability and prosperity, as opposed to Mustafa Reşid Paşa who had better relations with British and emphasized abstract liberty. Wigen also notes the work of Mustafa Sami Efendi on Europe which emphasized knowledge and learning as the primary cause of European success ( Wigen, 2010: 51). Şirin (2009), on the other hand, observes how there was still resistance to European influence and intervention among Ottoman higher bureaucracy and how Ottoman diplomats were actually divided into roughly two factions whose political interests determined their perception of Europe. Niyazi Karaca (2004) also demonstrates in his analysis of the memoirs of Mehmet Akif Paşa, the first Ottoman foreign secretary, how Akif Paşa perceived a double standard in application of international law particularly on the part of the British during the infamous Churchill incident in 1836. Although there are very few records of public perceptions of Europe at the time, literature notes that the destructive consequences of Mahmud II’s Westernizing reforms helped create an image of him as the “infidel sultan”.

The desire of the Ottoman palace to present itself as part of the European civilization was clearly seen in symbolic events as well, such as the visit of Ottoman Sultan Abdulaziz to the Paris World Fair of 1867 and the display of Ottoman culture as part of the Fair ( Zaptçıoğlu, 2012).

The Young Ottomans, a small cadre of intellectuals which emerged from the mid rank bureaucracy in the 1860s, came up with extensive criticism of Tanzimat reforms and Westernizing policies and oppose the arbitrary rule of the Ottoman Sublime Porte. In the writings of intellectuals such as Namık Kemal and Ziya Paşa, Europeean other was represented as “belonging to a greater community with an inclusive identity, one that also the Ottoman Empire can take part in if it adheres to the same practices” ( Wigen, 2010: 57). These authors represented civilization not “as something inherently European, but as something the Europeans have succeeded in achieving” (ibid). Moreover, while the European other was associated with rationality and morality, Ottoman self is associated with irrationality ( Wigen, 2010: 60–61).

Young Ottomans also came up with the first comprehensive Ottoman liberal ideology and they promote a form of constitutional monarchy, which they learned from European authors such as Rousseau and Voltaire and equated with the ancient Islamic principle of consultation ( *meşveret*) ( Mardin, 2000). In Young Ottoman ideology, modern European ideals such as equality and liberty represented the “highest Islamic ideals” which the Ottomans had deviated from. Nazan Çiçek (2010), on the other hand, deals with another aspect of Young Ottoman opposition. Situating their reaction to Europe within the context of the Eastern Question and particularly the Cretan insurrection of 1868–69, Çiçek demonstrates how Young Ottomans contested European intervention into the domestic affairs of the Empire particularly regarding the affairs of Christian minorities. Hence, while they desired to become part of the European international civilization, they also wanted to protect the integrity of the Ottoman identity as an inclusive project covering minorities. Finally, Wigen also notes alternative views on Europe and civilization, such as Ahmet Cevdet Paşa, a conservative minister of justice and compiler of the first Ottoman civil code *mecelle* ( Wigen, 2010: 61–62). Cevdet Paşa uses civilization in the plural to indicate and Islamic civilization and rejects European laws and mores as unfitting of the Ottoman way; in his view Islam represents the core of Ottoman identity, the *Volksgeist*.

A major issue which arose in the late Tanzimat era is the reaction against over-Westernization, that is the criticism of prevalent Western oriented consumption and leisure patterns in the urban centers of the Empire ( Mardin, 1974; Wigen, 2010: 64). The concept that symbolized this reaction was *alafranga* (francophile) which was contrasted with *Alla Turca.* Many late nineteenth century authors have worked with the trope of the “fob” or the “dandy” who did not see the real core of European superiority and simply sufficed with imitating European fashion blindly. Mardin (1974) brilliantly analyzes the dynamics behind this reaction and concludes that the trope of *alafranga* served the intellectual mobilizers such as the Young Ottomans because it served their concerns regarding the Ottoman identity, and it also resonated with the public because these “deviant” consumption patterns went against the grain of Ottoman traditional democratic patterns of consumption and leisure activities. As such, while the Ottoman self was linked with the necessary aspects of Westernization, *alafranga* was associated with the “unnecessary adoption of European ways” and this dichotomy was reflected on the urban scape of Istanbul whereby certain districts of Istanbul (e.g. Beyoğlu) was associated with moral degradation ( Wigen, 2010: 65).

In an interesting study, Avner Wishnitzer, sheds new light on the *alla turca-alafranga* binary, that of timekeeping ( Wishnitzer, 2015). Wishnitzer analyzes how a particularly Turkish way of time-keeping (clocks would be reset every day at sunset at variance with seasons), the *alla turca* clock developed from the eighteenth century onwards parallel to Ottoman modernization process and how later in the nineteenth century with increases relations with Europe, Turkish time-keeping practices started to coexist with *alafranga* one, whereby many people would have two sets of time standards. Wishnitzer (2015: 152) ties his analysis to the concept of progress and the notion that the Ottomans lagged behind, where inadequately implemented or observed temporal practices became metaphors for their standing in the “race between nations”.

The Hamidian Era which lasted 32 years between 1876 and 1908 is generally marked with the rise of an anti-Westernist Islamism as an ideology both as part of Abdulhamid’s deliberate policies to increase the legitimacy of his rule and as part of a broader trend in the Middle East ( Aydın, 2007; Deringil, 1991). Abdulhamid II strove to perfect his image as a Muslim leader, caliph and protector of Muslims and a general leader of the Muslims all over the world ( Deringil, 1991). In addition to this, he spent conscious and careful effort to present the Ottoman Empire and its people in a better light as part of humanity in the eyes of European public ( Baleva, 2012; Greene). For instance during the Russo-Ottoman war of 1877–78, in response to the European public outcry against “Turkish atrocities”, Abdulhamid arranged for photos of wounded Muslims to be taken and thus had the “Russian atrocities” documented ( Baleva, 2012). In these photos, which were dispatched the European embassies, special care was taken to portray Muslim sufferers individually and humanize them. In a much broader effort, Abdulhamid had an extensive album of photographs prepared with photos of social life from all around the Empire and sent those to various libraries in Europe and America (Greene). These albums particularly aimed at battling the eroticized and exoticized image of the Ottoman Empire prevalent at the time in Western public; they portrayed Muslim subjects as ordinary people (Greene).

An interesting development during the Hamidian era is the internalization and reproduction of the orientalist discourse within the Empire in a way that reflects on the eastern periphery of the Ottoman realms ( Deringil, 2003; Makdisi, 2002). Based on his observations on Ottoman government in Mount Lebanon, Makdisi argues that in the second half of the nineteenth century Ottoman government embraced the Western orientalism’s “underlying logic of time and progress” and gradually established a regime “in which an advanced imperial center reformed and disciplined backward peripheries of a multi-ethnic and multi-religious empire” ( Makdisi, 2002: 769). Makdisi (2002: 779) shows that “as part of this project of imperial nationalism that placed the Empire on a par with other ‘civilized’ states, Ottoman modernization generated its discursive opposite, the pre-modern within the empire, whether in the Danubian principalities, the sands of Arabia, the cities of Syria, or Mount Lebanon”. This discursive othering particularly targeted Druzes, Marnoites, Yezidis and other similar groups standing low in the Ottoman ethnic and religious social hierarchy. In a parallel argument, Deringil (2003: 311) deals with the “civilizing mission” mentality of the late Ottoman government by which it “came to conceive of its periphery as a colonial setting”. In a fashion which could “pretty much be summed up as the White Man’s Burden wearing a *fez*” an Ottoman bureaucrat described the tribes living to the south of Ottoman Libya as “savages and heretics” to be saved only “by an invitation into the True Faith” ( Deringil, 2003: 312). This colonizing governmentality, however, was not simply invented anew in the nineteenth century; Deringil demonstrates how Ottomans dipped “into a whole grab bag of concepts, method and tools of statecraft, prejudices, and practices that had been filtered down the ages,” invoking the classical Ottoman distinction between nomadism and civil life ( Deringil, 2003: 312, 317), in a way that was parallel to the one observed by Ilıcak (2011). This attitude revealed an Ottoman national identity formation process, which emphasized modernization and civilization and by which the peripheral subjects were gradually less Ottoman ( Makdisi, 2002). Again, Ottoman orientalist approach to its subjects demonstrates how issues of civilization, state-society relations and nation formation were inextricably linked in the discursive realm.

explaining the global emergence of an anti-Westernist discourse by the late nineteenth century, Cemil Aydın compares the emergence and trajectory of anti-Western ideas in Japanese and Ottoman cultures and respective ideologies of pan-Asianism and pan-Islamism ( Aydın, 2007). He rejects the essentialist explanations which pit West and the rest against each other and he also objects to reducing anti-Westernizm to a simple reaction to Western colonial and imperialist policies; instead he argues that pan-Islamist visions of world order, just like pan-Asianism, was a reaction to the “legitimacy crisis of a single, globalized, international system” which had been intellectually justified through Orientalist and racist discourses ( Aydın, 2007: 4–7). Hamidian era was particularly fruitful regarding rebuttals of Orientalist arguments and the argument that European civilization had been built upon the achievements of Arabs were put forward ( Wigen, 2010: 70). Particularly, a famous and authoritative Orientalist, Ernest Renan’s presentation of the Muslim World and its past as the history of an inferior Semitic race which was incompatible with science and progress triggered a massive reaction from Arabic and Turkish speaking Muslim intellectuals in the Empire ( Aydın, 2007: 47–53).

A significant event in the first years of the twentieth century was the Japanese victory over the Russians in 1905, which was a watershed event for the way international order and Orient-Occident relations were imagined ( Aydın, 2007). Japanese victory symbolized the potential for success and modernization of an oriental culture and hence inspired confidence in many colonial contexts as well as the Ottoman Empire. Wheras Japan had already been an object of interest among intellectuals and image of the Empire as “Japan of the Near East” had been used to contest the image of “Sick man of Europe” (see Worringer, 2004), with the victory, Japan began to symbolize an ideal model of a nation which could compete successfully with Western dominance. Worringer demonstrates how certain features of Japanese society such as reverence for ancestors, bushido ethos and samurai spirit resonated with Ottomans.

Concurrent with the rise of Islamism, we see the emergence of Turkism as an alternative discourse on national identity. Umut Uzer’s comprehensive study on the intellectual history of Turkish nationalism provides three factors that contributed to the emergence of Turkism: the ethnic separatist movements of minorities in the Empire such as Bulgars, Armenians, Arabs and Albanians which gradually made an inclusive Ottoman identity less and less realistic, emergence of turcology and studies of several eminent Turcologists on pre-Ottoman Turkish history and culture which appealed to Ottoman intellectuals and finally the influence of several Russian born Turkic intellectuals such as Yusuf Akçura and İsmail Gasprinski who were awakened to national consciousness in response to Russia’s pan-Slavic policies ( Uzer, 2016: 2–3, 16–17). Works of turcologists such as Arthur Lumley Davids, Constantin Borzecki, Leon Cahun, Friedric ax Müller and particularly the recovery of ancient Orhon Turkic inscriptions by Wilhelm Tomsen helped instill national feelings among Turkish literati ( Uzer, 2016: 18–19).

For the period following the Young Turk revolution of 1908 and the promulgation of the second constitutional period, Einar Wigen (2010: 75) notes the most significant change as the replacement of Ottoman and Muslim with Turk and Turkey. A major debate emerged during the period with Yusuf Akçura’s (1876–1935) famous article *Three Types of Policy,* which categorized the choices available to the Ottoman Empire as Ottomanism, Islamism and Turkism, three selves from which the Ottomans had to choose. Akçura believed Turkism was the most realistic choice for the Empire. To this Ziya Gökalp (1776–1924), the ideological father of Turkish nationalism, replied with another article which suggested a blend of three paths; the real Turkish nation would be a result of modernization, Islamization and Turkification ( Uzer, 2016: 55–80; Wigen, 2010: 75–79).

The literature pays particular attention to the ideas of Ziya Gökalp, and his usage of concepts of civilization and culture ( Davison, 2006; Heyd, 1950; Karadaş, 2010; Topal, 2017; Uzer, 2016; Wigen, 2010). As demonstrated above the concept of civilization had been a key concept in modernization and Westernization debates in the Ottoman Empire throughout the nineteenth century. Gökalp, in addition to civilization, coined the concept of culture ( *hars*), inspired from the German concept of *Kultur*, to define the national community. Gökalp associated civilization with universal, international, technical, economic and progressive categories, and culture with particular, national, religious and immutable categories ( Topal, 2017: 13). associating Europe with material and technological qualities, Gökalp approves westernization in these areas, whereas by using the concept of a national community, he draws a line between the West and the Turkish national community ( Karadaş, 2010). Heyd (1950: 63–4, 164–66) concludes that Gökalps nationalism is a kind of linguistic nationalism quite like German romantic nationalism and Davison (2006) argues that behind binary concepts of culture and civilization lies a quest for cultural authenticity frequently observed in postcolonial thought. Using Dipesh Chakrabarty’s work he demonstrates how Gökalp provincializes Europe through his dual concepts. Topal also agrees that Gökalp’s nationalism is a romantic and revivalist nationalism, however he also shows how Gökalp defines these originally European concepts with reference to historical and social dichotomies in Ottoman past such as folk Islam vs elite Islam, folk literature vs court literature, religious fraternities (Sufi brotherhoods) vs religious establishment; former categories associated with dynamism and authenticity, latter categories associated with corruption, decay and imitation ( Topal, 2017). Hence, in defining Turkishness Gökalp others not only Europe but also the Ottoman past. Topal also argues that Gökalp’s conceptualization influenced many conservative thinkers in the twentieth century.

Şükrü Hanioğlu’s (1997) article on the Westerners ( *Garbçılar*), a group of heavily pro-Western intellectuals, demonstrate their confrontation with Islam and infatuation with Western sciences and technology. Led by Abdullah Cevdet, Westerners imagined a society based on science and technology, they criticized traditional Islam and advocated clearing the religion of all dogma and superstition, and they were enamored with the idea of progress. Still they would embrace Islam, as they saw it essential to Turkishness and they criticized Western imperialism and the emphasis on Christianity in European identity ( Hanioğlu, 1997: 145).

Two other sources deserve mention here. The first is Palmira Brummet’s general survey of Ottoman post-revolutionary press (2000). In her book Brummet deals extensively with issues of ideology, order, critique of imperialism, competing representations of European nations, fashion, the problem of *alafranga,* technology and education all in thematic chapters thus giving a broad picture of Ottoman self and other dichotomies. Second, in his work on propaganda efforts of the Committee of Union and Progress, Erol Köroğlu (2007) deals with the Ottoman literature during the First World War and argues that this period was culturally significant in the formation of Turkish identity. Using the tools of literary criticism, Köroğlu shows how the war years witnessed a constant negotiation and compromise between the three competing narratives of national identity cited above. Although Ottoman propaganda effort was weak compared to European nations, Köroğlu concludes that the war years were formative for the Turkish national identity.

Overall, in the literature on there is an emphasis on bureaucratic representations of Europe which is understandable considering the lack of source material over which public representations could be analyzed and further elite and public representations could be contrasted. Only Şerif Mardin deals with the analysis of the reaction against over-Westernization in the intersection of elite and common sensibilities. Like the literature on European representations, there is a considerable number of studies on individual moments and short periods and scant research on diachronic change of Ottoman identity. While Wigen (2010) and Aydın’s (2007) studies provide a general overview of the transformation of Ottoman identity vis-à-vis the West/Europe, Aydın’s work focuses on anti-Westernism and pan-Islamism, and Wigen’s research (an MA thesis) is based on a limited number of primary and secondary sources. There is an obvious need for further research to test and complement their arguments in a more systematic way with methodological tools of discourse analysis and identity theories.

One final lacuna in the literature appears to be the lack of research on the diversification of Ottoman representations of Europe vis-a-vis different nations. While it is noted by Wigen (2010) that Ottomans increasingly distinguished between major European nations, England, France and Germany in the late nineteenth and early twentieth century, there is little research on how this reflected Ottoman self-representations, particularly considering the intensification of German-Ottoman relations and Ottoman adoption of the German *Kultur* argument especially in the writings of major intellectuals like Zia Gökalp. 

### The Focal Issues

***Civilization.*** Of the four focal issues of our work package civilization seems to be the most relevant issue for the duration of the nineteenth century by virtue of being an umbrella concept which covers the other three issues as well. It is also the most widely running theme in the literature.

Civilization was a central concept for European identity as well as a key word in legitimizing imperial and colonial projects of European nations (See Bowden, 2009). Accordingly, many cultures under direct and indirect Euroepan rule had to face the concept of civilization and make sense of it in their own way (See Pernau *et al.*, 2015). Ottoman Empire which was the next-door neighbor of Europe together with Russia was one of the earliest cultures to grapple with the semantics of Western superiority. Already in the late eighteenth and early nineteenth century image of the Ottoman Empire was mostly associated with concepts such as barbarism, despotism, stagnation and backwardness, all of which reflected the self-perception of Europe as civilized and progressive ( Çırakman, 2001; Konrad, 2011; Yapp, 1992). In response Ottoman bureaucrats started using the concept as early as 1830s in their increasing diplomatic relations with European nations ( Baykara, 2007).

While for Europe the concept of civilization and the dichotomous representation of the Orient as uncivilized remained more or less constant throughout the nineteenth century with the particular qualities associated changing over time, in the Ottoman Empire civilization seems to have varied both synchronically and diachronically ( Baykara, 2007; Wigen, 2010). Einar Wigen explores in detail various concepts attributed to the word ‘civilization’ in Ottoman context, some of which are ‘education of Ottoman man and the practice of orderliness’, ‘European international system’, ‘settled, urban and sedentary life’, ‘the level of ideational and material development achieved by Europe’, ‘attaining of refinement’ and ‘bourgeoisie sociability’(Wigen, 2015). Hence, while initially civilization is synonymous with international order and thus closely related to the focal issue of status in international society, later in the nineteenth century it is additionally associated with broader social practices and patterns all of which bear significantly upon the issue of proper citizenship and state-society relations. Again, as Wigen notes while initially civilization was a concept in the singular for Ottomans, by the last few decades of the nineteenth century alternative civilizations could be imagined and the term Islamic civilization had been coined ( Wigen, 2010). This was parallel with the rise of anti-Westernism and emergence of alternative international orders such as pan-Islamism and pan-Asianism ( Aydın, 2007).

The concept of civility was also significant in that it represented an overlap of Ottoman self-representations inherited from classical doctrines and internalization of Western orientalist representations. As highlighted by Ilıcak (2011), Makdisi (2002) and Deringil (2003), Ottoman self-representation as civil as opposed to nomadic and savage followed a traceable pattern during the nineteenth century by which Ottoman government imagined its own center-periphery relations.

By the second constitutional period civilization was an issue whose definition had become a distinguishing factor between the competing Ottoman ideologies of Islamism, Turkism and Westernism. It was particularly a defining concept in Ziya Gökalp’s nationalism and his distinction between civilization and culture was the most successful in defining limits and boundaries of emerging Turkish nationhood ( Davison, 2006; Karadaş, 2010; Topal, 2017; Uzer, 2016; Wigen, 2010).

***Status in International Society***. Early in the nineteenth century, it was of paramount importance for the Ottoman Empire to be a part of the European international order. After the Greek Independence which was secured by an alliance of the British, the French and the Russians and facing a domestic challenge from Egypt, the Ottoman government increasingly turned to European states and strove to improve its diplomatic relations to benefit from the advantages of international law. As noted above, the first usage of the concept of civilization in Ottoman language was to denote the post-Napoleonic European order based on balance and peace to which a part of Ottoman bureaucracy aspired ( Aydın, 2007; Mardin, 2000). Hence, the Tanzimat Edict (1839) invoked European nations as witnesses to Ottoman domestic reform and the Reform Edict of 1856 coincided with the Paris Conference which would end the Crimean War in which Ottomans allied with the British and the French against the Russians. However, with the Cretan insurrection in 1866 and Bulgarian revolt in the 1876, Ottoman status in international order was again compromised due to treatment of minorities which is closely linked to the focal issue of state-society relations. Status of Ottoman Christian subjects was a constant issue in Europe ( Gummer, 2010; Gürpınar, 2012) and the constant intervention of European states and Russia in favor of Ottoman subjects impaired Ottoman sovereignty and her trust in the international system which reflected on the representation of Europe ( Çiçek, 2010).

In the final decades of the nineteenth century alternative projects of international order emerged, such as pan-Islamism and pan-Asianism as a response to the failure of the project of a single global order lead by Europe and its justification mechanisms which drew heavily on racial explanations ( Aydın, 2007). These alternative projects also came with a serious anti-Westernist discourse. Pan-Islamism and the cause of the caliphate was the Ottoman manifestation of this trend.

While pan-Turkism was not as anti-Westernist as pan-Islamism, the adoption of German *Kultur* argument against civilization during the emergence of Turkish nationalism particularly in the writings of Ziya Gökalp, signifies another significant shift in the way Ottoman Empire imagined and represented the international order.

***Nationalism***. The emergence of nationalism has attracted a lot of attention in modern Ottoman studies with a particular focus on the post-1908 period. During the Tanzimat era, the main solution to the Ottoman problem of citizenship was the idea of Ottomanism, an inclusive identity which proposed the allegiance to Ottoman throne as a unifying factor for all Ottoman subjects ( Mardin, 2000; Uzer, 2016). As argued by Umut Uzer (2016), fate of Ottomanism was sealed with successive nationalist revolts in the Empire and inspired by the research on ancient Turkic history several intellectuals began to envision a less inclusive Turkish nationalism. While competing with alternative identity projects like Islamism, Turkism still shared a lot of common ground with these alternative projects such as definition of the nation by distinguishing it from the international civilization which was associated with Western civilization ( Topal, 2017).

Literature emphasizes that Turkism in the late Ottoman Empire was not based on racial premises; it was rather a linguistic or cultural nationalism with an emphasis on Islam as an essential component of Turkishness ( Heyd, 1950; Topal, 2017; Uzer, 2016). Topal (2017) also argues that Turkists like Ziya Gökalp actually promoted a competing definition of Islam which emphasized spirituality, experience and a public morality instead of ritual practice, legality and individual morality. The relationship of Turkists with the ruling Committee of Union and Progress (CUP) has also been discussed: Uzer (2016: 30–31) concludes that while the CUP at times used Turkist sentiments pragmatically and tried to control the Turkist organizations, Turkists maintained a careful distance to the government and this mutual distance persisted into the early republic. Finally, the work of Köroğlu (2007) highlights the propaganda efforts of the CUP during the WWI, which contributed to the rise of nationalist sentiments.

***State-Citizen Relations***. The relationship of Ottoman state with its non-Muslim subjects was a major concern for European states throughout the nineteenth century. Starting with the Greek revolt and their subsequent independence, European powers frequently intervened in the domestic affairs of the Ottoman Empire in order to secure the rights of its non-Muslim subjects against the perceived “despotism” of the Ottoman government. Russia invoked its status as the protector of orthodox Christians to incite agitation in the Balkans and create public opinion against the Ottoman Empire in Europe. Two major reform projects, the Gülhane Edict of 1839 and Reform Edict of 1856 were both attempts by the Empire to reorganize the state society relations, the former adjusting taxation and the newly introduced conscription and the latter by introducing legal equality to all Ottoman subjects regardless of religious denomination. However, the Ottoman government was not able to solve the status and material inequalities between the subjects in practice and with the growing unrest in the Balkans from 1876 onwards, the Empire’s image gradually deteriorated and began to be associated with “atrocities” ( Baleva, 2012; Gummer, 2010; Gürpınar, 2012).

As noted, the relations between the modernizing Ottoman government and its subjects –particularly non-Muslim and heterodox groups- in the periphery took on a colonial twist. Ottoman government took it upon itself to civilize, educate and modernize these groups in an effort to reach an inclusive identity under the banner of Ottomanism and loyalty to the throne ( Deringil, 2003; Makdisi, 2002; Mardin, 2000). As noted above this situation created a vicious circle of minorities problems for the Ottoman government whereby separatist groups within the ethnic and religious minorities would incite unrest citing Ottoman maladministration hoping for foreign intervention, after which the Ottoman military would suppress the revolts (generally in a bloody fashion) thus fulfilling the prophecy ( Gummer, 2010). Successive breaks of different minority groups from the Empire meant the failure of Ottomanism, and gradually led to narrower definitions of national identity. Particularly after the Balkan Wars and massive Muslim population flow into the Empire, the national identity was associated more and more with Muslimhood and Turkish ethnicity as evidenced by Ziya Gökalp’s project, in which non-Muslims did not have a place ( Davison, 2006; Heyd, 1950; Topal, 2017; Uzer, 2016). 

## The second period (1923–1945)

The second period (1923–1945) under scrutiny, namely the Republican period, starts from the proclamation of the Turkish Republic and lasts until the end of the Second World War. The Republican period is marked by key political and cultural developments that completely changed the governing mechanism of the country and triggered a remarkable societal transformation. Crucial political events during this period include the signature of the Treaty of Lausanne (24 July 1923) confirming the independence and equal sovereignty of the new Turkish state, the compulsory population exchange between Greece and Turkey (1923–1924), the abolition of the Caliphate (3 March 1924), the decision of the League of Nations to confer the sovereignty of Mosul to Iraq (5 June 1926), The law conferring women in Turkey the right to vote and get elected in national elections (5 December 1934), The Montreux International Straits Convention (20 July 1936) authorizing Turkey’s sovereignty over the Istanbul and Gallipoli staits, the accession of the Sanjak of Alexandretta (Hatay) to Turkey (30 June 1939) and the ‘Capital levy’ issued to non-Muslim citizens of Turkey during the Second World War (11 November 1941).

Notable cultural developments contributing to the transformation of the Turkish society include the adoption of the Hat reform (25 November 1925), the introduction of the Latin alphabet (1 November 1928), the victory of Keriman Halis winning the title of Miss World in Belgium (1932), the immigration of Nazi-persecuted Jewish German and Austrian intellectuals to Turkey triggering the University reform in the Republic (1933), the 12th International Woman Suffrage Alliance Congress held in Istanbul (18–25 April 1935) and the World Olympics held in Berlin (1–16 August 1936) where Turkey won its first ever medals.

Moreover, there were a number of official and unoffical visits by notable European figures to Turkey and Turkish visits to Europe including the visit by Greek Prime Minister Eleftherios Venizelos resulting in the signature of a treaty of friendship (25 October 1930), the visit of Richard N. Coudenhove-Kalergi, an Austrian-Japanese pioneer of European integration (early 1930s), the King of England, Edward VIII’s visit to Istanbul (4–6 September 1936), the participation of Turkish Prime Minister İsmet İnönü to the Coronation of King George VI and Queen Elizabeth in London (9–21 May 1937) and British Prime Minister Winston Churchill’s meeting with President İnönü in Adana during the Second World War (30 January 1943).

In comparison to other three periods, the identity representations of Turkey and Europe during the Republican era remain rather under-studied. Even though there are numerous sources touching upon the Republican period, there is an evident lack of in-depth analysis on the Republican era. Comprehensive scholarly volumes covering a long period of Turkish-European relations rather neglect this period ( Kumrular, 2008; Levin, 2011; Neumann, 1999; Soykut, 2003). Others only offer a concise look at the period focusing either on the historical background or current developments ( Ahıska, 2003; Brewin, 2000; Diez, 2004; Müftüler-Baç, 2000; Robins, 1996). Besides, there is no research dealing with the identity depictions of Turkey and Europe during the Second World War. The existing literature on the Second World War is either composed of sources with a general perspective on Turkish foreign policy ( Deringil, 1989; Oran, 2001) or works particularly focusing on the Capital levy ( Aktar, 1996; Aktar, 2000; Coşar, 2003; İnci, 2012). Moreover, most works dwelling on the Republican period rather examine Turkey’s top-down Westernization process without offering a detailed analysis on how Europe was perceived in the Republican Turkey and how the Republican transformation was received by European society ( Heper, 2004; Keyder, 1993; Keyman, 2006; Nas, 2001; Polat, 2006). The edited volume entitled “The image of the Turk in Europe from the declaration of the republic in 1923 to the 1990s” by Kuran-Burçoğlu (2000) hitherto remains the most comprehensive scholarly research on the European perceptions of Turks from 1923 onwards, but the book fails to live up to its promise of explaining in detail how the declaration of the Turkish Republic ‘as a turning point within the history of the image of Turks in Europe’ was observed by Europeans ( Kuran-Burçoğlu, 2000: 12). Nevertheless, several chapters of the book provide brief insights on how the Republican Turkey was viewed in different national (mostly Eastern European) settings ( Bibina, 2000; Murgeseu, 2000; Sabatos, 2000; Tahir & Türker, 2000). Especially, the chapter by Bibina (2000) exhaustively discusses the impressions of Vladimir Dimitrov, a famous Bulgarian painter during his visit to Istanbul in 1926.

There are only a handful of sources that specifically focus on the representation of Turks and/or Europeans during the Republican era, and among them, two recent studies stand out, namely “Turkey and the idea of a European Union during the inter-war years, 1923–39” by Barlas and Güvenç (2009), and After Defeat: How the East Learned to Live With the West” by Zarakol (2010). The work of Barlas and Güvenç proves to be an original attempt to scrutinize the European perceptions of Turkey during the Republican era through a detailed discussion of the endeavors for the establishment of an early version of European Union ^1^ and how Turkey’s inclusion in such a Union was contemplated by Europeans. Zarakol, on the other hand, focuses on the Turkish side of the story and succinctly explains how the Republican elite perceived Europe or West and made efforts to remedy the sense of defeatedness and build self-confidence through Westernization. However, inspite of these prominent scholarly works, there is still a lack of a comprehensive analysis focusing on both the Turkish depictions of Europeans and European perceptions on Turks during the period in a comparative fashion. Zarakol shares useful insights over the Turkish image in Europe giving examples from the British press, but the bulk of the work focuses on how the Republican Turks approached the West.

The extant scholarly literature during the Republican period is primarily informed by the disciplines of History ( Aktürk, 2007; Aytürk, 2004; Barlas & Güvenç, 2009; Criss, 2008; Hanioğlu, 1997; Lewis, 1961; Murgeseu, 2000; Tachau, 1964; Waterfield, 1973; Yapp, 1992) and Politics ( Brewin, 2000; Criss, 2008; Helvacıoğlu, 1996; Heper, 2004; Keyman, 2006; Kösebalaban, 2007; Müftüler-Baç, 2000; Nas, 2001; Oğuzlu, 2002; Polat, 2006; Robins, 1996). Nevertheless, there are also works informed by other genres such as Literary Studies ( Clark, 2013; Ergin, 2010; Sabatos, 2000; Tahir & Türker, 2000; Wood, 1929), Sociology ( Ahıska, 2003; Keyder, 1993; Zarakol, 2010), Fine Arts ( Bibina, 2000; Kuran-Burçoğlu, 2003), Architecture ( Bozdoğan, 1998), City Planning ( Tekeli, 1998), Music ( Turan & Komsuoğlu, 2007) and Sports ( Şenyuva & Tunç, 2015) which provide a multi-disciplinary value to the research on the Republican period. However, as previously highlighted, except for a handful of sources, the bulk of the literature does not necesarily focus on the identity discussions between Turkey and Europe on a systematic fashion, but briefly touches upon the effects of the Republican transformation in the creation of a new Turkish identity and its reflections in Europe.

In terms of methodology, discourse analysis focusing on political speeches with a particular focus on the speeches of Atatürk and other Republican elite such as Tevfik Rüştü Aras, Mahmut Esat Bozkurt and Hamdullah Suphi Tanrıöver, as well as the speeches and comments of European political figures including Aristide Briand and Edouard Herriot, stands as the dominant approach ( Barlas & Güvenç, 2009; Bora, 1997; Döşemeci, 2013; Lewis, 1961; Robins, 1996; Zarakol, 2010). Historical method with reference to archival research, including the archives on the minutes of the League of Nations sessions, and British, French, Italian and Turkish diplomatic archives, remains another popular method used in the literature ( Barlas & Güvenç, 2009; Criss, 2008; Lewis, 1961; Waterfield, 1973). Moreover, textual analysis examining history books ( Aktürk, 2007; Murgeseu, 2000), national press ( Barlas & Güvenç, 2009; Wood, 1929; Zarakol, 2010) and literary texts such as poems and novels ( Ahıska, 2003; Clark, 2013; Ergin, 2010; Sabatos, 2000; Tahir & Türker, 2000; Zarakol, 2010) proves to be another notable methodological preference. Finally, studies examining the identity discussions of Republican Turkey reflected into fine arts such as operas ( Turan & Komsuoğlu, 2007) and paintings ( Bibina, 2000) bring metohodological novelty to the scholarly literature, despite the fact that there is no such study scrutinizing the image of Europe in Turkish fine arts.

On the other hand, there is a strong tendency in the literature to examine political discourses at a rhetorical level without necessarily working on actual speeches ( Brewin, 2000; Helvacıoğlu, 1996; Heper, 2004; Keyman, 2006; Kösebalaban, 2007; Müftüler-Baç, 2000; Oğuzlu, 2002; Polat, 2006). Therefore, most of the studies with a claim to scrutinize political discourses are not necesarilly based on empirical research. A similar trend is also visible in other genres including history ( Kuran-Burçoğlu, 2003; Tachau, 1964; Yapp, 1992), and sociology ( Ahıska, 2003; Keyder, 1993) where assertions are not actually substantiated with empirical findings. Besides, the scholarly works that benefit from speeches and other texts do not necessarily provide an analytical discourse analysis thoroughly examining the meanings of political/cultural expressions in different political/cultural contexts, but rather pragmatically employ the speeches of certain political figures as a confirmation tool for authors’ arguments. Overall, there is an evident lack of a study that offers a (critical) discourse analysis by comprehensively examining European and Turkish political speeches and other texts to come up with a comparative perspective on identity depictions between Turks and Europeans during the Republican period.

### European representations of Turkey during the Republican era

This section scrutinizes how (or whether) the establishment of the Turkish Republic altered the Turkish image in Europe. There are two main trends in the literature. Accordingly, the bulk of the scholarly works dealing with the Republican era argue that the pro-Western reforms in Turkey were praised by some circles in the West, but the historical image of Turks in the eyes of Europeans did not necessarily change. In response, a minority of scholars focus on a number of European figures who believe in Turkey’s capability to become ‘like’ European.

According to Kuran-Burçoğlu (2003: 33–34), Atatürk aimed to dismantle the historical image of Turks in Europe by adopting the principles of secularism and non-intervention. Through Secularism, Atatürk believed Turkey would negate its image as the religious other of Europe. In other words, “Islam which had always been a significant issue that had made the Turks seem different in the eyes of the Europeans would cease to be an important issue” ( Kuran-Burçoğlu, 2003: 34). Second, Atatürk declared to the Western world that new Turkey would not be a revisionist power in order to address the long-standing European fear of “the expansionist Turk” (Ibid). these reforms, Kuran-Burçoğlu (2003: 36) argues, were impressive yet insufficient to have a remarkable impact upon European perceptions, since the historical negative image of Turks proves to be “deeply rooted” in European society.

Another pro-Western reform, the adoption of latin alphabet is evaluated by Wood (1929) as a positive step to help bridge the cultural divide between Turkey and the West, but Wood too admits that it does not necessarily erase Turkey’s ‘outgroup’ status in the eyes of the West. Wood (1929: 195) claims that Turkey has always been considered an ‘out group’ by the West due to the differences in ‘government, religion, the organization of the family, language, dress’. Hence, breaking the language barrier by latinizing the alphabet only helped to build an ‘understanding and good will between the Western nations and the Turkish “out” group’ (Ibid). Robins (1996: 65) reaches a similar conclusion as he admits that Turks were and are still considered by Europeans as ‘peculiar people’, ‘not authentically of the West’, ‘an outsider, an interloper in the European community’, despite the Republican attempts for Westernization.

The scholarly literature mainly argues that the Republican reforms failed to make a decisive impact in Europe, because Europeans were either unconvinced or unwilling to admit that such a top-down transformation would trigger an identity change in Turkish society. Zarakol (2010: 112) claims that Europeans certainly did not expect a Europe-friendly state would emerge out of a national movement which had fought against Europe a couple of years earlier. Western powers secured a fear that the new Republic would either usurp the powers of the Caliphate to mobilize the Asian Muslims or support Communism ( Zarakol, 2010: 132). Besides, Western governments, according to Criss (2008: 83), were rather suspicious of the Westernization process in Turkey since they did not believe the regime would last long enough to maintain the reforms. Most European ambassadors refused to transfer their residence from Istanbul to Ankara, the capital of new Turkey until 1931 (Ibid). European press was mostly sceptic of the success of a top-down reform process. There were two tendencies in the European press towards the Republican reforms: the reforms were either stigmatized as the impositions of an authoritarian leader or they were regarded as a positive step doomed to fail ( Zarakol, 2010: 139). In the 1930s, the British press started to appreciate Turkish modernization, but still did not locate Turkey within Europe. Zarakol (2010: 139) gives the example of *Contemporary Review* that calls Turkey and Japan as the most modern countries of Asia. Besides, the Republican transition was not necessarily a welcoming sight for Europeans either. Waterfield (1973: 199) intriguingly depicts the representations of the Ottomans and the Republic in the eyes of Louise Loraine, the wife of a British diplomat, through a comparison of (Ottoman) Istanbul and (Republican) Ankara in 1934. Loraine was frustrated to have to leave the exotic Ottoman city of Istanbul for the Republican Ankara, a ‘Godforsaken hole’ having ‘the atmosphere of an internment camp’ (Ibid).

Moreover, the approaches of major European governments regarding Turkey’s inclusion to an early version of European Union were rather pragmatic than identity-based ( Barlas & Güvenç, 2009: 431). French Prime Minister Aristide Briand’s proposal for European Union in the 1930s excluded Turkey for it was geographically located outside the European continent. Turkish statesmen protested Briand accusing him of drawing arbitrary boundaries of Europe, and claimed that “Turkey was geographically in Europe, since it was bounded by two European seas: the Black Sea and the Mediterranean” ( Barlas & Güvenç, 2009: 432). Briand’s approach to Turkey did not actually represent the European views, since Germany, Italy and Greece advised the inclusion of Turkey to the project. However, theythey strategically aimed to either include Turkey in their political and military bloc or prevent it from sliding to orbit of their rivals ( Barlas & Güvenç, 2009: 435).

Not only European politicians, bureaucrats and press, but also European public and artists were either unaware or unwilling to consider the Republican transformation as a pro-European identity change in Turkey. For instance, Brewin (2000) highlights the lack of interest in European society towards the emerging Republican Turkey. He claims that most Europeans were ‘indifferent to, and ignorant of, Turkey as a subject of history or as a model of Westernisation’ ( Brewin, 2000: 103). This indifference was especially reflected into arts and literature for European artists chose to remain loyal to the long-standing image of the Turk. For example, discussing the representations of Turks in Norway through novels, Tahir and Türker (2000: 74) claim that Norwegian view of Turks did not necessarily change when the Turkish Republic replaced the Ottoman Empire, because “even though the regime and borders have changed people are the same: they are Turks”. Tahir and Türker (2000: 75) highlight that in some instances Turkish reforms in the Republican era were praised, but then it triggered debates about ‘Turkey’s liminal position between the East and the West and its ensuing identity crisis”. Sabatos (2000) reaches a similar conclusion relying on the Czech and Slovak literature. Sabatos (2000: 261) argues that the representation of Turks as “the dangerous and alien force” in Czech and Slovak imaginary did not necessarily change with the demise of the Ottoman Empire. Although Czechoslovakia and the Turkish Republic shared no borders and had little contact during the interwar years, Czech and Slovak authors continued to depict ‘Turks’ as a symbol of foreign elements that threaten Czechoslovak sovereignty and intriguingly perceived ‘Jews’ as foreign spies working for Turks ( Sabatos, 2000: 262).

Discussing the impressions of a Bulgarian painter, Vladimir Dimitrov, about his visit to Istanbul in 1926, Bibina (2000: 381) reflects Dimitrov’s disappointment when he found a ‘modern’ Istanbul instead of an ‘Oriental’ one. The Republican elites’ efforts to create a Western society frustrated the painter who wished to see Turks as Oriental people rather than Western/European. He nevertheless chose to depict Istanbul in his paintings according to his own Oriental imagination rather than what he observed. Therefore, “where reality didn’t meet his expectations, his imagination added the “missing” aspects” ( Bibina, 2000: 384).

Murgeseu’s (2000) representation of Turks in Romanian history textbooks stands out as a minority in the literature. Murgeseu (2000: 283) claims that the books written in the 1930s depicted Republican Turks as “less other” than the Ottomans and praised Atatürk as the liberator of Turkey from “the Greeks” who overthrew the Sultan and established “a democratic republic.... in a European manner”. After the Communist takeover, Romanian history books approached the Republican era from a rather Marxist lense depicting the Republican elite as an emerging national bourgeoisie aiming to modernise the country after its liberation from a military-feudal regime and foreign invaders ( Murgeseu, 2000: 284). The history books written after the Cold War abandoned the Marxist rhetoric and returned to the discourse of the 1930s focusing on the European character of the Turkish reforms (Ibid).

There are only a couple of scholarly works emphasizing Turkey’s capability to become European with reference to the views of notable European figures. The most notable person highlighted in the scholarly literature is Richard von Coudenhove-Kalergi, an Austrian-Japanese politician and the pioneer of the idea of Pan-Europe, who once considered Turkey as a military threat to Europe in 1923, decided to include Turkey within political Europe after his visit to Turkey in 1934 ( Barlas & Güvenç, 2009: 438). He depicted the Turkey of Atatürk in the 1930s as “a compensation for what had been lost to Europe in the north and in the east as a result of the First World War” (Ibid). According to Barlas and Güvenç (2009: 438), Coudenhove-Kalergi was convinced that Turkey adopted European culture and became a member of European civilization through Republican reforms. Similarly, Yapp (1992: 195) refers to Alexandre Blacque and David Urquhart who claimed European success was not peculiar to the situation of Europe, and the characteristics of Europeans could be copied by others. According to Yapp (1992: 155), the likes of Blacque and Urquhart would hence claim that Turkey developed a capability to “become like European” thanks to the Kemalist reforms and the reforms by succeeding statesmen.

Finally, there was an evident sympathy towards Atatürk’s Turkey in the Nazi Germany, which could also be considered as a continuation of the 19th Century German Turkophilism. Ihrig (2014) claims that Atatürk constituted a crucial source of inspiration for Hitler in his endeavor to remake Germany along nationalist, secular, totalitarian, and ethnically exclusive lines.

### Turkish representations of Europe during the Republican era

The literature about Turkish perceptions on Europe during the Republican period is relatively understudied. Only several sources attempt to answer how the Republican Turks viewed Europe. Nevertheless, the extant literature still manages to offer four different explanations to the representations of Europe/West ^2^ in Republican Turkey.

The most popular scholarly answer is the ambivalence in the Republican understanding of Europe or the West, viewing it both as a threat and a model. For instance, according to Helvacıoğlu (1996: 516), the Republican regime perceived Europe both as a foreign invader and collaborator with the Ottomans, and as a model for Turkish Westernization. This duality laid the foundations of today’s perceptions towards Europe as “both the enemy and the object of desire” ( Helvacıoğlu, 1996: 518). Studying the evolution of Occidentalism (representation of West in the non-Western minds) in Turkey, Ahıska (2003: 367) traces a similar ambivalence in the Kemalist reading of Westernization. Accordingly, the Republican regime acknowledged Western civilization as superior to the Ottoman heritage, but also considered it a threat due to its morality, class struggles and imperialism (Ibid). Nevetheless, the Republican elite chose to adopt Westernization for it was viewed as the source of progress, despite the threats it poses to the Turkish society. This argument resonates with the notion of “Westernization in spite of the West” which was even influential in the Republican architecture. Bozdoğan (1998: 139) points to a paradox of the Republican elite who embraced European architecture as a token of Turkish modernization but introduced this new Europe-inspired architecture with an anti-imperialist and anti-orientalist discourse. Bozdoğan (1998: 140) explains that cubical and prismatic forms used by the Republican architects not only represented Turkey’s withdrawal from “Şarklılık” (Easternism), but also emphasized its total independence. Kösebalaban (2007: 88) calls this dual positioning as ‘defensive modernization’ which requires the adoption of Western civilization and abandonment of local traditions and moral codes in order to stay strong against the West. Referring to the works of prominent writers of the period such as Ahmet Ağaoğlu, Kösebalaban (2007: 89) depicts Turkish modernization as “Westernization for the sake of resisting the West” with an eagerness to learn from “the enemy” rather than integrate. Accordingly, Europe was perceived as the center of the civilization, which Kemalists strived to become a part of; though it was also considered a threat to Turkish independence and national integrity ( Kösebalaban, 2007: 90). Similarly, Bilgin (2009) indicates that Turkish adoption of Westernism/Europeanism was largely in reaction to ‘Early republican insecurities vis-à-vis European/international society’. The West/Europe used a civilizational discourse to leave Turkey out from the West, which according to Europeans proved that Turks did not deserve to survive as an independent nation ( Bilgin, 2009: 117). Hence, Turkey embaced Westernization to stand against Western imperialism. According to Zarakol (2010: 144), the Republican elite was aware of the costs of adopting Western values, but persistent on entering the ‘modern civilization’, they were willing to pay the price for it.

Second, the Republican elite believed the West had certain Orientalist prejudices against Turkey mainly ‘because of the difference in costume’ which needed to be addressed ( Zarakol, 2010: 147). This is why cosmetic reforms such as the replacement of Fez with Western-style hat were introduced to erase the ‘Orientalist’ notions in the West (Ibid).

Third, Ergin (2010) points to the dividedness of the Turkish public towards Europe/West during the Republican period. She analyzes two different characters from the Republican period, a Kemalist and an Islamist from Orhan Pamuk’s novel, *Snow*. The characters reflect a profound split within the Turkish society both towards the new Republic and Europe/West. The Kemalist character, also called *Jacoben* since the French Revolution was considered to be a source of inspiration for Kemalists, ‘openly admire(s) Western ideas for the sake of national progress’ and blames Islamists for the backwardness of the society ( Ergin, 2010: 253). The Islamist character, on the other hand, openly criticizes the West(ern Europe) for posing political and cultural threats against Islam, and stigmatizes pro-Western/Kemalist Turks as ‘the slaves of the ruthless European culture’ ( Ergin, 2010: 255). Ergin’s research is particularly valuable as it reminds that the Republican reforms failed to homogenize the Turkish public; they instead resulted in a sharp division of the society still visible today.

Finally, the literature poses the question of whether the Republican regime viewed Europe as its other, since it was essentially nationalistic. Bora (1997: 58) answers by saying an unqualified ‘no’. He claims that Turkey did not have any ‘external others’ especially in the initial years of the Republic. The main purpose of the Republican elite was to establish a new Turkish identity comprising Western identity which made it impossible to view Europe or the West as Turkey’s other (Ibid). Bora (1997: 58) quotes the words of Hamdullah Suphi; “In the Turkish Revolution, Europe was defeated, but Europeannes became victorious”. Similarly, Zarakol (2010) explains that the reforms fueling Turkish nationalism were considered in sync with European identity. Turkish nationalist formations such as *Türk Ocakları* were ordered to operate according to the dual principles of nationalism and Westernization. The words of its founder, Hamdullah Suphi are quoted by Zarakol (2010: 144) too as evidence for it: “We only became aware of our Turkishness when we approached Europeanness, and we will be Turks as long as we feel European”. Likewise, Heper (2004: 4) claims that founders of the Republic aimed to create a ‘new Turk’ free from religious dogmas, the main reason behind the demise of the Ottoman Empire. A ‘new Turk’ was created through the adoption of Western norms and European values ( Heper, 2004: 5).

Clark (2013)’s analysis supports the ‘pro-European’ claims to the Republican understanding of Turkish nationalism. Clark (2013: 207) claims that Atatürk launched a “pure Turkish” movement to free the language from Arabic and Persian vocabulary and grammar, introduced a Roman alphabet to replace the Arabic script, and rejected the Arabo- Persianate literary tradition in favor of the European. Clark (2013: 207–8) argues that in such a political environment, one might assume that the Republican regime would reject European writers such as *Loti* and *Farrère* who idealized Ottoman Muslim Turkey; but on the contrary, both writers were commended by the Turkish government for their invaluable contributions to Turkish literature. On the other hand, Aktürk (2007) claims the opposite. Researching Turkish history textbooks published during the republican era, Aktürk (2007: 350) argues: ‘many tropes of official Turkish historiography depict Turks as the opposite of Europeans, therefore, not only non-European, but anti-European’. However, he does not necesarily explain how ‘pan-Turkist’ rhetoric in history text books also translates into an ‘anti-European’ discourse.

### The focal issues

The four focal issues under scrutiny of the project, namely civilization, status in international society, nationalism and state-citizen relations have certain reflections in the prevailing literature. First of all, there is a dominant tendency in the scholarly literature to examine the focal issues through the prism of Republican Westernization and Turkish reflections on it, while there is a comparably less focus on European perspectives. Second, the extant literature shows a remarkably more attention to civilization and status in international society than nationalism and state-society relations. The Republican reforms were largely associated with the Turkish aspiration to locate the new Turkish Republic in the Western civilization and gain international recognition as a Western power while denying the Islamic tradition reminiscent of the Old Turkey, i.e. the Ottomans. Although not as popular as civilization and status in international society, nationalism proves to be another popular term highlighted in the literature, since the nationalistic nature of Republican reforms is widely debated. Besides, it is even possible to trace overlaps among focal issues for most scholarly works emphasize the tendency of the Kemalist elite to justify nationalist deeds with entering ‘civilization’ and gaining international recognition ( Aytürk, 2004; Barlas & Güvenç, 2009; Keyder, 1993; Polat, 2006). In this respect, the Republican inclination to develop a ‘pro-European’ nationalism could be interpreted as a desire to be part of the Western civilization and gain international legitimacy. Finally, in comparison to the first three focal issues, there is a relatively less attention to state-citizen relations. Here, the focus is rather on the suppression of non-Turkish identities with a particular emphasis on the Greek-Turkish population exchange, the Sheikh Said rebellion and the Capital levy, while there is a clear gap in the literature on how Europeans approached these events and how European perceptions on Turks were affected by these developments.

***Civilization***. There is a considerable amount of reference to ‘civilization’ in the literature, since the main goal of the Turkish Republic was determined by Atatürk as ‘reaching the level of contemporary civilizations’. Key developments in the Republican era such as the abrogation of Sultanate and Caliphate, the adoption of Latin alphabet and Western-style education, and the development of Football in Turkey, the establishment of the Turkish Opera were the main focus of the scholarly literature to denote the resoluteness of the Republican elite for a pro-Western transformation of the society in order to ‘enter civilization’. Bora (1997: 58) explains that the Ottomans and Islam, meaning the old Turkey, were viewed as the ‘other’ of the new Republic. Therefore, Republican reforms aimed to dismantle the old regime by erasing its traces from the state apparatus. For instance, the adoption of Latin alphabet is considered by many as an attempt to purify Turkish language from Arabic and Islamic elements ( Aytürk, 2004; Aktürk, 2007; Clark, 2013). Similarly, Zarakol (2010: 147) explains that the headgear reform was viewed as the symbol of the new Turkey, while the fez was associated with the old Turkey as ‘a symbol of ignorance, backwardness, fundamentalism, and hatred of civilization’. According to Lewis (1961: 410), the Headgear revolution and others promoting Western attire were particularly justified with reference to ‘civilization’. Islamic dress was condemned as the reflection of backwardness and religious dogmas; instead, Western-style dress was introduced as the reflection of a civilized Turkish society ( Lewis, 1961: 411). Tekeli (1998: 165) offers an intriguing perspective discussing the Republican logic behind the changing of the Turkish capital from Istanbul to Ankara. He considers the change as a radical decision, since Istanbul had proved to be the most Westernized city of the Ottomans (Ibid). By denying it, the Republican elite thus dismissed the Ottoman Westernism as ‘corrupted’ and aimed to construct the ‘true’ Westernism in a new capital city; Ankara (Ibid).

Focusing on the development of state policies towards football in Turkey, Şenyuva and Tunç (2015: 570) assert that the Turkish Republic invested in the development of football to eras the long-standing Ottoman image as ‘the sick man of Europe’ and construct a modern and ‘westernised’ image for the new Turkey. For this purpose, Turkish policymakers invited Dr Carl Diem, the general secretary of the organising committee of the 1936 Olympics, to write a proposal for improving the Turkish sports system. The Turkish Sports Institution (Türk Spor Kurumu, TSA) was established in 1936 on the model proposed by Diem’s report of 1933 (Ibid).

Studying the Turkish approach to Opera in the late Ottoman and the early Republican years, Turan and Komsuoğlu (2007: 7) argue that the Turkish Republic not only viewed Western music as a modernization tool; but also as a means to consolidate Turkey’s new “national” identity. Reform in Turkish music became a major part of the Republican reform process aiming to create “a new country with a new culture” ( Turan & Komsuoğlu, 2007: 25). According to Turan and Komsuoğlu (2007: 19), the establishment of the Turkish Opera hence proved to be the last major effort of the Republican regime to develop a western musical culture in Turkey.

Döşemeci (2013: 30) asserts that the rhetoric of ‘civilization’ served as a convincing justification for a top-down revolution in Turkish society entailing ‘a radical restructuring of a multiethnic theocratic empire into a homogenous secular nation-state, as well as ... [the] transform[ation of] the daily lives of the Anatolian people’.

According to Nas (2001: 181), modernization during the Republican era was based on the premise that ‘the Western civilization had to be taken as a whole, together with its technology, culture and social and political institutions’. Therefore, the reforms aimed for a societal transformation creating a new national identity which was modern, secular, Europe-oriented and nationalistic ( Nas, 2001: 180). Polat (2006: 515) explains that this forceful identity creation was considered compatible with the Enlightenment rationality, but Western modernity then moved beyond it and embraced multiculturalism ‘leaving the Kemalist project out in the cold’.

Moreover, Keyman (2006: 207) explains that the Republican elite resorted to a top-down and rapid modernization of the country to quickly catch up with the level of civilization in the West. Keyman (2006: 207) however contends that it was not sufficient to accomplish a full-fledged modernization through technological development only; it was also necessary to ‘construct a national identity compatible with the will to civilization’. Due to the urge to overcome the problem of the “time lag” between Turkey and the West as fast as possible, the Turkish elite prescribed a one-size-fits-all ‘modern and secular’ national identity resulting intoa societal “modernization without democratization” ( Keyman, 2006: 208).

There is a particular reference to the Republican attempt to deal with *excessive Westernization*. Zarakol (2010: 143) informs that the Republican elite viewed (Western) civilization together with autonomy, sovereignty, and power: “A country could not have the latter without the former”. Adopting Western reforms without losing independence constituted the key Republican strategy in persuading ‘skeptics and detractors’ about the necessity of Westernization (Ibid). Similarly, Ahıska (2003: 366) argues that the Republic assumed a guardian role to protect the ‘less civilized’ and ‘infantile’ population from the ‘dangers of too much Westernization’. This protectionism primarily stemmed from the uncertainty of the Republican elite about the effects of Westernization in Turkey (Ibid). That’s why Westernization was both considered as ‘necessary’ and ‘dangerous’ at the same time ( Ahıska, 2003: 366–367).

According to Keyder (1993: 24), the Republican elite “invented” nationalism as the ideological tool to protect Turkish society from the ill effects of westernization. However, the notion of national identity had no historical roots in Turkish context, therefore “it had to be taught afresh” (Ibid). This simultaneous ‘learning and teaching’ process eventually caused popular reaction in defense of local culture against ‘a transformed (hence alien) and authoritarian great culture’.

Finally, according to Döşemeci (2013: 32), the Republican logic of ‘civilization’ had three distinct features. First, civilization was not only based on economic and military developments, but it also had ‘a cultural and spiritual’ character (Ibid). Second, the Republican elite did not simply wish to ‘emulate the other for its own advancement’ but tried to become the other ‘gaining acceptance by this other as a member’ (Ibid). Finally, the civilizational logic of Turkey charged the European other to arbitrate over Turkey testing its capability to become a European other ( Döşemeci, 2013: 33). This Republican interpretation of civilization, Döşemeci (2013: 33) argues, constitutes the backbone of Turkish-European relations ever since.

***Status in international society***. The scholarly literature pays particular attention to the Republican motivation to gain international status through Westernization. Many scholarly works associate the reforms with the objective of gaining international recognition and the acceptance of Europe. For instance, referring to Toynbee’s observations of the Republican period, Robins (1996: 64) argues that Turkey’s quest for Westernization in the form of an ‘abandonment of one civilization and way -of life and the adoption of another’, primarily stemmed from the desire to gain international status and recognition. Keyder (1993: 24) argues that the Republican elite introduced a westernization process with the objective of assimilating Turkey into an ‘imagined European area’. Similarly, Nas (2001: 181) claims that Atatürk’s reforms ‘laid down the foundation for Turkey's eventual integration into Europe’. Moreover, Müftüler-Baç (2000: 28) asserts that the pro-Western reforms aimed to make Europeans accept ‘the new Turkish state’s Europeanness’. Similarly, Aytürk (2004: 2) argues that the reforms were undertaken to pressurize ‘the Western nations… to acknowledge the Turks as part of their family, as a nation which contributed most generously to their civilization’. For instance, the highly controversial Sun-Language theory was primarily based on the Turkish desire to be acknowledged ‘as part of the west in linguistic and historical terms, which, it was hoped, would pave the way for modern Turkey’s full accession to European civilization’ ( Aytürk, 2004: 19).

Barlas and Güvenç (2009: 431) raise a similar claim that the Republican urge to redefine Turkey as Western/European essentially stemmed from “the Turkish pursuit of recognition as a European member of international society”. Turkey’s effort for the League of Nations membership was particularly associated with the objective of gaining international status ( Barlas & Güvenç, 2009: 432). Likewise, according to Zarakol (2010: 135–136), the Montreux Convention of 1936, the Balkan and the Sadabad Pacts along with the League membership reflect the Republican objective to be recognized internationally as the protector of status quo and peace.

Oğuzlu (2002: 581) brings a security perspective to the identity discussions arguing that the Republican regime sought external recognition as ‘European’ to guarantee its security: ‘the more the Europeans recognized Turkey as 'European’, the more ‘secure’ Turkey would feel. And the more the Europeans saw Turkey as vital to European 'security,' the more the Turks thought of Turkey as ‘European’. Bilgin (2008), and Bilgin and Bilgiç (2012) draw a similar conclusion. Bilgin (2008: 144) emphasizes that the Republican elite considered the modernization project and ‘gaining respect in the eyes of the civilized world’ as a solution to the security problems of the new Turkey. Similarly, Bilgin and Bilgiç (2012: 122) argue that achieving Enlightenment and meeting the standard of civilization would grant Turkey an international recognition of its sovereign equality and keep European colonialism away from its territories. Therefore, through Westernization, Turkey would free itself from future European interferences ( Bilgin, 2008; Bilgin & Bilgiç, 2012).

Finally, Döşemeci (2013) establishes a direct connection between the two focal issues claiming that the Turkish urge for ‘civilization’ stemmed from the desire for ‘status in international society’. He thus argues that the Republican aspiration to catch up with the level of contemporary civilization was primarily motivated by the aim to ‘end the liminal status of the Turk’, an Ottoman legacy of being ‘caught between integration within a system of European states and exclusion as its uncivilized other’ ( Döşemeci, 2013: 30).

***Nationalism***. Nationalism is also a popular term used in the literature since Republican reforms were mostly depicted as nationalistic as well as pro-Western. Therefore, the Republican reforms were not only introduced with the objective of catching up with the Western civilization and gaining international status, but also with the particular aim of mobilizing the masses under Turkish nationalism and even Pan-Turkism. For instance, Tachau (1964: 200–201) argues that the adoption of Latin alphabet and other language reforms were mainly geared towards fueling nationalism into the society with the aim of purifying the Turkish identity from foreign elements. Republican inventions such as Sun-Language Theory claiming Turkish as the mother of all languages were viewed as a remedy for treating the inferiority complex ‘instilled by the prejudices of European historians’ (1964: 202). Similarly, Aytürk (2004: 11–16) explains that the Republican Turkey tried to justify the ‘superiority’ of Turks through linguistic research. For instance, Ahmet Cevat Emre claimed to have found in his research that ‘an ancient Turkic language was the original Indo-European language, carried to Europe from the Eurasian steppes’ ( Aytürk, 2004: 11). Moreover, Turkish linguists worked on the state sponsored ‘Sun-Language Theory’ to prove that Turkish was ‘the mother of all languages’ ( Aytürk, 2004: 15–19).

Relying on the Turkish history textbooks of the Republican period, Aktürk (2007) claims that Turkey has never had an official claim to European identity, but a Pan-Turkist one. He claims that Atatürk’s formulation of Turkish identity was not based on European values, but it was rather built upon ‘a myth of origins in Central Asia, which was construed as being implicitly supportive of "Turkic imperial" imagination’ ( Aktürk, 2007: 349). The Ottoman legacy was denied since ‘the Ottomans were associated with religious obscurantism, a decadent cosmopolitanism that had corrupted the Turkish "essence" with Arab and Persian elements, scientific and economic backwardness, military defeat, and collaboration with foreign invaders’ ( Aktürk, 2007: 358). However, Aktürk (2007: 358) asserts that Republicans did not replace the Ottoman identity with a European one; they instead turned to pre-Islamic Central Asian and Anatolian history to ‘Turkify’ the new Turkey’s identity. After the death of Atatürk, Turkey under İsmet İnönü rather invested in the exploration of the Greco-Roman roots of Turkish identity, through the translation and official dissemination of Western classics from ancient Greek and Latin ( Aktürk, 2007: 359). Aktürk (2007: 360) however contends that this Greco-Roman shift in Turkish identity construction does not indicate the European vocation of the new Turkish Republic, but rather points to the Turkish official desire to promote ‘Turkish Humanism’.

Ergin (2008: 831) associates Republican nationalism with racism highlighting ‘the republican fascination with whiteness’. Measuring Turkishness through skin colour exams, craniological measurements, or blood types was meant to ‘produce a scientific basis for participating in the project of modernity as equal partners in an international order of advanced nationstates’ ( Ergin, 2008: 831). Therefore, even the racist elements in the Republican interpretation of nationalism were justified with the urge to be recognized by the West as a legitimate actor.

Finally, as pointed out previously, the extant literature depicts the Republican understanding of Turkish nationalism as ‘pro-European’. With the exception of Aktürk (2007), the scholarly literature generally claims that the Republican understanding of Turkish nationalism was designed to encompass Western or European identity ( Bora, 1997; Clark, 2013; Heper, 2004; Keyder, 1993; Zarakol, 2010). Keyder (1993: 24) particularly explains that the Republic suppressed “the ambivalence and defensiveness of nationalism vis-à-vis the West” and appropriated it “as a purely modernizing ideology” compatible with the West. Here, it is possible to trace connections among focal issues since the literature associates the promotion of a pro-European nationalism with the Republican aspiration to be included in the Western civilization. Ergin’s (2008) analysis establishes a similar connection between racist policies to create Turkish nation and the Turkish quest for recognition by the West. Another example associating nationalism with another focal issue; civilization is raised by Bora (1997: 58) who finds traces of Orientalism in Turkish nationalism especially in the downgrading of Islamic tradition in social and political life.

***State-citizen relations***. The prevailing litrature stresses the suppression of non-Turkish ethnic identities.. For instance, Polat (2006: 515) explains that the new Turkish Republic deemed it necessary to establish a homogeneous nation-state with a common civic culture through the suppression of the pre-modern or regressive periphery. Nas (2001: 180–181) contends that although Turkish identity was supposed to be based on civic attachment ("Happy is the one who calls himself a Turk”), it was based on subsuming all ethnic groups under a general Turkish identity.

Heavy-handed policies towards minorities are emphasized in three main political events: the compulsory population exchange between Turks and Greeks in 1923–1925, the Sheikh Said rebellion in 1925–1926 and the Capital levy issued to non-Muslim Turkish citizens during the Second World War.

The population exchange is considered by many scholars as an example of the Republican attempt to homogenize the society ( Iğsız, 2008; Keyder, 2003). Especially, Keyder (2003: 51) argues that population exchange was a conscious effort to create a coherent narrative of the past and a homogeneous Turkish nation.

The Sheikh Said rebellion is depicted as a religious as well as a nationalist uprising through which Kurdish tribes aimed to protect their national and religious identities against the repressive policies of the Republic ( Olson, 1989; Yeğen, 1996). According to Olson and Tucker (1978: 197), the rebellion also fueled anti-British sentiment in Turkey as Britain was accused of orchastrating the rebellion. Turks, especially Atatük, believed that “the British, seeking the oil deposits of Mosul, had roused anti-Turkish feeling among the Kurds hoping to use this as a means of pressuring Turkey in relinquishing Mosul” (Ibid).

Finally, the Capital levy is viewed in the literature as a concrete example of the Republican oppression towards minorities. Ergin (2008: 829–830) considers its introduction as a crucial indicator of state sponsored racial discrimination. Aktar (2000) views the Capital levy as an extention of the Republican government’s ‘Turkification’ policies that further isolated its non-Muslim citizens. Oran (2001: 397) on the other hand associates the Capital levy as a reflection of the ultra-nationalist initiatives of pro-Nazi Germany elements within the Republican government such as Prime Minister Şükrü Saraçoğlu, the chief architect of the levy.

On the other hand, litrrature also focuses on Turkey’s open-door policy to Nazi-persecuted Jewish scientists rather as a positive example. There is a vast literature on how Turkey opened its borders to brilliant Jews running away from the Nazi Germany for humanitarian reasons ( Grothusen, 1981; Önsoy, 1998; Reisman, 2006; Söyler, 1987). The Republican motivation behind this policy was however pragmatic, according to Ergin (2009: 111), since it was believed that “émigrés would rekindle a new Renaissance in Turkey and bestow European modernity to its rightful owners in its own cradle”. The Republican regime was particularly persistent to use these scholars in its quest for dicovering “Turkish essences in historical, linguistic and racial studies” ( Ergin, 2009: 112–113). 

These events provide concrete examples for the representations of Republican Turkey in Europe. However, despite numerous academic sources dwelling on these key events, there is no scholarly work discussing how these events (re)shaped the European perceptions of Turkey.

Overall, the prevailing literature treats the identity representations of Europeans and Turks rather separately. It either concentrates on how Europeans viewed Republican Turks in comparison to the historical image of Turks, or how Republican Turks viewed the West/Europe. A comparative study is necessary for scrutinizing how certain events during the period (re)shaped the perceptions of Turks and Europeans towards each other. Besides, there is a need for a study particularly focusing on how the Republican reforms affected the Turkish image in Europe. The events in the Republican era, mentioned above, provide a solid basis for researching the European approach to Turkish identity and culture, but the existing academic sources evaluating these events rather overlook this aspect in their analyses.

## The Third Period (1946–1998)

The third period (1946–1998) starts with the end of the Second World War and lasts until the officialization of Turkey’s EU membership candidacy. Major political developments that took place during this period include Turkey’s memberships in Western institutions such as the Council of Europe (1949) and NATO (1952), the signature of the Ankara Association Agreement initiating Turkey’s European vocation (1963), military coups in Turkey including the ones in 1960 and 1980 that damaged Turkey’s democratic credentials and the Luxembourg European Council in 1997 that denied EU candidacy to Turkey, amongst others. Moreover, key cultural developments during this period include the arrival of the first wave of Turkish guest workers in Germany (1961), Turkey’s first participation at the Eurovision Song Contest in Stockholm (1975), the release of the movie Midnight Express (1978) and Turkish oil wrestling became popular in Europe (1997). Apart from key events, there are also notable visits including the visits of İsmet İnönü, Süleyman Demirel, Turgut Özal and Tansu Çiller to Europe, and the visits of Konrad Adenauer, Charles de Gaulle, Elizabeth II, Richard von Weizsäcker and Margaret Thatcher to Turkey. These events and visits constitute crucial drivers of political and cultural interactions between Turkey and Europe. 

The extant literature divides this period into two, namely the Cold War and post-Cold War periods, and traces shifts in the representations of Turkey in Europe and vice versa. The literature particularly indicates a remarkable shift in European political discourses on Turkey after the Cold War. While Turkey’s Europeanness was not necesarily questioned by Europeans during the Cold War security context, Europeans increasingly questioned Turkey’s European credentials after the end of the Cold War ( Aybet & Müftüler-Bac, 2000; Çapan & Onursal, 2007; Challand, 2009; Keyder, 2006; Nas, 2001; Oğuzlu, 2002; Özoğuz, 2007). On the other hand, the extant literature does not indicate such a drastic change in the representations of Europe in Turkey. Turks continued to perceive Europe both as a source of inspiration and anxiety in the post-Cold War era ( Bilgin & Bilgiç, 2012; Helvacıoğlu, 1999; Kuniholm, 2001; Kuzmanovic, 2008; Macmillan, 2013; Pesmazoglu, 1997; Sezer, 2010). However, similar to the previous periods, there is still a lack of a detailed analysis on how Europe was perceived in Turkey and how Turkey was received by European society in a comparative fashion. There is hence an evident necessity for a comprehensive comparative study.

The extant scholarly literature during the third period is overwhelmingly informed by Politics and IR ( Aybet & Müftüler-Bac, 2000; Bilgin & Bilgiç, 2012; Bogdani, 2010; Buzan & Diez, 1999; Çapan & Onursal, 2007; Challand, 2009; Diez, 2004; Helvacıoğlu, 1999; Keyman, 2006; Krieger, 2006; Kuzmanovic, 2008; Levin, 2011; Macmillan, 2013; Mandel, 1995; McLaren, 2007; Müftüler-Baç, 1997; Nas, 2001; Oğuzlu, 2002; Öniş, 1999; Özoğuz, 2007; Pesmazoglu, 1997; Robins, 1996; Stone, 2002; Verney, 2007; Yanık, 2009) and Sociology ( Antoniou & Nuhoglu Soysal, 2005; Göbenli, 2003; Gülalp, 1998; Keyder, 1998; Keyder, 2006; Manço, 2000; Mastnak, 2000; Straube, 2000; Yanovski, 2000). In comparison to the previous periods, there is a remarkable decrease in the number of scholarly works informed by History ( Aktürk, 2007; Atakuman, 2010; Döşemeci, 2013; Yıldırım, 2014). Moreover, there are also a number of works informed by other genres such as Literary studies ( Aydın, 2003; Sabatos, 2000; Sezer, 2010; Soenen, 2000), Communication ( Buğday, 2003; Rougheri, 2000; Yanovski, 2000) and Sports ( Şenyuva & Tunç, 2015) that offer a nuanced approach to the prevailing literature.

The methodological gap in the previous period indicating a lack of (critical) discourse analysis based on comprehensive field research continues to be the case in this period. The bulk of the scholarly literature that claims to analyze political discourses in Turkey and Europe does not necessarily rely on original data. Although several studies scrutinize political discourses with reference to the speeches of prominent political figures in Turkey such as Turgut Özal, İsmet İnönü, Süleyman Demirel, Necmeddin Erbakan, Feridun Cemal Erkin and Fatin Rüştü Zorlu ( Atakuman, 2010; Bilgin & Bilgiç, 2012; Döşemeci, 2013; Robins, 1996), these studies rather use these texts as discursive tools to uphold their own arguments than comprehensively evaluating the meanings of political/cultural expressions in different political/cultural contexts. The literature also analyzes Turkish and European novels and other literary texts ( Aydın, 2003; Sabatos, 2000; Sezer, 2010; Soenen, 2000), Turkish and European press ( Buğday, 2003; Rougheri, 2000; Yanovski, 2000) and history textbooks ( Aktürk, 2007; Antoniou & Nuhoglu Soysal, 2005), although the main methodological focus of the literature remains on political discourses.

### European representations of turkey

The prevailing literature traces certain changes in the representation of Turkey in European politics following the end of the Cold War. European political elite refrained from approaching Turkey from an identity perspective due to the intensive security context of the Cold War. Therefore, European approaches to Turkey were primarily informed by security rather than identity. This facilitated Turkey’s inclusion in the emerging European security community. However, the post-Cold War political context necessitated Europeans to redefine the identity of Europe, which then triggered debates about Turkey’s place in Europe. Contrary to politics, European society however did not necessarily adopt a pragmatic approach to Turkey’s identity during the Cold War, since European literature and press depicted Turkey as ‘non-European’, and this trend continued after the end of the Cold War.

***The cold war representation of turkey in europe***. The scholarly literature traces three main tendencies regarding the Cold War imagery of Turkey in Europe. First, the Cold War context forced European governments to view Turkey as a necessary ally against the Soviet threat. Therefore, without questioning its ‘Europeanness’, Europe accepted Turkey as an integral part of the European security community ( Aybet & Müftüler-Bac, 2000; Çapan & Onursal, 2007; Challand, 2009; Keyder, 2006; Krieger, 2006; Nas, 2001; Oğuzlu, 2002; Özoğuz, 2007). Second, European society, however, treated Turkey differently. Scholarly works mainly trace a particular stereotypical image of Turks as “barbarians” and/or “non-Europeans” depicted in European literature ( Aydın, 2003; Sabatos, 2000; Soenen, 2000), press ( Buğday, 2003) and History books ( Antoniou & Nuhoglu Soysal, 2005) with a particular reference to political developments in Turkey including the 1960 and 1980 Military coups, and Turkey’s military intervention to Cyprus in 1974. Finally, the difficult encounters between Turkish immigrants and European public were directly translated into the European perceptions of Turkey ( Göbenli, 2003; Manço, 2000; Mastnak, 2000; McLaren, 2007; Müftüler-Baç, 1997; Robins, 1996; Straube, 2000; Yanovski, 2000).

The literature generally concurs regarding Turkey’s key role in the European security structure during the Cold War. Turkey’s inclusion in the Western security alliance also provided an enabling environment in its interactions with Europe, since nobody was in a position to question its European credentials given the Communist threat ( Aybet & Müftüler-Bac, 2000; Çapan & Onursal, 2007; Challand, 2009; Keyder, 2006; Nas, 2001; Oğuzlu, 2002; Özoğuz, 2007). Besides, as Diez (2004) indicates, nobody was questioning or redefining Europe’s identity either. According to Krieger (2006: 167), it was even a taboo within the EEC ‘to voice fundamental criticism of Turkey in public’ in order not to dissuade Turkish government from the Western Camp. For instance, the West German discourse on Turkey’s European identity essentially secured a pragmatic motivation to reinforce Turkish loyalty to the West ( Krieger, 2006: 169).

The historical other of Europe, namely Muslim Turks, was replaced with Communist Russia as a much higher threat during the Cold War. Challand (2009) argues that Communism as the new external other of Western Europe postponed, if not totally erased, identity debates about Turkey in European political circles. Examining the EU’s official documents since 1959, Challand (2009: 73–74) finds no reference to contestations against Turkey in terms of identity and culture, but rather the appreciation of Turkey’s Western identity during the Cold War period. Özoğuz (2007) and Krieger (2006) support this view by arguing that there were no cultural contestations against Turkey’s entry into the EEC during the Cold War. When Turkey applied for EEC membership in 1987, the main objections in Europe against Turkish entry were rather economic (economic instability) and political (human rights violations and democratic deficit) than cultural ( Özoğuz, 2007: 6; Krieger, 2006: 169).

European society’s understanding of Turkey and Turks during the Cold War was, however, very different from European politicians. The prevailing scholarly literature contends that Turks and Turkey were rather depicted in European novels, press and cinema as the ‘other’ highly affirmative of the historical image attributed to Turks. Aydın (2003) highlights two major tendencies in European novels, magazines, and cinema. First, the historical image of Turks as barbarians and Islamic fanatics were retained during the Cold War with a particular reference to the Crusades, the Fall of Constantinople, the Greek War of Independence, the Crimean War, and the Armenian Genocide ( Aydın, 2003: 314). Second, depiction of Turks in European literary texts was influenced by Turkey’s policies during the Cold War. For instance, in reaction to the Turkish policy of hashish production under state control in the 1960s and 1970s, Turks were increasingly depicted as drug-dealers in European and American novels ( Aydın, 2003: 313). Similarly, the 1980 coup made an impact on the representations of Turks in European novels. While, Turkish security forces were portrayed as ‘unprofessional’, ‘incompetent’, ‘clumsy’ and open to bribery in the 1960s novels, Turkish soldiers during the 1980 coup were described as ‘Mongol overlords crushing rustics under armed heels’ ( Aydın, 2003: 312–316). Aydın (2003: 320) also refers to the popular movies during the 1960s and 1980s such as Midnight Express and Lawrence of Arabia that create a strong image of Turks in the minds of the Western audience.

Sabatos (2000) traces a peculiar tendency in Czech and Slovak novels to highlight the danger of “Turkish invasion” even during the Cold War period although the Turkish threat had long passed. Sabatos (2000: 266) argues that the use of “Turkish threat” was a subtle way of criticizing the Soviet control and the excesses of Stalinism, ‘another “alien” force from the East that could not, of course, be directly criticized’. Some Czech novels even depicted the Soviet invasion of Prag in 1968 as a Turkish invasion ( Sabatos, 2000: 267). Novelists hence drew ‘metaphorical parallels between the Turkish invaders of the past and the Soviet invaders of the present’ to raise criticisms against the Soviet control over Czechoslovakia without alerting the Soviet regime ( Sabatos, 2000: 268).

Soenen (2000: 42) indicates an increasing interest in Belgium and Netherlands towards Turkish literature especially after the 1980 *coup*. Numerous Turkish novels were translated into Dutch after the *coup*, while such interest was almost non-existent before 1980. Turkish literature translated in the 1980s was essentially categorized by European publishers as either Asian or Middle Eastern ( Soenen, 2000: 38).

Buğday (2003) examines the representation of Turkey in the German magazine ‘Der Spiegel’ from 1946 onwards and finds that Turkey was generally considered as an ally of the West in the Cold War context, but not necessarily viewed as Western. Especially, the 1960 coup contributed to the image of Turkey as ‘Oriental and African peoples who are all together called “coloured”’, hence non-European ( Buğday, 2003: 301). The *coup* was considered to have shattered Turkey’s image as ‘a western-oriented modern state’ ( Buğday, 2003: 302). General Cemal Gürsel was depicted as ‘der Revolutions-Papa’ leading Turkey back to ‘the enlightened despotism of the Orient’ (Ibid). Similarly, the rhetoric of ‘barbarian Turks’ was further highlighted in reaction to Turkey’s intervention in Cyprus in 1974 ( Buğday, 2003: 306).

Scrutinizing the representations of Turks in Greek History textbooks, Antoniou and Nuhoglu Soysal (2005: 116) explain that the Turkish military intervention to Cyprus in 1974 among other events was used in Greek history books as a rationale for maintaining images of a Turkish ‘other’. The inclusion of such encounters in Greek educational materials, according to Antoniou and Nuhoglu Soysal (2005: 116), projects a historically permanent generic Turkish ‘other’ in Greek national narrative presented as ‘a mighty military force, whose civilizational and ethical values are questionable’.

Finally, voluminous scholarly works studied Turkish migrant workers in Europe mostly focusing on the impact of immigrants in Turkish image in Europe. Encounters with Turkish immigrants contributed to the image of Turks as the other of Europe. Müftüler-Baç (1997: 21) argues that immigration to Europe increased interaction between Turks and Europeans resulting into an ‘influx of Western values into Turkish culture’, while at the same time revealing the difference between two cultures. Manço (2000: 29) explains that the image of Turkish male in the eyes of European public directly reflects the image of Turkish immigrants: ‘backward and more often fundamentalist, violent, uninterested in his children’s education other than religious instruction, exploits social benefits’ and having close relations with Mafia. Turkish female is depicted as illiretate, submissive, veiled, victim of arranged marriage, victim of domestic violence and having no control over her own future ( Manço, 2000: 29).

Robins (1996: 66) even claims that European experiences with Turkish migrant workers feed into the Turkey-sceptic discourse that Turks can never be assimilated or converted into European culture. Besides, the sense of being overwhelmed by an alien (Turkish) culture resonates heavily with the assertion that Turkish migrant workers constitute ‘a kind of continuation (this time by economic means) of the Ottoman (Islamic) onslaught on Europe’ (Ibid). McLaren (2007) argues that the role of Turkish migrant workers in the construction of a particular image of Turkey in European minds considerably contributed to the burgeoning opposition within European public against Turkey’s EU entry. The extant literature hence extensively stresses European interactions with Turkish immigrants as a key indicator of European resentment towards Turkey. A notable exception is raised by Stone (2002: 187) who asserts that the presence of Turkish migrants in Western Europe since the 1960s feeds into the perception that ‘the Turks are already a part of the European scene’, since they constitute a sizeable portion of minorities in Western Europe.

***The Post-Cold War representation of Turkey in Europe***. The bulk of the extant literature regarding the European perceptions concentrates on the post-Cold War period. It is possible to trace two tendencies in the scholarly literature. First, politics-wise, the main argument is that the end of the Cold War shifted Europe’s focus from security to identity which put Turkey in an awkward position. Since the post-Cold War political environment led Europe to redefine its identity, Europeans increasingly questioned Turkey’s Europeanness. Second, scholarly works reveal a continued tendency in European press to depict Turkey as the other.

Aybet and Müftüler-Bac (2000: 566–567) argue that the end of the Cold War ‘challenged Turkey's position in Europe’ and resurfaced ‘centuries-old questions about Turkey's 'Europeanness'’. While considered within the European community during the Cold War that prioritized security over identity, Turkey found it difficult to locate itself in Europe after the Cold War ( Aybet & Müftüler-Bac, 2000: 570). The post-Cold War security understanding of Europe was not only based on the security of physical borders, but also on the objective of securing ‘a newly defined’ European identity and rediscovering ‘the notions of 'Europeanness' that were repressed at the start of the Cold War’ ( Aybet & Müftüler-Bac, 2000: 571). Therefore, in the absence of the Cold War security parameters, the perceptions of the 'Turk' as the 'other' of the European identity, ‘deeply embedded in the European consciousness’, resurfaced, and determined the EU’s policies towards Turkey in the post-Cold War era ( Aybet & Müftüler-Bac, 2000: 579–580). Keyder (2006: 72) too highlights the end of the Cold War as a game changer for Turkish-European relations, since ‘Europe’s boundaries had shifted from congruence with the line tracing the ‘iron curtain’ to one of civilizational divide – defined as religious difference’. This brought back onto the agenda ‘the uncertainty of Turkey’s inclusion – an uncertainty leaving Turks to feel like scorned suitors’ (Ibid). Similarly, Çapan and Onursal (2007: 105) emphasize that when the Cold War security context disappeared, Turkey’s place in Europe became contested. Müftüler-Baç (2000: 29) shares this point claiming that the end of the Cold War replaced ‘the ideological East-West conflict with ethnic, religious, and historical conflicts’ emphasizing Turkey’s non-Christian and hence non-European character. Besides, the increasing number of Turkish immigrants in European countries stirred hostilities against Turks ( Müftüler-Baç, 2000: 30).

Diez (2004: 329) claims that Turkey’s membership process after the Cold War brought an important challenge that necessitated to determine who was European and who was not. Likewise, Robins (1996: 65–66) points to the hybridity of Turkish identity creates an overriding image for Turkey as ‘an in-between place’. According to Robins (1996: 66), Turkish hybridity gives strong leverage to the opponents of Turkish entry into the EU who not only base their claims on the Islamic identity of Turkey but also refer to Turkey’s Asiatic origins back to Cengiz Khan.

Challand (2009) argues that the threat of Eastern European Communism as the external other of Western Europe during the Cold War was replaced, in the post-Cold War era, with the threat of Islam embodied in Turkey’s possible entry into the EU. The post-Cold War environment hence led to the re-definition of Europe along with the re-definition of Turkey which proved detrimental to Turkey’s place in Europe ( Özoğuz, 2007: 2).

Verney (2007) concentrates on the introduction of the Copenhagen criteria as a turning point for the categorization of Turkey as (non)European. Verney (2007: 215) stresses that Turkey’s strategic significance during the Cold War had encouraged its depiction as ‘European’ and facilitated its admission to other European organizations such as the OECD and the COE. However, after the introduction of the Copenhagen Criteria in 1993 that emphasized ‘democracy as the primary defining characteristic of a European state’, Turkey’s European credentials were increasingly questioned since ‘Turkey’s image has not corresponded with the democratic European ideal’ ( Verney, 2007: 216).

Öniş (1999) claims that in the post-Cold War era, Christianity became a defining component of European identity and ‘this dimension comes to the surface and plays a major determinant role’ in the EU’s approach to Turkey’s membership bid. According to Öniş (1999: 113), the factor of religious difference cannot be ignored when explaining the differential treatment of the CEECs and Turkey despite similar economic and political indicators. Similarly, Oğuzlu (2002: 587) argues that it was the question of Turkey’s European identity that ‘led to Turkey’s placement in the membership list far behind the countries of Central and Eastern Europe’, even though they were not far better than Turkey in terms of economic and democratic development.

Finally, scrutinizing the debates in the European Parliament from 1996 to 2010, Levin (2011: 182) argues the issue of Turkey’s EU accession constituted ‘a key site for the contestation over what it means to be European and where Europe’s borders lie’. According to Levin (2011: 185–186), the debates between 1996 and 1999 particularly focused on whether Turkey’s European credentials could be endorsed if it complied with the Copenhagen political criteria. However, many right-wing conservative MEPs considered the political criteria as irrelevant, since Turkey was not a European country ( Levin, 2011: 187).

On the other hand, the literature traces a continued tendency in European press to treat Turkey either as a threat or a problematic case. Rougheri (2000: 417–419) traces a strong tendency in the Greek press to view Turks as the successors of ‘the Ottoman invaders’, ‘the barbarians of Anatolia’, ‘incorrigible Asian people which, for some 500 years, had given the worst example of a conquerer in the Balkans’ and ‘champions of slaughtering and persecuting people’. The Greek press also tends to depict Turks as imperialists who are ‘intransigent, unreliable, impudently provocative, aggressive, expansionist’, driven by both megalomania and inferiority complex and aiming to ‘restore the Ottoman Empire’ ( Rougheri, 2000: 419). Finally, the Greek press overwhelmingly claims that Turks are not Europeans. Since they identify Europeannes with ‘a synthesis of the Greek and the Roman spirit as well as that of Christianity’, Turks are automatically considered outside this category ( Rougheri, 2000: 419–420). Accordingly, ‘Greeks are by definition Europeans, while Turks are anything but Europeans...[T]hey can never be(come) Europeans, simply because they do not deserve the European identity’ ( Rougheri, 2000: 420). Rougheri (2000: 419) quotes a commentary which claims that Turks will never become European despite Kemalist reforms: ‘Even though Kemal tried to change them, to Europeanize them, the Turks remain deeply Turkish!... The West and Turkey are two different worlds’. 

Yanovski (2000: 310) finds two diametrically opposed tendencies in the Bulgarian press towards Turkey. Some depict Turkey as Islamic, backward, undemocratic, unstable, unpredictable, and hostile; others consider Turkey as secular, advanced, democratic, therefore predictable and friendly. Despite this duality, Turkey is never considered as truly European. Yanovski (2000: 319) quotes several Bulgarian newspapers which claim that Turkey is ‘a semi-European Muslim state’, located either ‘on the periphery of Europe or rather in Asia’, and thus it is ‘neither a classical Balkan state nor a European one’.

Finally, according to Buğday (2003) examining the representations of Turkey in the German magazine *Der Spiegel*, the image of Turkey in the 1990s was informed by political developments in Turkey. Especially, Turkish government’s heavy-handed policies towards Kurdish minority was depicted as Turkish ‘oppression’ against Kurds, and Turkey’s fight against the PKK terrorism was rather interpreted as ‘a struggle between peoples, Turks and Kurds’ ( Buğday, 2003: 304). In comparison to the Cold War period, there is a recurrent trend in *Der Spiegel* to reflect a critical approach to Turkey, during the post-Cold War period, in reaction to the controversial political developments in Turkey.

### Turkish representations of europe

The extant literature on the representation of Europe in Turkey compared the Cold War and the post-Cold War political contexts too. However, contrary to the European perceptions, Turkish perceptions did not considerably change after the Cold War. The overwhelming argument highlighted in the literature is that Turks considered Europe both as a source of inspiration and anxiety during the Cold War and this trend continued in the post-Cold War era. A notable difference between the two periods is the rising Eurosceptic reactions of Turkish political elite against the changing European attitudes towards Turkey after the Cold War.

***The Cold War representation of Europe in Turkey***. During the Cold War, the literature essentially offers two readings of Turkish approach to Europe: Europe as a model but also as a source of anxiety, and Europe as a threat. Similar to the Republican period, Turkish political elite during the Cold War idealized Europe as an ideal despite reservations against its imperialist nature. According to Stone (2002: 197–198), Turkish perceptions on Europe hardly changed much from the late Ottoman period onwards. Europe has always been considered as a ‘financial powerhouse’ and a ‘motor of modernity’ ( Stone, 2002: 198). However, what Stone (2002) overlooks is the anxieties Turks felt against Europe. Bilgin and Bilgiç (2012) offer a more elaborate perspective highlighting the ambivalence in the discourses of Turkish policymakers representing the EU/rope both as a source of inspiration and anxiety. According to Bilgin and Bilgiç (2012: 121), Turkish political elite considered EEC membership as a means to join the Western civilization. However, from mid-1970s onwards, Turkish policy makers tended to approach Europe not only as a source of inspiration but also as a source of anxiety, especially in response to the EEC’s ‘pro-Greek’ attitude towards the Cyprus problem and its favourable stance towards the Greek accession ( Bilgin & Bilgiç, 2012: 115). Similarly, Pesmazoglu (1997: 149) argues that while Turkey embraced Europe as a role model, European criticisms of human rights abuses in Turkey were dismissed as racist, and the Western criticisms of the Armenian issue and the Cyprus problem were charged with orientalism among Turkish intelligensia. Macmillan (2013: 142–143) traces a similar tendency among Turkish political elite who viewed Europe both as a model of civilization, and a threat to national sovereignty and culture.

Sezer (2010) joins the Turkish-European identity debate examining the works of two influential writers of twentieth-century Turkish literature, Ahmet Hamdi Tanpınar (1901–1962) and Peyami Safa (1899–1961). Safa’s post-1945 writings define two different Europes, ‘the old, purely mechanistic, positivistic and technological Europe as opposed to the new, more spiritually-oriented Europe which nevertheless suffers at the hands of an unstoppable spiritual crisis’ ( Sezer, 2010: 432). Tanpınar stresses the necessity of synthesizing ‘humanistic values of European modernity’ with ‘the artistic and literary narratives of Turkish culture’, unlike Safa, Tanpınar believes that such as combination help construct a ‘European Turkey’ ( Sezer, 2010: 435).

The prevailing scholarly literature emphasizes that Europe was also perceived in Turkey as a threat. As Nas (2001) indicates, 1970s led to the rise of Euroscepticism in Turkey which not only associated Europe with imperialism, but also believed that Turkey would never be accepted by Europeans due to its Ottoman/Islamic identity. Nas (2001: 184) quotes Cemil Meriç who drastically reflected this perception: ‘Even if we burn all the Korans, run down all mosques, we are Ottomans in the eye of the European; Ottoman meaning Islam. A dark, dangerous, hostile crowd’. Döşemeci (2013: 111) shares this analysis, arguing that the late 1960s marked the rise of a ‘new Occidentalism’ shared by both far right and far left segments of Turkish intelligensia in response to Turkey’s membership application to the EEC. Especially nationalist and Islamist journalists and academics dismissed the ‘Kemalist protect of Europeanization’ as a ‘diseased behavior’ stemming from an inferiority complex towards ‘the decadent, soulless, immoral, positivist and Masonic west’. Leftist organizations such as DİSK and chamber of mechanical engineers showed similar reactions claiming that Turkish and Western cultures had totally different foundations, and efforts to change Turkey would rather ‘dig the grave of Turkish civilization’ ( Döşemeci, 2013: 113).

Kuzmanovic (2008) points to the rise of pro-Islamic political actors under the leadership of Necmeddin Erbakan especially after the 1980s coup that persistently contested Kemalist Westernization through a civilizational discourse. Constructing Western-European civilization as ‘the Other’ of Turkey, these political actors depicted Kemalist attempts to ‘replace Islamic-Ottoman civilization with the Western’ as the main reason for the ‘ill-state of Turkish society’ ( Kuzmanovic, 2008: 47). Similarly, Helvacıoğlu (1999: 26–27) explains that the 1970s and the 1980s witnessed the rise of ‘Europhobic construction of Europe as a sinister force to divide the national unity’. For instance, Greece and France were viewed as two major sinister European powers working against Turkey’s interests ( Helvacıoğlu, 1999: 26).

***The Post-Cold War representation of Europe in Turkey***. Turkish perceptions towards Europe in the post-Cold War period did not necessarily change since Europe was both viewed as a path to follow and as a source of anxiety. Moreover, the European tendency to revisit their convictions about Turkey’s place in Europe after the Cold War further contributed to the escalation of Euroscepticism in Turkish politics. According to Kuniholm (2001: 27), in the 1990s, EU membership was considered in Turkey as a guarantee for Turkey’s continued Westernization and the evidence for its European identity. That said, Turkish political elite viewed European reservations for Turkish entry into the EU as a rejection of Turkey’s ‘civilizational commitment to Europe and the West’ (Ibid). In response, Turkish decision-makers used the ‘Islamist fundamentalism’ card to pressurize the EU to accept Turkey into Europe to prevent it from falling prey to political Islam (Ibid). Kuniholm (2001: 26) also claims that Europeans decided to extend customs union to Turkey to ‘preclude Islamists from coming to power in Turkey’.

Similarly, Nas (2001: 185) argues that Europe was increasingly viewed in Turkey as a guarantee of democracy and human rights in the 1980s, and a ‘safety valve’ against the threat of political Islam in the 1990s. However, the rearticulation of European identity after the Cold War led Europeans to question Turkey’s Europeanness especially regarding Turkey’s EU membership process (Ibid). This attracted Eurosceptic reactions from Turkish political elite.

Helvacıoğlu (1999: 27) argues, Turkish statesmen including the President Süleyman Demirel reacted to the political conditions issued for membership as indicators of European unwillingness to accept Turkey due to its Muslim identity. This attitude, according to Helvacıoğlu (1999: 27) indicates a duality among Turkish political elite towards Europe in the sense that they both blame Europe for treating Turkey as its other and exalt Europe as a primary model for Turkey’s Westernization. According to Helvacıoğlu (1999: 28), this duality reflected the ‘inferiority complex’ of Turkey towards Europe.

Güneş-Ayata (2003) points to the rising fear of division by foreign forces, i.e., the Sevres Syndrome, following the end of the Cold War shared by both nationalist right-wing and center left political forces in Turkey. It depicts Europe rather as an enemy with the objective of dividing Turkey. Güneş-Ayata (2003: 211–212) explains that introduction of the Copenhagen Criteria, the initiation of the accession process of the Republic of Cyprus, and the signing of the Customs Union between the EU and Turkey turned formerly pro-Western nationalist political forces in Turkey including the Nationalist Action Party (MHP) against Europe. The MHP dismissed Europe as an imperialistic power aiming for the ‘federalization of Turkey’ resulting into a national disintegration ( Güneş-Ayata, 2003: 212).

The Turkish Center left spearheaded by Bülent Ecevit’s Democratic Left Party (DSP) shares the reservations of Nationalist political actors. Ecevit blamed the EU as an imperialistic force aiming to divide Turkey with ‘ethnic and religious resurgences’, ‘impoverish the Turkish people’ and make Turkey dependent on European credits ( Güneş-Ayata, 2003: 214). Pro-Islamic political forces led by the Welfare Party (RP) made even more drastic depiction of Europe in the 1990s. Accordingly, the EU was shown a Christian club, established to prove that ‘the cross was superior to the crescent’ ( Güneş-Ayata, 2003: 216).

### The Focal Issues

***Civilization***. The focal issue ‘civilization’ resonates heavily during the period under scrutiny in which the extant literature emphasizes Turkey’s EU membership process as a decisive step towards the Republican ideals of reaching the level of contemporary civilizations. According to Döşemeci (2013: 47), the signature of the Ankara Association Agreement in 1963 was particularly considered by Turkish political elite as the realization of Atatürk’s ideal of reaching the level of contemporary civilization ‘by tightly binding Turkey to Western Europe and the highest standards of civilization that it represents’. Turkish press welcomed the signature as ‘the most productive and concrete step in Turkey’s 150-year effort to westernize and be considered an equal member of Western Civilization’ ( Döşemeci, 2013: 48). Some headlines even claimed that Turkey’s Europeanness was inescapably validated with the signature (Ibid). Similarly, Nas (2001:182) argues that the signing of the agreement led to the popular perception in Turkey that Turkey was considered as a European country. Moreover, it was viewed by Turkish politicians, including the Prime Minister İsmet İnönü, as a solid confirmation of Turkey’s inclusion in the Western civilization ( Nas, 2001: 183). Furthermore, Müftüler-Baç (1997: 18) claims that the Republican ideal to be incorporated into the West was translated into a desire for EU membership throughout the 1980s and 1990s.

The scholarly literature also points to the notion of ‘excessive Europeanization’ during this period highlighted by Turkish novelists such as Peyami Safa. Accordingly, Sezer traces, in Safa’s works of the 1950s, both an appreciation of Turkey’s European vocation and criticism of ‘excessive Europeanisation’ ( Sezer, 2010: 428). Safa believes that ‘Turkish-Islamic civilisation needs to be regarded as Occidental and European’, since the three fundamental pillars of the European civilisation, namely, Greece, Roman Empire and Christianity were built upon the Eastern Mediterranean, the Turkish homeland ( Sezer, 2010: 430). On the other hand, Safa also remains critical of too much Europeanization and prescripes a (Turkish) synthesis between Eastern spiritualism and Western materialism ( Sezer, 2010: 431).

Perhaps the most vivid emphasis on ‘civilization’ regarding the discussions on Turkey and Europe is the one offered by Huntington (1996) who defines Turkey as a torn country caught between Western and Eastern civilizations. Huntington (1996) claims that Atatürk made Turkey a torn country building a Western country on an Eastern society, and when Atatürk’s legacy began to erode, the Islamic revival in Turkey furthered the European perceptions on Turks as others. Huntington (1996: 146) claims that Turkey would never become an EU member not because of its failure to comply with membership conditionality but because of its ‘torn’ identity claiming to be Western but looking like Eastern. Huntington (1996: 146) refers to Özal who stated in 1992 that Turkey’s human rights record was a ‘made-up reason why Turkey should not join the EC. The real reason is that we are Muslim, and they are Christian, but they don’t say that’. Building upon Huntington, Bogdani (2010: 21) highlights that Turkey has ‘two souls’; the Western soul ‘confined to the cosmopolitan Istanbul elite and other metropolitan areas’, and the Eastern soul defined as backward, impoverished and ‘non-European’ stretching to the vast Asian interior. This split within Turkish identity largely dominated by the Islamic culture of the Eastern soul is why Europeans consider Turks as non-European (Ibid).

The literature particularly focuses on metaphors used by political elite such as Turkey as a bridge or gateway especially after the Cold War to prove that Turkey secured a unique position to nurture Western civilization. However, the extant literature remains highly critical of the ability of these metaphors to prove Turkey’s European or Western credentials. For instance, Atakuman (2010: 118) argues that the popular metaphors of the 1990s, defining Turkey as ‘a bridge between East and West’, ‘mosaic of cultures’ and ‘a cradle of civilizations’ reflected a similar tendency to formulate a unique identity for Turkey as both Western/European and Eastern/Anatolian. According to Huntington (1996: 149), the ‘bridge’ metaphor confirms Turkey’s torn identity by admitting the fact that Turkey connects East and West, ‘but is part of neither’. Similarly, Yanık (2009: 533) claims that metaphors used by Turkish statesmen to define Turkey such as ‘buffer’ and ‘bastion’ during the Cold War, and ‘bridge’ and ‘crossroads’ in the post-Cold War era, with the objective of gaining authority and legitimacy, rather emphasize Turkey’s ‘uniqeness’ and ‘exceptionalism’ than its Europeanness, therefore constantly contribute to, in a liminal way, ‘Turkey’s “othering” by Europe’. Especially, the terms such as ‘bridge between Europe and Asia’, ‘bridge between civilizations’, and ‘gateway to Europe’ aimed to justify Turkey’s EU membership ( Yanık, 2009: 540). However, the use of bridge metaphor reinforced Turkey’s liminal status and was embraced by European politicians who wished to emphasize Turkey’s non-European identity ( Yanık, 2009: 544).

Moreover, the signature of the Customs Union between the EU and Turkey in 1995 was introduced by Turkish political elite as the most important step for becoming European ( Alpan, 2015). Turkey regarded the Customs Union as a step towards full membership, although the EU never made such an indication ( Rumelili, 2007: 64). On the contrary, the Customs Union agreement triggered an intensive debate in the European Parliament revealing a dual construction of Turkey’s identity as different based on both inherent and acquired characteristics ( Rumelili, 2002: 176–182; Rumelili, 2007: 67). The opponents of the Customs Union agreement with Turkey voiced their concerns over Turkey’s desire to acquire European identity which, in their argument, would constitute a threat to the European identity ( Rumelili, 2002: 178). Therefore, they opposed the agreement since they viewed it as a step for Turkey to claim European identity ( Rumelili, 2002: 179). Supporters of the Customs Union both in Turkey and Europe emphasized Turkey’s stabilizing role as a ‘natural counterbalance’ to Islamic fundamentalism ( Rumelili, 2002: 181–184; Rumelili, 2008: 103). However, according to Rumelili (2008: 103), the justification of the Customs Union agreement on religious grounds is ‘self-defeating’, for it is based not on Turkey’s similarities, but ‘on the threatening possibility of its further differentiation’, and it also reproduces an understanding of Islam as potentially threatening to Europe.

Finally, the Luxembourg Council Summit, in which Turkey was denied candidacy, was highlighted by Buzan and Diez (1999: 46) who argue that ‘the old game is now over’ and the EU withdrew its membership perspective for Turkey essentially because Turkey and the EU have different interpretations of ‘Westernization’. Turkey’s resistence to comply with the political conditionality stems from a limited understanding of Westernization dating back to early 20th century. However, the EU’s understanding of Westernization is reflective of the 21st century with reference to more individual and social rights and liberties ( Buzan & Diez, 1999: 45). According to Müftüler-Baç (1997: 18), Turkey can reformulate its identity through political and democratic reforms establishing a congruence between these two interpretations of Westernization.

***Status in international society***. The existing scholarly literature indicates a strong tendency in Turkish political circles to connect Turkey’s EU membership process with Turkish aspiration to gain international legitimacy and avoid international isolation despite military coups that shattered democratic development in Turkey ( Atakuman, 2010; Bilgin & Bilgiç, 2012; Döşemeci, 2013; Yıldırım, 2014). Referring to the speeches of Turkish foreign ministers in last 1950s and early 1960s, including Fatin Rüştü Zorlu and Feridun Cemal Erkin, Döşemeci (2013: 27) claims that the main motivation behind Turkish quest for membership to European Economic Community was to be recognized as European and join the standard of civilization. Turkish journalists and academics in the 1960s shared the view that Turkey’s EEC membership would help reach ‘the level of Western Civilization and the standards of its prosperity’, while economic benefits would rather be of secondary importance ( Döşemeci, 2013: 38–39). The EEC membership was even praised as ‘the final stage of Turkey’s Westernization’ ( Döşemeci, 2013: 43).

Relying on EU archives, Yıldırım (2014) examines the first traces of Europeanism in Turkey following the Second World War. Accordingly, Yıldırım (2014: 115–119) depicts the establishment of the Turkish National Council of the European Movement in 1948 and the European Campaign of the Youth in 1951 with the participation of both major political parties, the CHP and the DP, as highly indicative of Turkish aspiration for recognition as a legitimate European state. However, Yıldırım (2014: 121) claims that the 6–7 September 1955 events triggered by the burgeoning Cyprus problem considerably decreased the Turkish enthusiasm for pan-European activities and resulted in the closing down of the the European Campaign of the Youth.

The scholarly works particularly highlight the persistent efforts of Turkish intelligentsia to prove Turkey’s Europeanness through membership in Western organizations such as NATO and participation to European events such as friendly football matches with European countries ( Oğuzlu, 2002; Şenyuva & Tunç, 2015). Oğuzlu (2002: 583) argues that siding with European powers in issues of international politics and joining European institutions such as NATO and the EU were considered as the most ‘cost-efficient’ ways of encouraging Europeans to recognize Turkeys European identity during the Cold War. However, according to Oğuzlu (2002: 583), Turks did not necessarily choose to be an integral part of European identity. Rather than fully embracing ‘a domestic reform process aimed at internalizing the constitutive norms of the European international society’, Turkish decision-makers deemed it sufficient to participate in the European state system through membership in intergovernmental ogranizations such as NATO.

Şenyuva and Tunç (2015: 574) argue that from early 1950s onwards, Turkey attached particular importance to friendly football matches with Western European teams not only because Europeans were the best teams to play against, but those matches confirmed Turkey’s Europeanness as well: ‘As Turkey was European, it was only natural that Turkey played with‘other’ Europeans’. Şenyuva and Tunç (2015: 575) explain that the debate over Turkey’s Europeanness was reflected into its standing in Football as early as the 1950s. Turkey was invited to the inaugural conference of UEFA in Vienna in 1955 that enabled its participation in all European competitions. However, FIFA objected by keeping Turkey in Asian qualifiers for the World Cup and the Olympics until 1962 resulting into the ‘Asia–Europe Question’ of Turkish football (Ibid). Turkish authorities viewed this as ‘an issue of identity and involved themselves in a relentless effort to prove Turkey’s Europeanness in football’ ( Şenyuva & Tunç, 2015: 577).

Bilgin and Bilgiç (2012: 114) explain that the Ankara Association agreement in 1963 was essentially considered by Turkish political elite as ‘a next step in the Republican project of Westernization’ and a new victory for ‘Turkey’s efforts to become a European state’. It was also viewed as a decisive step to avoid diplomatic isolation for Turkey, considering the European reactions after the 1960 coup ( Bilgin & Bilgiç, 2012: 115). Similarly, the additional protocol with the EEC in 1970 was welcomed as an important stage for the fulfilment of Turkey’s quest for becoming ‘a member of Europe and as a member of the free world’ ( Bilgin & Bilgiç, 2012: 115). Moreover, behind the official application of Turkey for membership in 1987 and the signature of the Customs Union in 1995, a common Turkish motivation, according to Bilgin and Bilgiç (2012: 116–117) was to avoid diplomatic isolation in international society and to be recognized as a legitimate member of the Western civilization.

Throughout the 1980s the Turkish government worked heavily for Anatolian historical sites to be included in the World Heritage List of UNESCO, a list dominated by European cultural history. According to Atakuman (2010), this essentially evidences Turkish quest for being recognized as European. Especially, the inclusion of the Hittite capital of Hattusha to the list in 1986 was praised by Turkish authorities as a sign that proved Turkey’s credentials as ‘a civilized European state’, since Hittites were one of the earliest known Indo-European-speaking populations in history ( Atakuman, 2010: 116).

After the Cold War, the loss of Turkey’s strategic value also damaged its quest for being recognized as Western and European. In response, Turkish government used a civilizational discourse to prove its European credentials in the early 1990s. Atakuman (2010: 118) refers to Turkish President Turgut Özal who claimed that Europeans cannot deny Turkey’s Europeanness since Turkish homeland is where Europe was born:

You yourselves (Europeans) accept that your own civilization originated in Mesopotamia (where civilization flowered for the first time), then Anatolia, the Aegean basin, and Rome. We have at least as much right as you to adopt these ancient civilizations as our own, since they are those of our own land.

On the other hand, the literature also indicates a tendency in Europe to view Turkey as a security engine consolidating Europe’s status against the Soviet threat during the Cold War ( Aybet & Müftüler-Bac, 2000; Çapan & Onursal, 2007; Challand, 2009; Keyder, 2006; Nas, 2001; Oğuzlu, 2002; Özoğuz, 2007). As security concerns outweighted identity considerations, European countries accepted Turkish military strength as a crucial factor for the preservation of European security. Therefore, European pragmatism to incorporate Turkey into the European security community stemmed from the urge toprovide European security against Soviet expansionism ( Aybet & Müftüler-Bac, 2000; Çapan & Onursal, 2007; Krieger, 2006; Nas, 2001; Oğuzlu, 2002). The post-Cold war period, however, negated the existing security arrangements and Turkey’s aspiration for joining the EU was increasingly viewed as a threat to the international status of Europe since Turkey was dismissed as non-European ( Aybet & Müftüler-Bac, 2000; Diez, 2004; Oğuzlu, 2002).

***Nationalism***. Regarding the focal issue ‘nationalism’, contrary to the previous period which emphasized the construction of a pro-European nationalism through Republican reforms, the third period witnessed the rise of anti-European nationalism in Turkey in the forms of Pan-Turkism, Pan-Islamism and Eurasianism. The extant literature emphasizes the rise of pan-Turkist and pan-Islamic tendencies as key indicators of Turkish nationalism during and after the Cold War. Aktürk (2007) claims that Turkish official stance from 1950s onwards has never been pan-European but rather ‘pan-Turkist’ referring to the Turkish policy of officially recognizing ‘Turkic’ states right after the collapse of the Soviet Union. It was also ‘pan-Islamist’ because of the return of Arabic call to prayer during the DP government in 1950 and the foreign policy of the RP of Necmeddin Erbakan to form alliance with Islamic countries in mid-1990s. Similarly, Robins (1996: 72–73) indicates that mid-1980s and early 1990s witnessed ‘the rise of Islamic fundamentalism and the reawakening of Turkic expansionism’. Turkey’s renewed interests in the Middle East and the newly independent states of Turkic origin in the Central Asia, according to Robins (1996: 73), confirm the thesis of ‘the return of the repressed’. Moreover, violent attacks against non-Muslim Turkish citizens during 6–7 September 1955 are particularly emphasized in the literature as a turning point where radical nationalism flourished in Turkey through ‘the socio-economic, ideological and political transformations of the Democrat Party era’ ( Kuyucu, 2005: 361). Furthermore, Heper (2004) emphasizes the intensification of Turkish nationalism especially in response to the rising Kurdish separatism in 1980s.

The post-Cold War Turkish politics was particularly emphasized with the emergence of Kemalist Eurasianism that blamed pro-Western policies for the decline of ‘the secular and social Turkish nation-state’ ( Akçalı & Perinçek, 2009: 551). Aiming for ‘the creation of a non-Western “Eurasian space” through an alliance with Turkey’s eastern neighbours like Russia and Iran and even with Pakistan, India and China’, Kemalist Eurasianism particularly stood against Western indifference to the PKK terrorism and its lukewarm approach to the Cyprus problem ( Akçalı & Perinçek, 2009: 559–560).

The existing literature also highlights Turkish communities as a source of rising nationalism in Europe. Turkish minorities became a source of local tensions and xenophobia in Greece and Bulgaria especially in the 1980s and 1990s ( Anagnostou, 2001; Yanovski, 2000). Moreover, the presence of Turkish migrant workers from 1960s onwards triggered ultra nationalist reactions by neo-Nazi groups against immigrant Turks as well as other ethnic groups ( Kühne, 2000; Mandel, 1995; Pratt-Ewing, 2003). For instance, ‘Terror, murder, and Turk hunting’ became the main motto of such violent groups in Germany ( Mandel, 1995: 269). In response, Turkish youth formed their own violent gangs with ultra nationalist rhetoric (Ibid).

***State-citizen relations***. The prevailing literature emphasizes “state-society relations” both within the Turkish and European contexts. State-society relations in Turkey are examined with reference to the rise of political Islam and the issue of Kurdish insurgency. In Europe, the focus is rather on how Europeans reacted to Turkey’s heavy-handed policies towards Kurds and human rights abuses, and how Turkish migrant workers are viewed in Europe.

Regarding the Turkish context, scholars point to a burgeoning identity crisis in Turkey exacerbated by the rise of different religious and ethnic groups. Helvacıoğlu (1996) points to the Islamic revival in Turkey after the Democrat Party came to power in 1950. It was consolidated after the 1980 coup emphasizing ‘Islamic religiosity’ as an integral part of Turkey’s political identity. Accordingly, both self-identifed Turkish-Kemalist nationalists such as Kenan Evren and Sunni-Islamicists such as Süleyman Demirel and Turgut Özal used Islam as a political tool to mobilize masses ( Helvacıoğlu, 1996: 510). Müftüler-Baç (2000: 31) argues that the post-Cold War era especially contributed to the deepening of Turkey’s identity crisis dividing Turkey into Westernist and Islamist camps. European ambivalence towards Turkey exacerbated its identity crisis playing into the hands of pro-Islamic actors in the 1990s (Ibid). Keyman (2006: 210) emphasizes the co-existence of transformation and crisis in the realms of politics, economics and culture largely stimulated by the revival of political Islam, rising popular demands for Kurdish rights and the fight against Kurdish insurgency. Helvacıoğlu (1996: 503) claims that Euopean statesmen such as the President of France, Jacques Chirac supported Turkey’s EU membership with the motivation to prevent political Islam from taking over Turkey. Keyder (1998) argues that modernization in Turkey took a non-Western turn in the 1990s. While modernization had been equated to Westernization since the proclamation of the Turkish Republic, the 1990s witnessed the popular rise of ‘non-Western modernization’ that associated ‘Islam’ with ‘modern’ ( Keyder, 1998: 40). Similarly, Gülalp (1998) points to the rise of Islamist discourse along with the decline of Westernist and Europeanist discourse in the 1990s. The rising popularity of the pro-Islamic Welfare Party indicated a non-Western/European turn in Turkish political discourses., According to Gülalp (1998: 64), pro-Islamic discourses associated Europe with relentless capitalism and imperialism, and prescribed Islam as the right way for Turkish modernity. Conversely, Özbudun and Keyman (2002), and Buğra (1999) argue that the Islamic challenge brought success to the Islamic-oriented parties in national and municipal elections; and successful Islamic-oriented economic actors, whose increasing presence in economic life proved that Islam can co-exist with free market capitalism, globalization and modernity.

Keyder (2006: 76) claims that after the Cold War, opposition groups including the Islamic and Kurdish movements along with the Leftist intelligentsia resorted to pro-European rhetoric to dismantle the Kemalist establishment through EU membership process. In response, the EU’s political conditionality especially regarding the rights of Kurdish minority and Islamist groups was viewed by the Kemalist elite as a threat to the 'unitary state’ and 'homogeneous nation' characteristics of Turkey ( Oğuzlu, 2002: 588). Therefore, EU membership, resulting in the violation of Turkey’s unitary character, would be highly undesirable for Turkey’s Kemalists (Ibid).

The literature also analyzes state-society relations within European context. Scholarly works emphasize Europeans view on the Kurdish issue and human rights abuses in Turkey, and how Turkish migrant workers are treated in Europe. The 1980 *coup* and human rights violations perpetrated by the military government particularly drew the attention of European politics, press and literature. The military government was often dismissed as despotic and barbarian ( Atakuman, 2010; Aydın, 2003; Challand, 2009; Helvacıoğlu, 1996; Soenen, 2000).

In the post-Cold War period, the image of Turkey was marked by Turkish ‘oppression’ against Kurds, and fight against PKK was represented in *Der Spiegel* as ‘a struggle between peoples, Turks and Kurds’ ( Buğday, 2003: 304). Similarly, Turkish government was condemned by the European parliament for its repressive approach to Kurdish minority ( Levin, 2011). The mistreatment of the MPs from the pro-Kurdish Democracy Party (DEP) led to the suspension of the Customs Union negotiations. Talks resumed only after Turkey complied with the European Parliament’s call for reforms to secure fundamental human rights and pluralist democracy ( Rumelili, 2002: 116–167). In response, Turkish political elite charged the EU with ‘Western hypocracy’, as the EU was claimed to have turned a blind eye to the atrocities in Bosnia ( Rumelili, 2002: 169–170).

European approaches to Turkish migrants living in Europe have also been an important point of reference in the extant scholarly literature. Having a closer look into European experiences with Turkish migrant workers in Germany, Straube (2000) scrutinizes the stereotypes against Turks in German society which directly resonate with the image of Turkey in Germany. Straube (2000: 144) argues that German images of Turks changed remarkably after the arrival of Turkish immigrats in 1961, as ‘[t]he emerging common living space led, on both sides, to myths, anecdotes and stereotypes with regard to the other’. The terms Auslander (foreigner), Kameltreiber (Camel-rider) and Kümmeltürke (cumin-Turk) were increasingly used to define Turks, while immigrant Turks countered with Schweinehirt (swineherd) to define Germans, starting from 1960s (Ibid). Conducting a small-scale survey to 15 German university students in 1992, Straube (2000: 145) finds that German students perceived Turkey as ‘an Oriental country that is opening itself to Western culture’. Turkish man was viewed as ‘sitting in teashops, sun-tanned, Muslim’ and Turkish women as ‘suppressed, disadvantaged with regard to men, subject to their men, an appendage of the man’, ‘veiled’ and ‘not allowed to speak their minds’ (Ibid). According to students, Germans regarded Turks as ‘smelling garlic’, ‘with large families and no money, looking for work or needing help here, unwanted Gastarbeiter who take away jobs’, ‘workers who we do not need’ and bringers of the ‘creeping danger of Islam’ ( Straube, 2000: 145).

Göbenli (2003: 287–289) explains that Germans had welcomed Turkish workers who were expected to do the dirty work for them and leave in a couple of years. However, xenophobic and racist attitudes against Turks increased after the realization that they would live in Germany indefinitely. The image of Turks is conditioned by stereotypes towards immigrant Turks. Accordingly, a Turkish man is depicted as ‘a “backward” Anatolian peasant, carrying a big moustache, walking two meters in front of his wife, a Muslim of strong belief’ ( Göbenli, 2003: 293). Similarly, a Turkish woman is perceived as ‘suppressed and dependent on their husbands, wearing headscarf, walking behind their husbands’ ( Göbenli, 2003: 294).

Finally, Mastnak (2000: 247) summarizes the Austrian perception of Turks as ‘unskilled workers who do “dirty work” which is extremely necessary but not valued at all’. Mastnak (2000: 248) argues that the Austrian stereotype of the historical Turkish invaders was supplemented with a new stereotype associated with backwardness.

## The Fourth Period (1999–2016)

The final period under scrutiny covers the most recent developments regarding Turkey and Europe starting from the Helsinki European Council Summit (11–12 December 1999), when the EU candidacy status was recognized for Turkey, up until mid 2010s. During this short period of time, numerous political and cultural developments took place triggering identity debates between Turkey and Europe including the start of accession negotiations between Turkey and the EU (3 November 2005), the Nobel Prize won by Turkish novelist Orhan Pamuk (12 October 2006), French President Nicholas Sarkozy's speech against Turkey’s EU accession in the 2007 election campaign (21 February 2007), and the joint agreement on the status of refugees reached by the EU and Turkey (18 March 2016).

The prevailing literature in the post-1999 period is overwhelmingly informed by politics and international relations discipline, while historical studies, in comparison to other periods, are almost non-existent. In this period, interdisciplinary studies combining two or more genres such as history, sociology and politics also proved to be a very popular. Sociology remains a highly studied field during this period, while a new discipline, communication is particularly highlighted as an emerging field of study. Finally, the period, although to a lesser extent, is also informed by other genres including psychology, sports, linguistics, education, geopolitics and fine arts presenting a multidisciplinary approach to the study of identity and culture in Turkey and Europe.

In terms of methodology, the recurring gap in the extant literature persists in the final period since there is still an evident lack of a comprehensive discourse analysis with reference to political texts. Nevertheless, the works of Çağatay-Tekin ( 2008; 2010); Levin (2011), Buckingham (2013) and Aydın-Düzgit ( 2012; 2013; 2015; 2016) remain as notable exceptions since they offer critical discourse analysis examining political texts and speeches regarding the European discussions of Turkey’s Europeanness. Their analyses hence bring a methodological novelty to the literature indicating the necessity of a comprehensive research on the topic. This project will build upon these works by not only examining the European representations of Turkey, but also the Turkish representations of Europe interactively through a critical discourse analysis.

Empirically, in comparison to other periods, the literature extensively covers the identity representations of Turkey and Europe primarily in line with Turkey’s EU accession. That said, European representations of Turkey is studied more than Turkish representations of Europe, while, similar to other periods, the number of studies examining both representations in a comparative fashion is rather limited.

### European representations of Turkey

This period witnesses the intensification of debates on Turkey’s ‘Europeanness’. Turkey’s EU accession constituted a crucial setting for the articulation of Turkey’s differences from Europe ( Aydın-Düzgit, 2013; Dixon, 2010; Rumeli & Çakmaklı, 2011; Walter & Albert, 2009). The extant literature reflects two main tendencies in this period. First, it primarily emphasizes the othering of Turkey on cultural grounds. Accordingly, opponents of Turkey’s EU accession often base their contestations on Turkey’s non-Europeanness with a particular reference to its distinct culture, religion, history, geography and demography ( Brewin, 2003; Grigoriadis, 2006; Kuzmanovic, 2008; Yılmaz, 2007). Therefore, the incompatibility of values between Turkey and Europe has been indicated as a key rupture point negating Turkey’s capability for EU membership. Studies focusing on European public opinion and press highlight a strong tendency to associate Turkey with Islam and hence a source of threat to Europe. Also, resentments towards Turkish immigrants contribute to the othering of Turkey.

Second, studies emphasize both inclusive and exclusive discourses on Turkey which judge Turkey’s European credentials with reference to European ‘values’ and European ‘culture’, respectively. The literature particularly highlights Turkey’s liminal status in Europe which contributes to the competing European discourses on Turkey’s Europeanness ( Rumelili, 2004; Rumelili, 2007; Rumelili, 2011). Finally, a minority of studies reflects a rather optimistic tendency, especially in British press, to depict Turkey as a ‘positive other’.

Studies scrutinizing European contestations against Turkish EU membership overwhelmingly trace a common tendency to denote Turkey’s ‘otherness’. According to Diez (2007), below is how Turkey’s difference from Europe is articulated to justify its unfitness for EU accession:

The predominance of different values is distributed on a geographical basis; such value differences matter to communities, so they are worth preserving; and the difference between Turkey and the present EU member states is sufficiently greater than that among member states, so it is justifiable to treat Turkey differently and to deny membership.

According to Yılmaz (2007), religion is viewed as a crucial identifier of Europeanness and used as a strategy of othering Turkey. Since Turks do not share the Christian tradition, they are automatically considered outside the ‘genetic pool’ of Europeanness ( Yılmaz, 2007: 299). Similarly, Çağatay-Tekin (2008: 757) finds that the discourse in France against Turkish EU entry shows European/French identity (Self) and Turkish identity (Other) as antithetical. French political discourses define the Turkish other not only ‘different (or strange), but also aggressive, dangerous, threatening, and most importantly, inferior to the [European] Self’ (Ibid).

Strasser (2008: 186) indicates that ‘Turks/Kurds/Muslims/Orientals’ are viewed as Europe’s ‘distinctive others and Austrian political elite emphasize the necessity of protecting ‘occidental culture’ from ‘a Turkish siege’ by preventing Turkish accession. Matonyte and Morkevicius (2009: 971) find that European political elite perceives Turkey as a bigger threat than Russia since it is viewed as ‘destabilising the convenient European ways of thinking ‘from within’ and calling for a revision of European boundaries and values’.

Aydın-Düzgit (2013: 16) suggests that Turkey is not necessarily considered by the European Commission as a ‘threatening other’. However, the European Commission’s discourse is more geared towards presenting Turkey as a threatening other to Europe when it comes to Turkey’s EU accession (Ibid). Similarly, Suvarierol and Aydın-Düzgit (2011: 165) indicate that despite having a cosmopolitan agenda evidenced through its institutional motto of ‘unity in diversity’, the Commission’s depiction of Cosmopolitan Europe ‘stop[s] at the borders of Turkey’. The Commission officials resort to stereotypical depictions of Turkey based on culture and identity (Ibid).

Studies examining opinion polls in Europe trace a similar tendency. European public stands against Turkish accession on the grounds that its accession would ‘endanger’ the Europeanness of Europe ( Canan-Sokullu, 2011; Ruiz-Jiménez & Torreblanca, 2007; de Vreese *et al.*, 2008). The European representations of Turkey as ‘other’ have also been reflected into the everyday lives of Europeans whose perceptions on Turkey are primarily informed by the historical image of Turks, European interactions with Turkish migrants and the human rights abuses in Turkey ( Yılmaz, 2005: 28).

The representation of Turkey as Europe’s other is not only used by the opponents of Turkish EU entry. Buckingham (2013) traces the representation of Turkish EU accession in Spanish press between 1999 and 2010 and finds that despite Spanish support for Turkish EU entry, Turkey is still represented as Europe’s cultural ‘Other’. Boria (2006: 503) reaches a similar conclusion for the Italian case. Both opponents and proponents of Turkish EU accession in Italy resort to stereotypes that depict Turks as ‘others’. Similarly, Negrine (2008), Negrine *et al.* (2008) and Wimmel (2009) emphasize that contrary to German and French press, Turkey’s religious, cultural and demographic difference was not considered by British press as a cause for rejecting its EU accession, but Turkey is still represented as ‘being more ‘different’ than ‘similar’ from Europe’.

The post-9/11 perceptions in Europe against Islam directly resonate with contestations against Turkey ( Casanova, 2006; Challand, 2009; Göle, 2006; Jones, 2013; Ramm, 2009). Bunzl (2005: 505) highlights the European concerns over Turkey’s EU accession as ‘by far the most crucial aspect of Islamophobia’. Turkey’s EU membership aspirations set in motion a series of anti-Turkish political campaigns in several EU member states where far right political parties gained electoral success. The argument that ‘Islam is external and even antithetical to the culture of the EU’ has been increasingly defended by right wing political actors in Europe to reject Turkey’s membership ( Özyürek, 2005: 509).

Minkenberg (2012) explains that churches in EU member states became the epicenter of anti-Turkish propaganda. Public resentment towards Turkey’s EU accession is primarily informed by anti-Islamic sentiments ( Kentmen, 2008). There is an established fear within European public that ‘Islamisation of Europe’ will most likely ‘come about through Turkey’s EU membership’ ( Canan-Sokullu, 2011: 484). The rejection of the European Constitution was essentially based on the public fear against Islam/Turkey in the Netherlands and France ( Benhabib & Isiksel, 2006; Göle, 2006). Similarly, perceptions towards Turkey within Italian public are primarily influenced by the representations in Italian press that equalize Turkey with Islam ( Marcellini & Şenyuva, 2011).

Studies focusing on political discourses in Europe indicate a tendency among European politicians to highlight ‘the so-called Judeo-Christian foundations of Europe’ and the distinct cultural and religious features of Turks to invalidate Turkey’s EU membership efforts ( Grigoriadis, 2006; Kuzmanovic, 2008; Levin, 2011; Macmillan, 2013; Rumelili, 2007). Even the proponents of Turkish EU entry tend to associate Turks with Islam and thus consider Turkey ‘alien to Europe’ as evidenced ‘in their calls for a ‘coexistence’ of Europeans and Muslims’ ( König *et al.*, 2006: 151).

Similar to the previous period, European representations of Turkey in the post-1999 era are also influenced by interactions between Europeans and Turkish migrants. Political and social discourses against Turkey’s EU membership have been based on resentments towards Turkish migrants in Europe ( Kubicek, 2004; McLaren, 2007). In Western European countries, Turkish immigrants are still perceived as foreigners ( Spruyt & Elchardus, 2012). The problems associated with the integration of Turkish immigrants are shown as crucial indicators of Turkey’s lack of capability to be integrated into Europe ( Brewin, 2003). For instance, patriarchal lifestyle of immigrant Turks that victimizes women through forced marriages and domestic violence is viewed in Austrian society as evidence of Turkey’s ineligibility for EU membership ( Strasser, 2008: 187). Moreover, far right populist political actors in Western Europe justify their opposing stance against Turkish EU accession through the ‘othering’ of immigrant Turks ( Öner, 2014).

On the other hand, debates in Europe regarding Turkey’s EU membership primarily reveal ‘Europe’s own confusion and hesitancy about its own identity’ ( Müftüler-Baç & Taşkın, 2007; Wood, 2013). There is a duality among Europeans about whether to judge Turkey’s membership with reference to its compatibility with ‘thick’ European ‘culture’ or its compliance with ‘thin’ European ‘values’ ( Aydın-Düzgit, 2011; Benhabib & Isiksel, 2006; Dostál *et al.*, 2011; Jones, 2013; Kirişci, 2008; Le Gloannec, 2006; Özoğuz, 2007). Opponents of Turkey’s EU membership essentially raise the question of ‘culture’. They claim that although Turkey complies with membership conditionality, it will never belong to Europe ‘which bear[s] the stamp of Christianity and humanism’ ( Rumeli & Çakmaklı, 2011).

Kylstad (2010) claims that the EU's self-identification as a ‘Christian-culturalist’ union can be evidenced through its discourses on Turkey’s bid for membership. The discourses of the European People’s Party (EPP) members inf the European Parliament reflect a similar tendency to define ‘European culture’ with reference to (Christian) religion. They claim that the religion-induced European culture ‘constitutes the essential component of a consolidated democracy’ ( Aydın-Düzgit, 2015: 163). A similar discourse is also spelled out by German Christian Democrats who claim that ‘democracy is unthinkable without Christian values’ ( Kösebalaban, 2007: 102). Turkey is hence represented as ‘a diluter of a culturally homogenous Europe’ ( Aydın-Düzgit, 2015: 170).

Shakman-Hurd (2006: 402), on the other hand, points to the fact that opponents of Turkish EU entry are not only composed of religious/Christian Europeans but also of seculars as well. Turkey’s EU membership bid ‘brings up long dormant dilemmas internal to Europe regarding how religion and politics relate to each other’ (Ibid). Turkey’s EU candidacy poses a challenge to the established notion of the ‘Judeo-Christian’ secularism in Europe forcing Europeans to revisit their own definition of secularism (Ibid). In contrast, proponents of Turkish EU entry emphasize European ‘values’ which are universal and can be adopted by outsiders, rather than ‘culture’ which is particularistic and cannot be acquired by non-Europeans ( Rumeli & Çakmaklı, 2011). Turkey’s cultural difference does not preclude its integration into Europe provided that it internalizes European norms and values ( Scherpereel, 2010). Turkey, hence, proves to be a major scene for contestation between universalism and particularism in Europe ( Aydın-Düzgit, 2012; Baban & Keyman, 2008; Diez, 2007; Levin, 2011; Macmillan, 2013; Risse, 2010; Rumeli & Çakmaklı, 2011).

Debates about Turkey’s ‘Europeanness’ have a particular reference to Turkey’s liminal, ‘a partly-self, partly-other’ position ( Rumelili, 2008). Turkey is often represented as a country of contradictions, geographically located between the East and the West, and with a Muslim society under a secular Kemalist regime ( Rumelili, 2008: 102). Challand (2009)’s emphasis on the ‘centaur’ metaphor used in Geman cartoons to represent Turkey proves to be an intruiging illustration of Turkey’s liminality. The depiction of Turkey as half-human (Western) and half-animal (Eastern) reflects its dual nature caught between the East and the West ( Challand, 2009: 76). Turkish society is primarily viewed as divided into Eastern and Western Turks ( Brewin, 2003: 138). Therefore, ‘Turkey is neither seen as a stable European ‘Other’ nor as a European ‘Self’, but rather a ‘thing on the (European) doorstep’ ( Walter & Albert, 2009).

Turkey’s liminality contributes to the competing discourses in Europe. Discourses focusing on the exclusive aspect of European identity consider Turkey inherently non-European due to its distinct geography and culture, thus unfit for membership. Others reflect optimisim for Turkey’s potential to become European provided that it ‘develops economic and political institutions in line with European values and standards’ ( Rumelili, 2004: 44). Furthermore, Turkey’s liminal position resonates strongly with the discussions on ‘‘defining the frontiers’ and ‘defending the borders’ of Europe’ ( Rumelili, 2007: 77). Debates in the European Parliament and the European Commission about Turkey’s EU membership often lead to another debate about where the borders of Europe end and where Europe should be heading ( Aydın-Düzgit, 2013; Çapan & Onursal, 2007; Keyder, 2006; Kastoryano, 2006; Levin, 2011; Malatesta & Squarcina, 2011). On the other hand, for some scholars, Turkey’s liminal or in-between position presents an opportunity for the EU to prove its normative power ( Diez, 2005; Nicolaidis, 2004). The EU can both exert influence upon Turkey by triggering a political transformation through membership conditionality and clarify Europe’s identity by emphasizing its difference from Turkey ( Diez, 2005: 633).

Finally, Paksoy and Negrine (2016: 500) emphasize that Turkey is represented as a ‘positive other’ in British media. Turkey is not only depicted as ‘other’ in conventional terms, but also considered to have a potential to function as a bridge betwen the East and the West assisting Europe to reach out to the Muslim world (Ibid). This argument holds similarities with, the ‘bridge metaphor’ and ‘Alliance of Civilizations’ to increase dialogue between the East and the West. It also negates the claims about British press’ tendency to ‘portray Turkey in connection with persistent exclusivist perceptions’, similar to the portrayals made by press in continental Europe ( Devran, 2007; Paksoy, 2013; Schneeberger, 2009). On the other hand, Phillips (2013) also claims that British press emphasizes European ‘values’ in the discussion of Turkish EU entry. It focuses on the necessity of preserving ‘secularism’ as a crucial requirement for EU accession ‘as a response to the rise of the Islamically-identified Justice and Development Party (AKP)’ (Ibid).

### Turkish Representations of Europe

In the post-1999 period, Europe as a source of inspiration and anxiety continued in Turkish representations with an increasing reference to Turkey’s EU membership bid ( Bilgin & Bilgiç, 2012). Republican quest for Westernization has been associated with EU accession. Therefore, Europe is viewed as ‘a choice of political modernization – a source of pride for the whole nation’ and Turkish desire to become an EU member stems from its desire to have a place in the ‘family photo’ of Europe ( Kastoryano, 2006). From 1999 onwards, Turkish political elite, both government and opposition, perceived ‘Europe’ (and the EU in particular) as everybody’s ‘project’, a common cause ( Alpan, 2014: 74). The Copenhagen Criteria outlining the requirements for EU accession was adopted by Turkey as a guideline for its Europeanization and Turkish political elite was adamant to comply with these criteria regardless of whether compliance would result in EU accession or not. Top government officials called them ‘Ankara criteria’ to denote their significance for Turkey’s advancement evidencing the established Turkish representation of Europe as a source of inspiration for Turks ( Dağı, 2006; Tocci, 2005). Europe remained a ‘promised land’ and ‘the embodiment of a harmony between the local and the global’ in the AKP government’s discourses until the late 2000s ( Alpan, 2016: 19). Moreover, there is a remarkable tendency among Turkish intellectuals and statesmen to highlight a ‘desirable’ or ‘ideal’ Europe focusing on what Europe ‘must’ be and how Europe ‘must’ behave. Europe is considered to be based on universal values and norms including democracy and human rights, instead of rigid cultural, religious and historic qualities ( Arkan, 2016; Aydın-Düzgit, 2006; Balkır & Eylemer, 2016; Gülmez, 2008; Müftüler-Baç, 2005; Oğuzlu & Kibaroğlu, 2009; Öniş, 2010). Similarly, ‘Europeanness’ is largely associated with ‘democracy’, ‘democratic consolidation’ and ‘reform’ ( Alpan, 2015). Europe is also viewed as having a potential to become a post-national polity dependent upon Europeans’ ability to accept Turkey as an equal partner ( Aydın & Keyman, 2004; Baban & Keyman, 2008).

However, Europe is also considered as a source of anxiety or even threat generating eurosceptic reactions from Turks. Turkish political elite develop ‘anti-EU’ sentiments in reaction to the EU’s representation of Turkey as ‘non-European’ ( Arkan, 2016; Bilgin, 2004; Güneş-Ayata, 2003; Öner, 2011). . The accession of former Communist countries was considered ‘a blow to Turkey’s self-image as a highly valued ally, staunch defender of the West, and bulwark against communism’ ( Rumford, 2008: 121). Turkish political reactions to the EU’s ‘unfair’ application of its membership conditionality towards Turkey are embodied in their representation of Europe as a violator of universal principles ( Aydın-Düzgit, 2006; Balkır & Eylemer, 2016; Celep, 2011; Gülmez, 2008; Hortaçsu & Cem-Ersoy, 2005; Öniş, 2010). Besides, Turkey often criticized the construction of European identity through Christian exclusivism ( Morozov & Rumelili, 2012). This period indicates an increasing tendency among Turkish political elite to represent the EU as a Christian club ( Arkan, 2016; Bilgin & Bilgiç, 2012; Gülmez, 2013; Vaner, 2003). Intensively used after the 1997 Luxembourg Summit, the term was revived by Turkish statesmen in the post-1999 period. It was used to ‘reproduce’ the argument that ‘if the EU declined to grant membership to Turkey, it would be doing so for cultural and religious reasons’ despite all the efforts of Turkey ( Rumelili, 2008: 104). It was also argued that Europe would not be ‘completely’ European unless Turkey was admitted to the EU ( Arkan, 2016: 142).

Turkish representation of Europe as a source of threat not only developed as a reaction to controversial European discourses, but also secures deeper societal roots. Turkish contestations against Europe resonate strongly with the ‘Sèvres Syndrome’. It refers to ‘fear of plots by external enemies, especially the Western countries, and their alleged internal collaborators – ethnic and religious minorities in Turkey– to weaken, carve up and terminate the existence of the Turkish Republic’ ( Nefes, 2013: 252). It is a ‘siege paranoia’ that delegitimizes the West and fuels mistrust against Western states ( Guida, 2008). It reflects an image of ‘a conspirational West bent on the destruction of Turkish national integrity with the collaboration of ‘internal enemies’’ ( Kösebalaban, 2002: 131). It dictates Turks not to ‘enter into economic, political and cultural pacts and alliances with the Western world’ ( Yılmaz, 2006: 12). The Treaty of Sèvres is depicted by some scholars as the most important symbol of Turkish distrust towards Europe ( Bardakçı, 2010; Macmillan, 2013). The Sèvres Syndrome hence stands as a common feature of Turkish political culture since it is not only embraced by Kemalists, but also pro-Islamists and Nationalists ( Guida, 2008; Gülmez, 2014; Macmillan, 2013).

Especially, the EU’s membership conditionality is contested by both left and right wing political actors in Turkey as the re-introduction of the Sèvres Treaty to divide Turkey ( Avcı, 2011; Başkan-Canyaş & Gümrükçü, 2015; Buhari-Gülmez, 2017; Gülmez, 2014; Uslu, 2008; Yılmaz, 2011). The EU is blamed for maintaing a hidden agenda to damage the integrity of Turkish state through political reforms including minority rights, the abolution of capital punishment and liberalization of foreign land ownership ( Alpan, 2014; Buhari-Gülmez, 2017; Celep, 2011; Gülmez, 2008). The representation of Europe in Turkey both as a model and a threat is also reflected into the perceptions of Turkish society exemplified in football fans who both secure a desire to be accepted and be a part of Europe, and wish to seek revenge on Europe ( Bora & Şenyuva, 2011).

Turkish representations of ‘Europe’ tend to fluctuate in tandem with Turkish assessments of the justice or injustice of ‘European’ treatment of Turks. This brings selectivity in the representation of Europe focusing on ‘negative representations of the country by European actors whilst ignoring neutral or positive views’ ( Fisher Onar, 2009; Fisher Onar & Evin, 2010: 298). Fisher Onar and Evin (2010: 308–309) argue that Turkish perceptions towards Europe proved to be more conciliatory following the AKP’s electoral victory in 2002. Not only pro-Islamic and secular segments but also nationalist political actors in Turkey toned down their criticisms against Europe and sought for a convergence with the EU accession criteria (Ibid). However, the EU-phoria in Turkish politics was curtailed following the introduction of EU-led reforms contested by Kemalist and nationalist political actors as a threat to the integrity of the Turkish Republic ( Avcı, 2011; Aydın-Düzgit, 2006; Celep, 2011; Gülmez, 2008). Besides, the *Leyla Şahin* case at the ECtHR upholding a secularist ban on veiling in public institutions swiftly led to the disillusionment of the AKP government with Europe. According to AKP officials, this decision proved that ‘‘EU-niversal’ values cannot encompass the ‘alternative’ reading of such values put forth by figures within and/or sympathetic to the pro-religious camp’ ( Fisher Onar & Müftüler Baç, 2011: 379). Similaly, pro-Islamic intellectuals in Turkey reacted to the Court decision by reverting to ‘their traditional estimation of ‘Europe’ as a ‘totalitarian’ project ontologically incapable of acknowledging the truths of ‘Others’’ ( Fisher Onar & Evin, 2010: 309). Especially after Ahmet Davutoğlu became Turkish foreign minister in 2009, the Turkish representation of Europe in official discourses began to change. The AKP government’s discourse framed ‘Europe as inferior and as belonging to a different civilisation, thus revealing a more self-confident, inclusive and Islamist national identity discourse’ ( Macmillan, 2013). A primary reason is argued to stem from the AKP goverment’s objective to replace the Orientalist image of the Turk as ‘sick man of Europe’ with that of ‘the Robust Man of Europe’ and turn the crisis-ridden Europe into a ‘stronger, richer and safer’ Union ( Macmillan, 2013: 114).

Finally, along with the disappearance of EU membership prospects and the reversal of the EU-led reform process in Turkey, the literature has recently focused on the ‘de-Europeanization’ of Turkey. The term refers to Europe’s decreasing salience in Turkish discourses and actions indicative of a ‘growing scepticism and indifference in Turkish society towards the EU/Europe’ ( Aydın-Düzgit, 2016; Aydın-Düzgit & Kaliber, 2016; Boşnak, 2016; Kaliber, 2016; Onursal-Beşgül, 2016; Saatçioğlu, 2016; Yılmaz, 2016a; Yılmaz, 2016b). Turkey’s de-Europeanization indicates that Europe is now represented as a ‘resented guardian’, an ‘unwanted intruder’, a ‘discriminatory entity’ and even ‘democratically/economically inferior’ to Turkey ( Alpan, 2016; Aydın-Düzgit, 2016).

### The Focal Issues

***Civilization***. This period resonates strongly with the term ‘civilization’ as both Europe and Turkey dived into heated debates on how to place Turkey into a certain civilization. While the goal of ‘reaching the level of contemporary civilizations’ remained in the Turkish agenda, concepts such as ‘Alliance of civilizations’ have been increasingly discussed to denote Turkey’s place in civilizational debates. European discourses on Turkish EU entry have increasingly been informed by a civilizational rhetoric that serves to either include or exclude Turkey. The Turkish question triggered intensive debates in Europe regarding the nature of European civilization and the prospects for its evolution through multiculturalism and post-westernism.

Turkey’s EU membership bid especially led to debates in Europe of whether Europe could be defined as a monolithic civilization exclusive to certain groups. As pointed out above, there is a lack of clarity among Europeans over whether they consider the EU as an exclusive civilizational project with cultural, historical and religious referents or as an inclusive community based on norms and values ( Özoğuz, 2007). According to Kastoryano (2006), Turkey’s EU candidacy has served as a ‘mirror’ for Europe underscoring the paradoxes of its expectations and values involving ‘universality’ formulated in terms of human rights and ‘particularism’ embodied in ‘Christian’ religion. Especially, the AKP government’s pursuit of EU membership through a rigorous reform process ‘challenged the construction of Islam and Europe as incompatible identities both in Europe and in Turkey’ ( Rumelili, 2008: 108).

According to Delanty (2003), the Turkish case has brought a major challenge to the meaning of Europe as a ‘geopolitical, social and cultural space’. It forced the EU to move beyond postnationality to a transnational encounter with multiple civilizational forms. Hence, the Turkish EU accession debate stands as test case for the possibility of a post-western, a polynational, polyethnic and multicentric Europe not based on the fiction of a ‘European people’ or a territorial domain ( Delanty, 2003: 20). Building upon Delanty, Rumford (2008: 119) claims that it would be more accurate to view Turkey as one agent of Europe’s postwesternization that will help Europe abandon the dichotomy of self and other in the re-definition of its identity. Parker (2009: 1098) claims that a cosmopolitan outlook to the ongoing debate on Turkey’s EU membership will help transcend the ‘dogmatic perceptions of inside /outside’ and can be ‘constitutive of more peaceful relations within and between Turkish and European societies’.

The discourse of ‘civilization’ is widely used by both opponents and proponents of Turkey’s EU accession ( Kuzmanovic, 2008). The opponents highlight the distinct character of Turkey that renders it unfit for EU membership ( Baban & Keyman, 2008: 115). Culturalist approaches to Europe emphasized by the opponents of Turkey’s EU entry primarily adopt a definiton of monolithic civilizations separating Europeans and Turks ( Ramm, 2009). Turkey is not viewed as part of Europe ‘because of its different civilizational roots’ not based on ‘Christianity and Enlightenment values’, namely, the common features of European civilization ( Kösebalaban, 2007: 101). There is a ‘civilizational clash’ between Islam and Europe raising the question of whether ‘Islamic civilization was ever well disposed towards quintessentially ‘European’ values’ including freedom of thought, tolerance, religious pluralism, and gender equality ( Taras, 2013: 80). Hence, the EU membership of Turkey was equalled by European political elite with the end of Europe ( Brewin, 2003; Kösebalaban, 2007). The endorsement of such as a rhetoric which emphasizes ‘civilizational divide’ in its approach to Turkey left Turks to feel like ‘scorned suitors’ ( Keyder, 2006: 73). Besides, according to some scholars, adopting a ‘fortress Europe’ mentality to deny Turkey on civilizational grounds might ‘risk aggravating the prospects of a ‘clash of civilizations’’ ( Kirişci, 2008: 20).

The proponents of Turkey’s EU accession too refer to the term ‘civilization’ in their discourses. Especially, centre-left parties in Western Europe emphasize Turkey’s membership as an indicator of Europe’s ‘civilizing mission’ ( Keyder, 2006: 79). Pro-Turkish European political elite also consider Turkey’s EU accession process as evidence that Europe ‘is multicultural, secular, but multi-faith, peaceful and open to the rest of the world’ ( Rumelili, 2008: 105). Therefore, the ‘Clash of Civilizations’ discourse has been used by both opponents and proponents of Turkey’s EU entry to either discredit its European credentials or support its role in remedying the civilizational clash ( Öner, 2009). Dixon (2008) investigates to what extent the thesis of the Clash of Civilizations is valid regarding the Turkish case and finds that Turkey and Europe/West do not necessarily represent distinct civilizations. Turkish people support democracy and human rights similar to Europeans except for more support for religious and authoritarian rule and less tolerance towards minorities in Turkey (Ibid).

Civilizational rhetoric is also widely used by Turkish political elite to emphasize Turkey’s critical role for Europe. Turkish political elite traditionally presents EU accession as the finalization of Turkey’s Westernization in line with the Republican ideals ( Nas, 2001). Oğuzlu (2011) however argues that Turkey’s Westernism in recent years has rather been ‘Turkey-centric’ especially in its approach to the EU. He claims that Turks embrace European values and institutions ‘based on their national interests rather than their national identity’ (Ibid). Moreover, Bilgin and Bilgiç (2012: 119) claim that the AKP government adopted a rather different civilizational discourse than its predecesor, the DSP. While the DSP government sought to find a common civilizational ground with Europe emphasizing shared values and history, the AKP’s discourses ‘wrote Turkey out of EU/ropean civilization and sought commonality in the practice of dialogue of civilizations’ (Ibid). Hence, there is a shift in civilizational discourses between Turkish governments since 1999 from representing Turkey as Western to ‘belonging to and offering oneself as the interlocutor of Islamic civilization’ ( Bilgin & Bilgiç, 2012: 121). In this respect, the ‘Alliance of Civilizations’ project was pioneered by the AKP government to establish a bridge between the East and the West, but by doing so, the Turkish government also confirmed Turkey’s liminal position. According to Yanık (2011),

Turkey’s liminality, as discussed above in detail, is ‘neither created by the EU alone nor regarded by the Turks as a bad thing’. Turkish political elite contributed to the creation of such an identity for Turkey and ‘carr[ied] liminality as a badge of honor’ ( Yanık, 2011: 82). Turkish exceptionalism in the post-Cold War period was constructed via liminal representations of the country as a mediator/peacemaker between East and West ( Yanık, 2011: 80). The metaphors popularly used by Turkish politicians such as ‘bridge,’ ‘door,’ ‘latch and key,’ ‘crossroads,’ and ‘gate’ feed into Turkey’s liminal identity and help Turkey formulate a unique position for its foreign policy ( Yanık, 2009; Yanık, 2011). Rising Eurasianism in Turkish politics is also linked to the aspirations to emphasize Turkey’s liminal position as the ‘center’ or ‘hub’ of Eurasia ( Yanık, 2009). Similarly, relying on the neo-Ottomanism discourse, the AKP government declared Turkey as a ‘central’ country in world politics ( Davutoğlu, 2008; Yanık, 2011). Besides, Turkey’s ‘liminality’ reinforced through the continuous use of ‘bridge’ metaphor places Turkey in a less classifiable category than the regular ‘othering’ practices ( Yanık, 2009). However, the AKP’s neo-Ottomanist ideology feeds into the clash of civilizations thesis since it draws a fine line between the East and the West by totalizing civilizational and cultural identities such as ‘Western’ and ‘Christian’, and ‘Islamic’ and ‘Eastern’ ( Ertuğrul, 2012: 184). According to Ertuğrul (2012: 184), it also confirms ‘the otherness of Turkey in European imagery’ by placing Turkey on a different category of civilizations.

***Status in international society***. The literature discusses the potential impact of Turkey’s EU membership on the international status of both Turkey and Europe. In the Turkish mind, Europe stands as a high-status superordinate group, and, in trying to join the EU, Turkey confirms that it is already a low status group aspiring to elevate its status through the EU ( Hortaçsu & Cem-Ersoy, 2005: 110). Besides, according to Nas (2001; 187), EU accession represents a break away from the ‘psychological isolation’ of Turkey stemming from its liminal position. Bilgin and Bilgiç (2012) argue that in the post-1999 period Turkey viewed Europe ‘as a source of the risk of diplomatic isolation vis-à-vis the International Society’ especially given the fact that Greeece, a historical rival of Turkey, remained an EU member. Therefore, Turkish statesmen did not consider remaining outside EU/rope as a viable option for Turkish foreign interests ( Bilgin & Bilgiç, 2012: 119). Conversely, Yanýk ( 2009; 2011) claims that Turkish political elite did not necessarily try to disprove Turkey’s liminality but actually promoted it to raise Turkey’s status and credibility in international society. According to Kuzmanovic (2008: 42), Turkey’s unique identity with a predominantly Muslim society and a secular political system not only proves that Western and Muslim civilizations can be coexist, but also grants Turkey the privileged international position of a broker between civilizations. Moreover, neo-Ottomanism ^3^ was designed by the incumbent AKP government to elevate Turkey’s status to a ‘central country’ in its neghbourhood and the spokesperson of the Islamic world ( Arkan, 2016; Davutoğlu, 2008; Ertuğrul, 2012; Yanık, 2011). By ‘re-representing’ the Islamic civilization in world politics, the neo-Ottomanist discourse expected to provide the ‘confidence and vigor’ for the AKP leadership's bid for regional hegemony and global impact ( Ertuğrul, 2012: 183).

Turkey’s liminal position is advertised by Turkish statesmen not as a threat but as an asset to Europe ( Morozov & Rumelili, 2012). Accordingly, Turkey’s EU membership will not only increase Turkey’s international status, but also raise the credibility of Europe as a ‘multicultural’ actor ( Alpan, 2015). Besides, Turkey’s EU membership is directly linked to Europe’s capability to achieve the ‘Alliance of Civilizations’ ( Alpan, 2016: 19). Therefore, the emphasis on Turkey’s hybridity challenges ‘the binary interpretations of European identity, which rest upon the construction of Europe/Asia, West/Islam as mutually exclusive and inherently incompatible identities’ ( Morozov & Rumelili, 2012: 41). On the other hand, standing against Turkey on religious grounds might risk undermining Europe’s credibility in the Muslim world ( Kirişci, 2008: 21). On the European side too, proponents of Turkish EU entry highlight that Turkish accession will raise the international status of Europe proving that ‘the European project is not culturally sealed but allows Europe to bridge the gap between the West and Muslim countries’ ( Baban & Keyman 2008: 112). Turkish accession might help the EU become a global power and a viable alternative to the US-led world order ( Baban & Keyman, 2008; Keyder, 2006). Therefore, admitting Turkey to the EU would prevent Europe from becoming increasingly isolated, culturally closed, and irrelevant in the global state of affairs ( Baban & Keyman, 2008: 112). Conversely, opponents of Turkish accession claim that Turkish membership would weaken the EU’s place in world politics, while some even believe that Turkish accession would be the end of Europe ( Aybet, 2006; Baban & Keyman, 2008; Kuzmanovic, 2008; Yılmaz, 2007).

On a final note, cultural events including the Eurovision song contest and European football tournaments are highlighted in the literature as important triggers of debates both in Turkey and Europe about Turkey’s Europeanness ( Alpan & Şenyuva, 2015; Christensen & Christensen, 2008; Şenyuva & Tunç, 2015). For instance, Turkey’s iconic victory in the 2003 Eurovision song contest turned out to be rewarding for Turkey’s European credentials ( Christensen & Christensen, 2008). Turkish victory was particularly welcomed in British media shifting the long standing ‘sick man of Europe’ narrative to the ‘slick man of Europe’ ( Christensen & Christensen, 2008: 169).

***Nationalism***. This period also witnessed the rise of nationalist discourses in Turkey as well as Europe. During the AKP era, Kemalist nationalism has been renamed as ‘Ulusalcılık’ charged with ‘uncompromising anti-Westernism; externalization of Islam from Turkish nationalism; and ethnic exclusionism’ ( Uslu, 2008). It is different from traditional nationalism (milliyetçilik) especially in the sense that it ‘has hailed secularism and emphasized the Turkish rather than Islamic dimension of national identity ( Yılmaz, 2011). It is generally viewed as a loose anti-Western alliance between the Turkish ultra-left and ultra-right around the protection of Kemalist principles ( Erşen, 2013). This new neo-nationalist trend is also defined as ‘defensive or inward-oriented’ nationalism with the objective of preserving the unitary and secular character of Turkey against the ill effects of globalization and Europeanization ( Öniş, 2007). The Sèvres Syndrome resonates strongly with this new nationalism. Associations with this neo-nationalist leaning sued Nobel Prize winner Orhan Pamuk, well-known writer Elif Şafak and late Armenian intellectual Hrant Dink for insulting Turkishness ( Taşkın, 2008).

The ‘Ulusalcı’ foreign policy dictates closer ties with Eurasia rather than the West since the West does not consider Turkey as a legitimate member of the Western international community ( Oğuzlu, 2010: 670). Besides, Eurasianism is further embraced by Kemalist intellectuals and politicians as a tool to criticize the West/Europe ( Akçalı & Perinçek, 2009; Erşen, 2013). Therefore, the pro-European nationalist understanding of the Kemalist establishment has evolved into a rather anti-European discourse. The sharp reversal in Kemalist nationalism stems primarily from Turkey’s EU accession process in which the EU empowered the AKP, a pro-Islamic government. Political and social reforms enacted for EU accession such as minority rights and foreign land ownership were alleged to damage the integrity of the Turkish Republic ( Börzel & Soyaltın, 2012; Celep, 2011; Yılmaz, 2011). The AKP was claimed to be giving unacceptable concessions to Europe for the sake of EU membership ( Gülmez, 2008; Taşkın, 2008).

On the other hand, the AKP government promotes a different kind of nationalism with an Islamic flavor through the introduction of ‘neo-Ottomanism’. The neo-Ottomanist discourse is used by the AKP elite to construct an exceptionalist Turkish identity which is ‘cosmopolitan’ and ‘multicultural’ ( Yanık, 2011: 84). Focusing on the ‘forgotton’ and ‘overlooked’ legacy and ‘unique’ geopolitical position of Turkey, neo-Ottomanism claims to offer a more active role for Turkey in its neighbourhood ( Arkan, 2016: 141). In this respect, textbooks revised in Turkey following the EU reform process continued to inject the AKP-led nationalism drawing the boundaries between ‘us’ and ‘them’ based on ethnicity (Turkishness) and religion (Islam) ( Çayır, 2009: 50). Although expected to dismiss nationalist discourse and adopt an inclusive rhetoric thanks to the EU accession process, the new textbooks rather ‘promote a notion of solidarity among the Turkic-Islamic population while paying no attention to non-Turkish and non-Muslim groups’ ( Çayır, 2009: 51).

However, the EU accession process produced contestations against Turkish national identity by both pro-EU actors in Turkey and pro-Turkey actors in Europe. They emphasized the necessity of transcending the primordial understanding of nation through increased social and political rights and liberties for Kurdish, Armenian and other minority groups ( Johansson-Nogues & Jonasson, 2011; Macmillan, 2013). Turkey’s Europeanization process also contributed to the projection of Kurdish nationalism into legitimate political platforms through political parties since Turkey’s EU accession process facilitated the recognition and expression of Kurdish identity ( Saylan, 2012; Tezcür, 2010).

Finally, the literature has recently concentrated on the rising far-right populism and ultra-nationalism across Europe in conjunction with the Eurozone crisis, the refugee crisis, rising Islamophobia and anti-Turkish sentiments ( Lazaridis *et al.*, 2016; Wodak *et al.*, 2013; Wodak & Boukala, 2015). The 2014 elections in the European Parliament were a particular success for eurosceptic politicians feeding on the rising nationalism across Europe in response to the economic crisis and intensification of immigrant problems ( Brack, 2015; Grabbe & Groot, 2014; Mudde, 2014). Moreover, the increasing popularity of far-right nationalist political actors including Marine Le Pen in France, Nigel Farage in Britain and Norbert Hofer in Austria indicates that nationalism and euroscepticism in Western Europe have been moving from the periphery to the political mainstream ( Akkerman *et al.*, 2016; Brack & Startin, 2015; Eger & Valdez, 2015; Vieten & Poynting, 2016). Besides, the British referendum to exit the EU and the electoral victory of Trump in the US indicate that nationalist populism is becoming a truly ‘Western’ phenomenon transcending the borders of the European continent ( Inglehart & Norris, 2016).

***State-Citizen relations***. Finally, this period dwells on state-society relations concentrating on minority rights and social rights including the headscarf ban and the criminalization of adultery as crucial indicators of interactions between Turkey and Europe. Turkey’s Europeanization process in the final period particularly enabled ethnic minority groups to gain more social and political rights ( Grigoriadis, 2008; Kirişci, 2011; Nas, 2012; Öniş & Yılmaz, 2009; Oran, 2007; Toktaş & Aras, 2009; Yılmaz, 2012; Yılmaz, 2014; Yılmaz & Soyaltın, 2014). The EU-led reforms however revealed a split within Turkish society indicating their tolerance to cultural and political freedoms. Nationalists in Turkey viewed those reforms as a threat to national sovereignty and the security of the state. Others attaching priority to the development of democracy and human rights regarded the EU as a guarantor of these values ( Avcı, 2011; Narbone & Tocci, 2007; Nas, 2001; Öniş, 2003; Yılmaz, 2011). Especially, Kemalist political elite perceived the EU-led reform process as a threat to the integrity of the Republic and drew further away from Europe and Turkey’s EU accession process ( Alpan, 2014; Celep, 2011; Dikici-Bilgin, 2017; Erşen, 2013; Gülmez, 2008; Oğuzlu, 2002). On the other hand, the EU accession process did not necessarily provide an enabling environment for Alevis, another ethnic minority group, to gain more social and political rights, and this has been raised as a crucial example of contradiction, if not discrimination, on the side of the EU ( Çarkoğlu & Bilgili, 2011; Özdalga, 2008).

Moreover, other hot topics including the headscarf ban at Turkish universities and the AKP government’s initiative to criminalize adultery were under the radar of the extant literature. Fisher Onar and Müftüler Baç (2011) focus on the ways in which Turkish women's role in society are viewed as a measure of Turkey's ‘Europeanness’ examining the adultery debate in Turkey and the *Leyla Şahin* verdict of the ECtHR. While secular Turks view women rights as an indicator of Turkey’s Europeanization, Islamists think otherwise (Ibid). They rather see the increased role of Islam in Turkish politics as a natural result of the adoption of the European values in Turkey ( Fisher Onar & Müftüler Baç, 2011: 388). Accordingly, the court decision was viewed by governmental circles as a breach of European values and a direct reflection of Islamophobia in Europe ( Fisher Onar & Müftüler Baç, 2011: 386). The court decision is considered as a turning point where the AKP lost eagerness for EU accession and succumbed to authoritarianism ( Baudner, 2014; Kubicek, 2014).

In Europe, the reform process was praised as a significant progress for Turkey’s Europeanization ( Börzel & Soyaltın, 2012; Kaliber, 2013; Keyman & Aydın-Düzgit, 2007; Kubicek, 2011; İçduygu, 2011; Müftüler-Baç, 2005; Öner, 2014; Tocci, 2005). However, the government’s efforts to criminalize adultery under the guise of EU reform faced with harsh criticisms and was highlighted by Turkey-sceptic European politicians as evidence of Turkey’s lack of European credentials ( Wood & Quasier, 2005).

Finally, the extant literature emphasizes the identity crisis of Turkish migrants in Europe focusing on how they struggle to be part of European society despite rising xenophobia and racism in Europe, and why some deny assimilation and emphasize Turkish identity even though holding a European passport, while others embrace European identity and deny Turkishness ( Ehrkamp, 2006; Kaya, 2011; Kaya & Kentel, 2005; Küçükcan, 2008; Küçükcan & Güngör, 2009; Özselçuk, 2014; Sarıaslan, 2016).

## Conclusion

This analytical review has located several gaps in the existing literature on Turkey-Europe identity relations. First, there are very few, if any, longue-duree studies that trace the evolution of identity relations between Turkey and Europe. There is an evident lack of studies with a comprehensive focus on the topic covering all four periods under scrutiny. The second relevant pattern is the relatively uneven distribution of the existing literature across different historical periods. Around 150 of the 313 works analyzed focuses on the identity relationship between Turkey and the EU in the contemporary post-1999 period, while in the 1789–1922 period, the number of relevant works is 40, in the 1923–1945 period 45, and in the 1946–1988 period, it is 65. Thirdly, most of the existing literature presents either the European perceptions or the Turkish perceptions in a particular historical period without analyzing the interaction between the two. In other words, scant attention has been paid to the ways in which European representations of Turkey’s identity resonate in Turkey and in turn shape Turkish representations of European identity. By paying equal attention to both European and Turkish representations of the Turkey-Europe identity relationship in all four historical periods, this review is therefore a major contribution to the existing literature.

Moreover, this analytical review has identified some gaps in the study of identity relations within specific periods. The European perceptions of different moments of Ottoman modernization in the first period has not been adequately studied, and there is a clear need to avoid reifying Europe and distinguishing between different policies and attitudes of each major European power, particularly England, France and Germany. From the Ottoman side, the main lacuna is methodologically informed studies examining the difference and interaction between the elite and public perceptions throughout the first period. Regarding the second period, the literature lacks studies focusing on how the Republican reforms and its (oppressive) actions towards public affected the Turkish image in Europe. Similarly, the third period requires more focus on how European attitudes towards Turkish immigrants contributed to the European image in Turkey.

Merging the arguments raised in the vast pool of scholarly sources, this analytical review has reached the following conclusions. The Nineteenth century was crucial in the formation of “Europe” and “the Turks” as both sides tried faced each other in battle, diplomacy, and culture, and in this Russia competed with the Ottoman Empire as her nemesis. Regarding the European representations of Turkey, the historical image of Turks as “the other of Europe” did not necessarily change in the second period despite Republican reforms to prove the contrary. In the third period, the literature highlights the European political pragmatism towards Turkey during the Cold War in which security concerns overshadowed identity discussions. European public opinion however remained unchanged especially given the interactions with Turkish migrants. On the other hand, the post-Cold War resurfaced identity debates and reinforced the image of Turkey as non-European again.

In the final period, the European representations vastly concentrated on the othering of Turkey on cultural grounds. Especially, resentments against Islam and migrants fed into contestations against Turkey’s EU membership. Moreover, the final period witnessed the intensification of competing discourses on Turkey, both inclusive and exclusive with reference to European ‘values’ vs European ‘culture’. Especially, Turkey’s liminality contributes to the competing discourses depicting Turks as both the negative and positive other of Europe.

Regarding the Turkish representations of Europe, the second period reveals the Turkish tendency to view Europe both a model and a threat. Intriguingly, Turkish nationalism in this period was largely deemed as pro-European. The duality in Turkish perceptions continued in the third period framing Europe both as a source of inspiration and anxiety. Unlike European representations, the Cold War did not change or conceal Turkish views towards Europe. Besides, the literature emphasizes the rising Turkish Euroscepticism in response to the changing European attitudes towards Turkey after the Cold War. Similar representations remained unscathed in the final period. On one hand, Europe was viewed as everybody’s ‘project’, ‘promised land’ and ‘ideal’. It was also dismissed as violator of universal principles and Christian club responsible for the revitalization of Sèvres Syndrome in Turkish society. Towards the end of the final period, literature also stresses an increasing pace of de-Europeanization in Turkish politics in response to the loss of EU membership perspective.

## Ethics and consent

Ethical approval and consent were not required.

## Data Availability

No data are associated with this article.

## Footnotes

^1^ An intergovernmental project foreseeing a political and economic partnership among European powers based on the French idea of harnessing Germany into a European framework to prevent its revival through Anschluss. For more detail, see Barlas and Güvenç (2009: 428).

^2^There is a tendency in the literature to either prefer the word ‘West’ or use ‘Europe’ and ‘West’ interchangeably. The former could be argued to indicate the Republican tendency to attach greater value to the notion of an all-encompassing West from a geographical perspective, while the latter reveals a tendency to equate Europe with the West taking into account the technological, cultural and political advancements throughout history.

^3^ defined as a discourse that highlights Turkey’s Ottoman past and mixes it with geographical uniqueness to justify an active foreign policy in Turkey’s neighborhood.
