# Supplementary material for: Europe and Turkey: identities in evolution. An analytical literature review
Source: Open Res Eur. 2023 Jul 26;3:120. [Version 1] doi: 10.12688/openreseurope.16176.1 (PMC10445910; doi:10.12688/openreseurope.16176.1)
Supplement: Peer review history

# Reviewer report: Reviewer response for version 1

**DOI:** 10.21956/openreseurope.17463.r34122

**Published:** 2023-12-14

**Authors:** Paul T. Levin

**Affiliations:** 1 Institute for Turkish Studies, Department of Economic History and International Relations, Stockholm University, Stockholm, Stockholm County, Sweden

This is a very comprehensive survey of the existing research on the question of identity in the mutual perceptions of Turks and Europeans over a long period of history. The review is divided into four periods, the first starting with the final century of the Ottoman Empire and the last ending with the Turkey-EU refugee deal in 2016. With this comprehensive approach, the authors hope to fill a lacunae in the literature: there are few studies of the mutual representation between these two actors over the *long durée*. They express the hope that their review can provide the basis for an "evolutionary analysis of mutual identity representations between the two sides over more than a two-hundred-year period." (p. 3)

 The authors structure the examination of the literature in each period by first presenting a historical context, after which they turn to European representations of the Ottoman Empire, and then to Ottoman representations of Europe. They conclude the examination of each period by discussing four focal issues: nationalism, civilization, status in international society, and state-citizen relations. These four issues are no doubt interesting and important, and the discussion of them is informative, but there is no prior discussion that explains the theoretical or practical significance of these issues, or why these and not others were chosen. A slightly stronger theoretical framework for the review could have enhanced the coherence of the review.

 The review is long and very rich in information. It is a gold mine for anyone interested in a birds-eye view of the mutual representation of identity and otherness between Ottomans/Turks and (non-Turkish) Europeans. This review should be a natural starting point for students and scholars writing on this topic in the future, and the concluding identification of eight major gaps in the literature should point the way to future research. The review is easily accessible and written in a straight forward manner by scholars who clearly know the topic. The focus on the mutuality of representations is one of the strong points of the study, as much of the literature (generally speaking) has tended to focus on European representations of Turkey and Turks. The periodization is reasonable and there were many revelations for me when reading.

 On the critical side, the authors could have discussed the Turkey-Europe dichotomy that they assume in a more reflective manner. Does the use of this dichotomy reify the construct that Turkey is not part of/partly Europe? The review is, as has been noted above, short on theory. A fuller theoretical discussion of in particular the four focal issues could have been useful. The reading of the research is generally very good, but the ambition to cover a vast topic means that the readings of the literature is at times somewhat shallow. There are minor but consistent grammatical errors (in particular when it comes to definite/indefinite articles) that marginally detract from the presentation.

 When it comes to limitations, it is not clear why the project begins with the French Revolution and not sooner. There were, of course, interactions between the Ottoman Empire and Christian kingdoms in Europe prior to that date. The review could have benefited from a motivation for this choice of limitation. Having said that, the period covered is already vast and extending it further could have made it unwieldy. Another limitation is that only works in two languages--English and Turkish--are included. Europe is of course much bigger than just the UK and while English has become the *lingua franca* of academia, the exclusion of sources in eg: German and French might have had consequences for the findings. On the other hand, just including Turkish sources is a significant improvement of the existing scholarship. A recommendation for future scholarship that wants to build on this review would be to add sources in other languages.

Are all factual statements correct and adequately supported by citations?

Yes

Is the review written in accessible language?

Yes

Are the conclusions drawn appropriate in the context of the current research literature?

Yes

Is the topic of the review discussed comprehensively in the context of the current literature?

Yes

Reviewer Expertise:

Turkish studies. European representations of Turkey. Collective identity.

I confirm that I have read this submission and believe that I have an appropriate level of expertise to confirm that it is of an acceptable scientific standard.

# Reviewer report: Reviewer response for version 1

**DOI:** 10.21956/openreseurope.17463.r34382

**Published:** 2023-09-11

**Authors:** Alaaddin F. Paksoy

**Affiliations:** 1 Anadolu University, Eskişehir, Turkey

**INTRODUCTION**

 Aiming to analyse a period of more than 200 years is a challenging but original attempt to unveil the bigger picture of Turkish-European relations within the specific dimension of identity. The research sample of this article is based on three periods, all of which are important for a better understanding of Türkiye's relations with Europe. The periods do not have similar characteristics. The first period is by far the longest. It contains so many controversial and historical events that even historians find it difficult to understand. The second and third periods are more manageable, although the literature on these years is probably more time-consuming. The article is interested in four focal points that are useful to explain the historical and contemporary aspects of Türkiye-EU relations within the framework of the notion of "identity".

 This article is instructive. The bibliography gathered in the research includes 313 works, which makes the study powerful and unique in its field. The article not only presents the overall picture in the literature, but also presents important but little known details that help to analyse what the article focuses on. For example, Kaiser Wilhelm's trip to Istanbul and Jerusalem and his spiritual experience on that trip make the article more interesting to read. Another stimulating and instructive detail is the establishment of the "Turkish National Council of the European Movement" and the "European Youth Campaign" and the contribution of the two leading political parties, the CHP and the DP, to them.

 One of the original features of this study is its comprehensiveness. The literature review does not only include research from political science, international relations, sociology or media studies. Some examples presented in the article originate from representations of Türkiye's European or non-European identity in arts and literature. Even a section focuses on the history of education in Türkiye, discussing textbooks from the Republican period. Another originality of this work is the scope of the literature. Most studies on Türkiye-EU relations use data from the most powerful EU countries, such as Germany, France and the UK (before Brexit), and sometimes Italy and Türkiye's neighbour Greece. However, this study also refers to the research related to the reverberations of Türkiye's European identity in less popular European countries in Türkiye-EU relations such as Norway, Czech Republic, Slovakia, Romania and Bulgaria.

**SOME CRITIQUES**

 The article summarises the literature that points to the inadequacy of Türkiye's Western image, despite the huge modernisation efforts in the Republican period. One criticism can be made of the article's approach to the 'top-down' nature of modernisation in the Republican period. It is clear that the modernisation movement in the Republican period was led by the Turkish elite, and in most cases it was a top-down social change. However, the article's constant emphasis on the notions of ‘top-down’ and ‘rapid modernisation’ inadvertently ignores the success of Türkiye's transformation during the Republican period. Although most transformations during the Republican period were carried out with a top-down approach, most reforms were actually internalised by state institutions and society in the long run. There is not much room for negotiation on most of these reforms in Türkiye today.

 The article shows that the anti-EU stance in Türkiye has not only been taken up by nationalists or Islamists, but also seriously by secular Kemalists and anti-capitalist politicians such as members of the Turkish Social Democrats (Bulent Ecevit's DSP party). The article gives several examples of how suspicious Kemalists and nationalists are of Türkiye’s EU bid. However, the article does not sufficiently explain the literature that shows why religious conservatives (or Islamists, according to some people) or mainly AK Party members had a very positive approach to Türkiye-EU relations and successfully led probably the most vibrant period of Türkiye-EU relations between 2002 and 2006. This point is crucial because those years had a huge impact on the development of the AK Party in the following years and shaped Türkiye's last two decades.

 Another missing point in the article is the view of European politicians on Türkiye's European or non-European identity. Political history is shaped by various actors and institutions. Politicians play a major role in this shaping. There are several examples of the views and actions of Turkish politicians (e.g. Özal, Demirel and Ecevit) in the article. However, the views of European politicians were not sufficiently represented.

 The article refers to Gummer (2010) and Vettes (1958) on page 4, while explaining the German and British perspectives in the relevant literature. In the rest of the paragraph, the authors underline the absence of the same topic in the French context. The authors can refer here to the works of Beyza C. Tekin. They already refer to her in the following pages, but this section also deserves a citation. The authors also refer to the Ottoman Orientalist approach on page 9. Edhem Eldem's work on this should be useful and deserves a mention here.

 Finally, the issue of refugees has been one of the most important issues in Türkiye-EU relations over the past decade. The article does not sufficiently discuss this issue and does not show the relevant literature that analyses Türkiye-EU relations from the perspective of the refugee issue and its impact on identity discussions.

**CONCLUSION**

 All in all, the article is right in its criticism that Türkiye-EU relations are mostly studied in terms of how Europeans have represented Türkiye rather than Turkish representations of Europe. This point is important. There is a widespread perception that Turks are already eager to join the EU, based on the views of both the public and the state. This interpretation is based on questionnaires only. Social reality is not always clearly reflected in quantitative data. It is easy to observe in Türkiye that both state actors and the public are becoming more and more suspicious of the EU and its approach to Türkiye every year.

 In conclusion, this article is a great contribution and fills a gap in the field. I highly recommend its indexing.

**Some other notes:**

- Calling what happened in 1915 the "Armenian Genocide" (on pages 4 and 22) is polemical. I leave it to the authors to decide, but I personally do not accept this statement. I think it would be a more impartial approach to explain how different sides call the Armenian issue differently (e.g. Turkish official position, etc.).
- This is up to the authors, but all EU institutions now use the name "Türkiye".
- Page 11: "our work package" sounds like a project report.
- Page 29: Calling Özal and Demirel "Sunni-Islamicist" sounds controversial.
- Page 30: Negotiations began on 3 October 2005.

**Typos:**

 -Page 3: Decmber

 -Page 3: Turkish republic

 -Page 3: asu such

 -Page 4: issueand

 -Page 9: explaining

 -Page 14: these

 -Page 15: theythey

 -Page 17: embaced

 -Page 18: eras

 -Page 21: turkey

 -Page 24: Europe

 -Page 29: Euopean

 -Page 35: Greeece

 -Page 35: Yanyk

Are all factual statements correct and adequately supported by citations?

Yes

Is the review written in accessible language?

Yes

Are the conclusions drawn appropriate in the context of the current research literature?

Yes

Is the topic of the review discussed comprehensively in the context of the current literature?

Partly

Reviewer Expertise:

Journalism, Media studies, Türkiye-EU relations, European identity

I confirm that I have read this submission and believe that I have an appropriate level of expertise to confirm that it is of an acceptable scientific standard.

# Reviewer report: Reviewer response for version 1

**DOI:** 10.21956/openreseurope.17463.r34379

**Published:** 2023-08-29

**Authors:** Sahizer Samuk

**Affiliations:** 1 International Migration Research Centre, Wilfrid Laurier University, Waterloo, Ontario, Canada

I think that it is a timely and important publication. I have enjoyed reading it and it flows well.

 There are a few typos but small ones, with one careful read it shall be much better. For instance, Moslems (e.g. small mistakes as such)...

 I suggest the authors make it literature-wise richer, and add resources that support these claims, especially regarding the claims in these paragraphs:

 In terms of cultural developments, the period witnessed several major cultural shifts in the Ottoman Empire. Starting with the fashioning of the Ottoman bureaucratic system in the European style and the establishment of the translation bureau, Westernization in urban social and intellectual life spread gradually and geographically from Istanbul to other urban centres. Translations were made from European literature starting in the 1850s and Ottoman writers started experimenting with European genres of novel and poetic styles. Consumption patterns and leisure activities as well as architectural style and education imitated European models to varying degrees among the emerging Ottoman middle classes.

 Especially this paragraph needs resources to support the claims:

 The literature on the Ottoman perspectives on Europe and its identity dimension during the nineteenth century mainly focus on the tensions of Ottoman reform process: the desire to become a part of the post-Vienna European order and the formation of national identity which gradually acquires an anti-Westernist tone. Westernization appears as both a desirable phenomenon which involves acquisition of military administrative technology, and also as a social ill which manifests in consumption patterns, material culture and alienation. The literature on European perspective, on the other hand, focus mainly on two things: orientalist representations of the East as exotic and backward at the same time and Eastern question, the problem of the fate of the Ottoman Empire and its multi-confessional subjects in the face of Russian threat and subsequent nationalist uprisings particularly in the Balkans.

 Greene does not have the reference and the year... (it shall be added)

 Typo: However, they strategically aimed to either include Turkey in their political and military bloc or prevent it from sliding to orbit of their rivals ( Barlas & Güvenç, 2009: 435). --- they repeated twice

 When was Turkish opera established? (the year?)

 Please base this remark on resources: On one hand, Europe was viewed as everybody’s ‘project’, ‘promised land’ and ‘ideal’. It was also dismissed as violator of universal principles and Christian club responsible for the revitalization of Sèvres Syndrome in Turkish society. Towards the end of the final period, literature also stresses an increasing pace of de-Europeanization in Turkish politics in response to the loss of EU membership perspective.

 Is it true that Turkey has de Europeanised in response to loss of membership? Can this claim be true as a reason for de Europeanisation?

 Turkey is not written with a big letter in the title (in one of the titles). There are such mistakes all along …

 Conclusions:

 What is the original contribution of this paper? It is long and detailed and it is highly important to consider these perspectives in different periods but could this paper be shortened somehow to be more concise? Even if there is no such way, since I am not a historian I would suggest Cigdem Oguz, University of Bologna and Hazal Pabuccular Istanbul Kultur University  as reviewers, my real strength is not history.

 I also suggest Raymond Taras’s paper on xenophobia in Europe where the Turkish are depicted very much pejoratively in the literature. Maybe that can be added to your paper? Here is the link: https://www.tandfonline.com/doi/abs/10.1080/10848770902999492

 It is a wonderful paper that looks at the literary works and understands the xenophobia and fears in Europe in general.

 Finally, more details on methodology is needed. Which databases were used in Koc and Sabanci ? Some authors not familiar might not know anything about which kind of databases are included.

 I suggest some minor to major revisions.

 It is very useful and rigorous but it needs more details: methodology for instance, Sabanci and Koc premises include which databases?

 In which periods it has been made? Which papers were covered? Did they use xenophobia also as a keyword for instance, it happens that many papers which focus on xenophobia also focus on the perception of the third country nationals, including Turkish for the EU? 

 Why there is not the recent data of brain drain emigration authoritarianism?

 Is it the time limitation?

 Turkey is encountering serious problems regarding liberties, democracy and more… what kind of perception does this mean for Turkey from the view point of Europe? Where do we stand? I was wondering if it is intentional not to include the recent critical works.

 Thanks!

Are all factual statements correct and adequately supported by citations?

Partly

Is the review written in accessible language?

Yes

Are the conclusions drawn appropriate in the context of the current research literature?

Partly

Is the topic of the review discussed comprehensively in the context of the current literature?

Yes

Reviewer Expertise:

NA

I confirm that I have read this submission and believe that I have an appropriate level of expertise to confirm that it is of an acceptable scientific standard, however I have significant reservations, as outlined above.

## References

[1] : Transnational Xenophobia in Europe? Literary Representations of Contemporary Fears. *The European Legacy* .2009;14(4) : 10.1080/10848770902999492 391-407 10.1080/10848770902999492

# Reviewer report: Reviewer response for version 1

**DOI:** 10.21956/openreseurope.17463.r34121

**Published:** 2023-08-22

**Authors:** Beyza Çağatay Tekin

**Affiliations:** 1 Department of International Relations, Galatasaray University, Istanbul, İstanbul, Turkey

This article presents an analytical overview of Turkish perceptions of Europe and European perceptions of Turkey, covering a broad historical period from the time of the French Revolution to the 21st century. I liked the study's approach to periodisation, as its division into four distinct chronological phases provides a well-organised and clear framework for analysis.

 The article is a carefully crafted and well-organised analytical account that explores the representation of Self and Other within these two mutually constitutive entities. Overall, the article is an engaging and valuable scholarly contribution. Its significance extends to scholars specialising in the Self/Other nexus in International Relations, as well as to those concerned with understanding the dynamics of EU-Turkey relations. I believe it will be a reference for many of us interested in identity constructions and the politics of representation.

 The study has a twofold aim: first, to identify Turkish conceptualisations and interpretations of Europe, including the evolution of images and representations over time. Second, and arguably more challenging, is to identify the main attributes and themes that characterise European representations of Turkey. This undertaking, which focuses on change and continuity over many centuries, presents a Herculean challenge. The authors skillfully provide an exhaustive and vivid account of Turkish perceptions of Europe and European perspectives on Turkey, traversing four key dimensions or 'focal issues': civilisation, status in international society, nationalism, and state-citizen relations.

 In my view, the article succeeds in its first objective, which is to provide an insightful, analytical and more or less comprehensive review of studies on Turkish images and representations of Europe since the French Revolution. As for the second task, however, I believe that the sheer size of this vast, multilingual community makes it literally impossible to provide a comprehensive account of all scholarly work on Turkey's image in Europe. To address this, the authors tackle the problem by focusing their study on Turkish and English publications from two specific institutions: Sabancı and Koç University Libraries.

 The study's holistic approach potentially undermines the depth of its insights into the complexity of mutual images, representations and perceptions of Turkey in different parts of Europe. It is wonderful to see European images and representations of Turkey through three centuries in a single comprehensive analytical review, but this necessarily comes at the cost of losing valuable nuances. It would be useful if the authors could comment on how they deal with this potentially important loss of information due to the comprehensive scope of this long durée study.

 The study could also benefit from a theoretical perspective at the beginning or end to explain how mutual representations are shaped and evolve over time. Can we speak of a collective imaginary/memory or habitus that has existed in this part of the world we call 'Europe' from the late eighteenth century to the present day? Can we really speak of ‘the European’ representations of Turkey in the late eighteenth or early nineteenth century? Some European countries spent many centuries under Ottoman rule, others did not. Some sought a military alliance with the Ottoman Empire or Turkey against a common threat, others did not. So they are likely to have different memories and recollections, which are reflected in their images and representations of Turkey. Can we speak of similarities or divergent tendencies across the East-West or the South-North divide of the continent? These are some of the points that I think could be included in such a wonderful account of scholarship on the mutual representations of Europe and Turkey.

Are all factual statements correct and adequately supported by citations?

Yes

Is the review written in accessible language?

Yes

Are the conclusions drawn appropriate in the context of the current research literature?

Yes

Is the topic of the review discussed comprehensively in the context of the current literature?

Yes

Reviewer Expertise:

Identity in International Relations, EU-Turkey Relations, International Migration.

I confirm that I have read this submission and believe that I have an appropriate level of expertise to confirm that it is of an acceptable scientific standard.

# Reviewer report: Reviewer response for version 1

**DOI:** 10.21956/openreseurope.17463.r34120

**Published:** 2023-08-14

**Authors:** Catherine MacMillan

**Affiliations:** 1 Department of English Language and Literature, Yeditepe University, Istanbul, İstanbul, Turkey

The research project is based on a multidisciplinary literature review of studies of Turkish and European identity and their mutual perception starting from the French Revolution up to the mid 2010s. The project divides the review into 4 periods - the long 19th century, the early years of the Turkish Republic, the Cold War and early post-Cold war period, and the period between the recognition of Turkey's EU candidacy in 1999 and the impasse in Turkish/EU relations in 2016. It focuses on four main themes: civilisation, status in international society, nationalism, and state-citizen relations.

 In my view, the project has several strong points: it is thorough, focuses on the (relative) longue duree in Turkish/European relations, and is multidisciplinary. This has allowed Gülmez, Topal and Rumelili to identify some important gaps in the literature, including, for instance, a lack of research on how Ottoman or early Republican reforms affected the Turkish image in Europe, or how European responses to Turkish immigration in the third period affected the European image in Turkey. The study has, importantly, also allowed the authors to identify certain key moments and themes in the development of the image of the Turk in Europe and of Europe in Turkey, and has allowed them to identify continuities and variations over time in this regard. In short, this is an important and valuable metastudy of the research on Turkish/European images and relations. A minor criticism is that there are several typos, spelling mistakes (e.g. visa-versa) and grammatical errors (especially regarding the use of articles. Therefore, while the language is generally clear and coherent, these relatively minor issues should be dealt with. 

 In my view, although this is not part of the study, the article could also benefit from a short introductory paragraph(s) which would place it in context by discussing European/Turkish identity and images in the early Ottoman period..

Are all factual statements correct and adequately supported by citations?

Yes

Is the review written in accessible language?

Yes

Are the conclusions drawn appropriate in the context of the current research literature?

Yes

Is the topic of the review discussed comprehensively in the context of the current literature?

Yes

Reviewer Expertise:

Turkey-EU relations, discourse analysis, politics and literature

I confirm that I have read this submission and believe that I have an appropriate level of expertise to confirm that it is of an acceptable scientific standard.

# Reviewer report: Reviewer response for version 1

**DOI:** 10.21956/openreseurope.17463.r34381

**Published:** 2023-08-14

**Authors:** Digdem Soyaltin Colella

**Affiliations:** 1 Department of Politics and International Relations, University of Aberdeen, Aberdeen, Scotland, UK

This article delves into the evolution of mutual identity representations between Turkey and Europe, tracing their development and transformations over time. The comprehensive analysis yields valuable empirical insights, spanning over a span of two centuries. The delineated time frames (1789-1922, 1923-1945, 1946-1998, and 1999-2016) serve as a useful guide for readers, facilitating an understanding of how perceptions shifted in accordance with pivotal historical junctures in Turkey and/or Europe. The authors also explore key topics pertinent to identity discourse on both fronts, including nationalism, civilization, international status, and state-citizen dynamics. A short theoretical part could have been added after the introduction elaborating on the construction of identity. In its current form, the focus points discussed in the article appear somewhat detached from the prevailing literature and theoretical deliberations concerning the (self and other) identity constructions in socio-historical contexts. 

 Overall, this is a very well-written, empirical very rich academic work analytically reviewing the existing literature on and bringing novel perspectives to the complex and contested relations between Turkey and the West/Europe.

Are all factual statements correct and adequately supported by citations?

Yes

Is the review written in accessible language?

Yes

Are the conclusions drawn appropriate in the context of the current research literature?

Yes

Is the topic of the review discussed comprehensively in the context of the current literature?

Partly

Reviewer Expertise:

Turkey-EU relations, Europeanisation, Turkish politics, European politics

I confirm that I have read this submission and believe that I have an appropriate level of expertise to confirm that it is of an acceptable scientific standard.
